# A Library of *Aspergillus niger* Chassis Strains for Morphology Engineering Connects Strain Fitness and Filamentous Growth With Submerged Macromorphology

**DOI:** 10.3389/fbioe.2021.820088

**Published:** 2022-01-17

**Authors:** Timothy C. Cairns, Xiaomei Zheng, Claudia Feurstein, Ping Zheng, Jibin Sun, Vera Meyer

**Affiliations:** ^1^ Chair of Applied and Molecular Microbiology, Institute of Biotechnology, Technische Universität Berlin, Berlin, Germany; ^2^ Tianjin Institute of Industrial Biotechnology, Chinese Academy of Sciences, Tianjin, China; ^3^ Key Laboratory of Systems Microbial Biotechnology, Chinese Academy of Sciences, Tianjin, China; ^4^ University of Chinese Academy of Sciences, Beijing, China; ^5^ National Technology Innovation Center of Synthetic Biology, Tianjin, China

**Keywords:** *Aspergillus niger*, macromorphology, pellet, dispersed growth, genome editing, Tet-on, *pkh2*

## Abstract

Submerged fermentation using filamentous fungal cell factories is used to produce a diverse portfolio of useful molecules, including food, medicines, enzymes, and platform chemicals. Depending on strain background and abiotic culture conditions, different macromorphologies are formed during fermentation, ranging from dispersed hyphal fragments to approximately spherical pellets several millimetres in diameter. These macromorphologies are known to have a critical impact on product titres and rheological performance of the bioreactor. Pilot productivity screens in different macromorphological contexts is technically challenging, time consuming, and thus a significant limitation to achieving maximum product titres. To address this bottleneck, we developed a library of conditional expression mutants in the organic, protein, and secondary metabolite cell factory *Aspergillus niger*. Thirteen morphology-associated genes transcribed during fermentation were placed via CRISPR-Cas9 under control of a synthetic Tet-on gene switch. Quantitative analysis of submerged growth reveals that these strains have distinct and titratable macromorphologies for use as chassis during strain engineering programs. We also used this library as a tool to quantify how pellet formation is connected with strain fitness and filamentous growth. Using multiple linear regression modelling, we predict that pellet formation is dependent largely on strain fitness, whereas pellet Euclidian parameters depend on fitness and hyphal branching. Finally, we have shown that conditional expression of the putative kinase encoding gene *pkh2* can decouple fitness, dry weight, pellet macromorphology, and culture heterogeneity. We hypothesize that further analysis of this gene product and the cell wall integrity pathway in which it is embedded will enable more precise engineering of *A. niger* macromorphology in future.

## Background

Filamentous fungi are used to produce a diverse portfolio of molecules worth several billion dollars per year, including platform chemicals, proteins, enzymes, secondary metabolites, and organic acids (Meyer et al., 2016, 2020). The majority of industrially produced fungal metabolites are generated in stirred tank bioreactors which are often several hundred litres in volume (Meyer et al., 2021). Maximizing product titres from minimum substrate and energy inputs promises to both reduce environmental impacts of fermentation and limit costs, which may ultimately enable fungal biotechnology to strengthen bioeconomy supposed to replace the fossil fuel economy in the near future (Cairns et al., 2018; Meyer et al., 2020).

During submerged growth, filamentous fungi produce a range of different macromorphologies, including dispersed mycelial networks, loose clumps, spherical pellets several millimetres in diameter, or heterogenous mixtures of these structures (Cairns et al., 2019a; Meyer et al., 2021). The formation of respective macromorphologies is a complex process that is not comprehensively understood. In general, clumped and pelleted growth is thought to involve coagulation of spores or hyphal fragments, whereas dispersed mycelial growth occurs following low levels of cell coagulation, in addition to fragmentation of large aggregates during bioreactor stirring (Cairns et al., 2019a, 2021; Meyer et al., 2021). It is clear that the precise macromorphologies formed in submerged culture has critical implications for titres of proteins, enzymes, secondary metabolites, and acids (Zhang and Zhang, 2016; Veiter et al., 2018; Cairns et al., 2019a). Additionally, each growth morphology has advantages and limitations from a process engineering perspective. For example, the rheological consequences of dispersed mycelial growth are elevated medium viscosity, and consequently temperature/substrate concentration gradients (Žnidaršič and Pavko, 2001; Papagianni, 2004; Meyer et al., 2021). In contrast, clumped/pelleted macromorphologies improve transfer of oxygen and increase efficiency for removing biomass from growth media during downstream processing. However, clumped or pelleted fungal macromorphologies may result in hypoxia at internal pellet areas, thus limiting growth, metabolism, and possibly production of a desired molecule (Driouch et al., 2010).

Given the crucial role of macromorphological development in productivity and rheological performance, biotechnologists have modified numerous abiotic culture parameters in order to control their respective development, including stir speed (Papagianni, 2007), oxygen concentration (Wongwicharn et al., 1999), pH of growth media (Veiter et al., 2018), addition of Tween surfactants (Liu and Wu, 2012), carbon/nitrogen sources (Papagianni et al., 1999), manganese ions (Kisser et al., 1980; Berovic et al., 2006), addition of hydrous magnesium silicate particles (Wucherpfennig et al., 2012), spore inoculum concentrations (Papagianni and Mattey, 2006), and other methods (Kurt et al., 2018). These approaches may constitute additional challenges or energy input considerations for industrial-level fermentation, and consequently fungal strains with defined and reproducible macromorphological development in liquid culture are preferable.

In order to develop such strains, recent genetic and molecular engineering has generated isolates with modified macromorphological growth in a range of fungi, including targeted genetic manipulation of genes encoding putative GTPases, chitin synthases, and many others (Andersen et al., 2011; Fiedler et al., 2018a, 2018b; Sun et al., 2018). In some instances, morphology engineering has drastically elevated product titres. As one example, production of a glucoamylase enzyme was elevated by 400% by over-expressing the encoding *glaA* gene in a hyper-branching, dispersed mycelial *Aspergillus niger* chassis strain (Fiedler et al., 2018a).

Despite these advances, however, several challenges limit the design and interpretation of genetic-based morphological engineering studies. Firstly, it is unclear which genes should be prioritized for functional analyses indeed, filamentous fungal genomes typically consist of >10,000 predicted genes, with approximately a fifth of these putatively involved in hyphal morphology, growth, or development (Stajich et al., 2012). Even in model filamentous fungi (e.g., *Neurospora crassa* or *Aspergillis nidulans*), most genes lack wet-lab functional analysis, thus making selection of genes for analysis extremely challenging (Schäpe et al., 2019).

Secondly, genetic modifications will usually result in pleotropic phenotypic consequences, whereby any macromorphological change(s) occur concomitantly with defects in a given biological process or pathway (e.g., chitin synthesis, Golgi vesicle trafficking, actin polymerisation (Andersen et al., 2011; Fiedler et al., 2018a, 2018b; Sun et al., 2018; Cairns et al., 2019a)). It is therefore challenging to determine if elevated titres of a desired molecule are due to macromorphological optimization, alterations in regulatory pathways, metabolic processes, or a combination of these factors.

More generally, it is unclear to what extent fitness, i.e. the reproductive success of a population (Elena and Lenski, 2003), contributes to macromorphological development in liquid culture. It is therefore difficult to distinguish genes and cognate proteins/pathways which disrupt submerged growth due to their role in fitness (e.g., by simply producing a “sick” strain), and those that mechanistically control some aspect of macromorphological formation or maintenance (e.g., spore coagulation, hyphal fragmentation rates, etc). Quantitative estimations of the interconnectedness between fungal fitness, growth, and macromorphological development may therefore aid the identification of *bona fide* targets that mechanistically control macromorphological development, which could conceivably enable more precise control of submerged growth.

The filamentous fungus *A. niger* represents an outstanding host for macromorphological engineering due to its wide industrial applications for organic acid, protein, and secondary metabolite fermentation and the availability of an advanced genetic toolkit (Cairns et al., 2021). In this study, we aimed to overcome limitations and challenges for *A. niger* strain engineering by developing a suite of isolates with distinct, predefined, and user controllable macromorphologies. This strain library can be used as chassis expression hosts for rapidly screening optimal submerged growth-types for a desired product. Additionally, we quantified fitness and filamentous growth amongst the strain library. By integrating these data with submerged growth phenotypes, multiple linear regression models were used to give quantitative estimates for the interconnectedness of fitness, filamentous growth, and macromorphological development in *A. niger*. This study therefore provides numerous genetic leads for strain engineering, multiple chassis for facile identification of optimal growth morphologies, and quantitative estimates for how fitness and filamentous growth effect submerged macromorphologies.

## Materials and Methods

### Microbial Strains

Fungal strains used in this study are given in Table 1. MA70.15 was used as progenitor isolate (Meyer et al., 2007). All bacterial plasmids were propagated in *Escherichia coli* DH5α using 100 μg/ml ampicillin as selection.

**TABLE 1:** Strains and corresponding morphogenes characterized in this study. Morphogenes were divided into six categories based on their predicted function. *citA* coefficients give correlation values relative between this gene and the respective morphogene.

Gene name^a^	Gene	Paralog(s) in *A. niger* ^b^	Strain name (this study)	Predicted functional category	*citA* coeff	Function as described in yeast and filamentous fungi^c^	Evidence of morphogene function
CRN1	An02g01210	None	TC3.2	Cytoskeletal	0.80	Coronin; cortical actin cytoskeletal component that associates with the Arp2p/Arp3p complex to regulate its activity; plays a role in regulation of actin patch assembly	Mikati et al. (2015)
SPT8	An07g04000	None	TC2.1	SAGA complex	0.70	Subunit of the chromatin-modifying SAGA transcriptional regulatory complex	Gao et al. (2014)
CTI6	An01g10200	None	CAF25.4	SAGA complex	0.75	Component of the Rpd3L histone deacetylase complex that recruits the SAGA complex to repressed promoters	Han and Emr, (2013)
MTS1	An13g00740	An03g02040, An02g04530	TC14.2	Sphingolipid	0.70	Sphingolipid C9-methyltransferase important for cell membrane biosynthesis and signalling	Oura and Kajiwara, (2010)
SEC27	An02g05870	An16g02460	TC8.5	Golgi/vesicle	0.85	Component of the COPI coatomer; involved in ER-to-Golgi and Golgi-to-ER transport	Gabriely et al. (2007)
SEC26	An08g03270	None	TC10.1	Golgi/vesicle	0.75	Component of the COPI coatomer; involved in ER-to-Golgi protein trafficking and maintenance of normal ER morphology	DeRegis et al. (2008)
COG4	An02g14400	None	TC11.1	Golgi/vesicle	0.70	Component of the conserved oligomeric Golgi complex; a cytosolic tethering complex (Cog1p through Cog8p) that functions in protein trafficking to mediate fusion of transport vesicles to Golgi compartments	Gremillion et al. (2014)
TRS130	An08g05190	None	TC13.1	Golgi/vesicle	0.75	Component of transport protein particle (TRAPP) complex II; TRAPPII is a multimeric guanine nucleotide-exchange factor for the GTPase Ypt1p, regulating intra-Golgi and endosome-Golgi traffic	Pinar et al. (2015)
BRE5	An09g06580	None	TC16.1	Golgi/vesicle	0.75	Ubiquitin protease cofactor; forms deubiquitination complex with Ubp3p that coregulates anterograde and retrograde transport between the ER and Golgi compartments	Kang et al. (2016)
APL4	An01g02600	An14g00540	TC18.1	Endocytosis	0.75	Gamma-adaptin; large subunit of the clathrin-associated protein (AP-1) complex; binds clathrin; involved in vesicle-mediated transport	O’Donnell et al. (2010)
SWE1	An05g00280	None	CAF22.1	Kinase	0.75	Protein kinase that regulates the G2/M transition; morphogenesis checkpoint kinase; positive regulator of sphingolipid biosynthesis via Orm2p	Stefanini et al. (2018)
SIP2	An15g00910	None	TC24.1	Kinase	0.75	Subunit of the Snf1 kinase complex; involved in the response to glucose starvation	Sanz et al. (2016)
PKH2	An02g08630	An15g04520	TC17.1	Kinase	0.7	Protein kinase; involved in sphingolipid-mediated signalling pathway that controls endocytosis; activates Ypk1p and Ykr2p, components of signalling cascade required for maintenance of cell wall integrity	Friant et al. (2001)

a Nomenclature follows the nomenclature in *Saccharomyces cerevisiae*.

b Paralogues were identified from the Ensemble database.

c Note that most of these functions have been verified so far in *A. niger*.

### Media and Culture Conditions

Strains of *A. niger* were grown on at 30°C on solid minimal medium (MM) (Fiedler et al., 2018b). For submerged growth assays, CitACM liquid media consisted of 3 g/L (NH_4_)_2_SO_4_, 3 g/L NaNO_3_, 0.5 g/L yeast extract, and 100 g/L sucrose, with the pH adjusted to 2.5 using 100% HCl. Fermentation was conducted at 220 RPM, 34°C, for 96 h. All agar plates and liquid cultures had a concentration of 4 mM uridine.

### Coexpression Analysis

The *A. niger* coexpression networks were analysed using FungiDB (Stajich et al., 2012) for query gene *citA* (An09g06680). GO-enriched biological processes or cellular components in this list were identified relative to the *A. niger* genome using default parameters in FungiDB, with Benjamini-Hochberg FDR corrected *p*-values above 0.05 reported (Stajich et al., 2012; Schäpe et al., 2019).

### Molecular Techniques

All molecular techniques were performed according to standard procedures described previously (Fiedler et al., 2018b). *A. niger* transformation and genomic DNA extraction were performed as described elsewhere (Meyer et al., 2010), with 5–10 μg/ml doxycycline (Dox) added to primary transformation plates and sub-culture media.

CRISPR-mediated genome editing was conducted as described previously (Cairns et al., 2019b). All plasmid sequences will be made available on reasonable request. 2 µg of the Cas9 encoding plasmid Cas9-Hyg [Zheng et al., in preparation] was co-transformed with 2 µg purified sgRNA and donor constructs into *A. niger* MA70.15 protoplasts as previously described (Zheng et al., 2018). Following selection (200 μg/ml hygromycin and 5–10 μg/ml Dox) and duplicate purification (200 μg/ml hygromycin and 5–10 μg/ml Dox) on MM supplemented, genomic DNA was extracted from transformants. Insertion of the donor cassette at the respective promoter region was confirmed by diagnostic PCRs using verification primers (Supplementary File S1). Isolates generated in this study were confirmed for single integration of the Tet-on cassette at the target locus using Southern blot probe and the *fraA* promoter of the Tet-on cassette (Supplementary File S1, (Wanka et al., 2016),).

### Growth Quantification on Solid Media

Hyphal growth was measured on MM agar slices that were sufficiently thin (approx. 1 mm) for light microscopic analysis as described previously (Cairns et al., 2019b). Briefly, 10 µl of 1 × 10^4^ spores/ml of mutant or control isolates were spotted in duplicate onto the agar slice, air dried, and incubated at 30°C for 18 h after which images of fungal growth were captured using a Zeiss Axio Cam Mrc5 light microscope. All fungal morphologies were quantified for length, branch rate (length µm/number of branches) and tip numbers using ImageJ. Growth assays were repeated three times, with a minimum of 30 hyphae quantified per Dox concentration/strain.

Radial growth rates were quantified on MM agar (pH 5.6) or MM with pH adjusted to 3.5 using HCl. Inoculation of 10 µl volume of 1 × 10^6^ spores/ml was used, and growth rates between days 5 and 10 calculated with the indicated Dox concentration supplemented to the agar. Low pH growth coefficients for each strain/Dox concentration were calculated thus:

$$\frac{\mathrm{Radial}\mathrm{growth}\mathrm{mutant pH}3.5}{\mathrm{Radial}\mathrm{growth}\mathrm{progenitor pH}3.5}/\frac{\mathrm{Radial}\mathrm{growth}\mathrm{mutant pH}5.6}{\mathrm{Radial}\mathrm{growth}\mathrm{progenitor pH}5.6}$$

### Quantitative Assessment of Submerged Morphology

Cultures were analysed using an Olympus szx7 stereomicroscope connected to a Canon DS126251 camera as previously described (Cairns et al., 2019b). For image capture, approximately 5 ml of culture volume was poured into a 25 ml petri dish, after which morphologies were gently agitated with a pipette tip to ensure pellets were physically separated. For each sample, triplicate images were captured from randomly selected regions of the petri dish. Images were captured on a black background with lighting from above to illuminate fungal pellets. Triplicate replicates were conducted for each Dox/strain condition.

Fungal morphologies were quantified in ImageJ/Fiji using the morphology of dispersed and pelleted growth (MPD) plugin using default parameters (Cairns et al., 2019c). Dispersed morphologies were defined as any fungal structure with an area <500 μm^2^ and ≥95 μm^2^. Pellets were defined as any structure with an area ≥500 µm^2^. The following parameters were calculated for each fungal pellet: 1) area (µm^2^), 2) Feret’s diameter (maximum diameter of each structure, µm), 3) aspect ratio (maximum diameter/minimum diameter), 4) solidity. Morphology numbers (MNs) were calculated as described earlier (Wucherpfennig et al., 2011, 2012):

$$\mathrm{Morphology Number=}\frac{2\;\times \;\sqrt{Area}\times Solidity}{\sqrt{\pi }\;\times Feret^{'}s\;Diameter\;\times \;Aspect\;ratio}$$

We also manually calculated the extent of hyphal length at the pellet periphery, whereby the pellet core was identified by eye, and the length of hyphae extending from the core quantified using ImageJ. This length was normalized by dividing by the pellet area.

### Determination of Fungal Biomass

To determine fungal biomass after imaging, cultures were filtered through triple layered muslin gauze, washed in sterile water, pat dried between paper towels, and added to pre-weighed falcon tubes. Biomass was incubated at 50–65°C until dry (minimum of 24 h) after which dry weight was determined.

### Regression Analysis

Multiple linear regression modelling was conducted in Microsoft Excel. Indicated test variables were considered to impact the dependent variable when *p* < 0.05. Where passing this threshold, test variable coefficients and calculated intercept were used to predict dependent variable values for the respective strain/Dox condition. In order to determine the utility of the model, predicted values were plotted as a function of those observed in the study.

## Results

### Using Coexpression Networks to Identify High-Priority Targets for Morphology Engineering

We have previously mined *A. niger* coexpression networks to identify genes which control secondary metabolite and citric acid titres during submerged growth (Cairns et al., 2019b; Schäpe et al., 2019). This unbiased approach for generating leads for functional analysis enables the delineation of robustly coexpressed genes over hundreds of different cultivation conditions (Stajich et al., 2012; Schäpe et al., 2019). Given that the citric acid cycle of *A. niger* generates precursor molecules for the production of organic acids, amino acids, proteins and secondary metabolites as well as ATP for biosynthetic processes, we reasoned that genes which are 1) coexpressed with the citric acid synthase encoding gene *citA* and 2) predicted to play a role in filamentous growth or morphology are high priority candidates for controlling and understanding industrially relevant macromorphological development.

**FIGURE 1:**
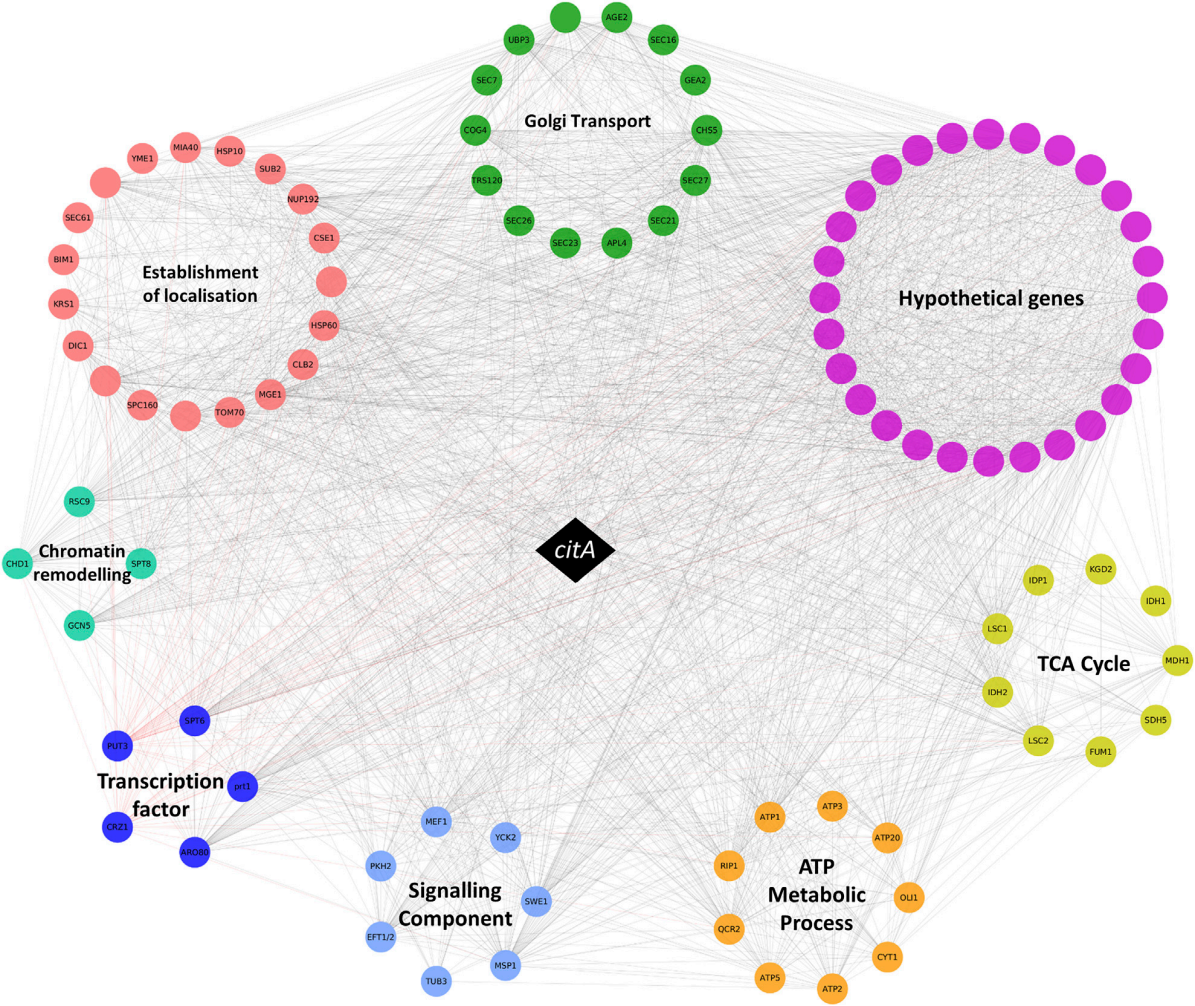
Schematic representation of the *citA* coexpression network. The *citA* gene is represented by a black diamond, and other genes are represented by coloured circles which are grouped into functional categories. Coexpression coefficients greater than 0.7 are depicted with grey lines. Gene names are given where available-genes lacking a name are blank, and full ORF codes are given in Supplementary Tables S1, S2.

We therefore interrogated the *A. niger citA* network, retrieving 249 candidate ORFs that were positively coexpressed with this gene from 283 microarray experiments (Spearman correlation coefficient ≥0.7, Supplementary Table S1). Consistent with the role of CitA in the Krebs cycle, the network was enriched with genes associated with tricarboxylic acid metabolism (GO:0072350, *p* < 0.0001) and citrate metabolism (GO:0006101, *p* < 0.0001, Figure 1 and Supplementary Table S2).

In order to identify morphology-associated genes embedded in this network, we interrogate enriched GO terms and manually predicted gene function(s) based on wet-lab experimentation for unicellular and multicellular fungi described in previous studies. Specifically, this suggested a total of 57 genes from the full 249 gene network are predicted to encode proteins that impact *A. niger* morphology, which we termed “morphogenes” (Supplementary Table S3). Note that the term is distinct from “morphogen”, which refers to chemical signals important for biological pattern formation, a term that was coined by Alan Turing in the 1950s (Green, 2021). We propose to use the term “morphogene” for genes and their protein products that play a central role in polarity establishment and polarity maintenance of fungal hyphae.

Notably, many morphogenes in the *citA* network are predicted to play a role in Golgi vesicle trafficking, a biological process vital for filamentous morphology whereby cell wall synthesizing proteins and membrane components are trafficked to the growing hyphal tip (Cairns et al., 2019c). This is supported by enrichment of the *citA* network with terms including intra-Golgi vesicle-mediated transport (GO:0006891, *p* < 0.01), COPI vesicle coat (GO:0030126, *p* < 0.01) and the TRAPPII protein complex (GO:1990071, *p* < 0.01, Supplementary Table S2). Other putative morphogenes included those encoding predicted signalling cascade components (e.g. putative kinases Swe1 and Pkh2 important for cell wall biosynthesis), cytoskeletal apparatus (e.g. predicted coronin Crn1) or chromatin remodelling complexes (e.g. Spt8 and Cti6), among others (Supplementary Table S3).

### Construction of a Morphogene Conditional Expression Library Using Genome Editing and a Tet-On Gene Switch

We selected 13 out of the 57 putative morphogenes to be studied in detail by loss-of function and gain-of-function analyses (Table 1). The selected candidate genes reflect the enrichment of Golgi associated processes and genes predicted to function in cell wall and cell membrane biosynthesis, chromatin remodelling and endocytosis, altogether processes which are central to hyphal growth in fungi (Steinberg et al., 2017). The MA70.15 strain was selected as a background for several reasons related to our intention for the library to be used as chassis strains in future strain engineering studies. Firstly, disruption of the *kusA* gene in MA70.15 enables high rates of homologous recombination with exogenous cassettes and the recipient genome (Meyer et al., 2007). This will enable facile strain engineering in the derivative strains. Secondly, MA70.15 is an orotidine-5′-phosphate decarboxylase deficient mutant (*pyrG* ^−^), and consequently the widely applied *pyrG* selection marker (Meyer et al., 2007) and, additionally, *pyrG* high expression locus (Schäpe et al., 2019) remains available in future engineering programs using these isolates. (Meyer et al., 2007; Schäpe et al., 2019).

Conditional expression mutants were generated as previously described, whereby a 20 bp locus in the 5’ UTR of the gene of interest was targeted using a sgRNA, and cut using a Cas9 nuclease (Supplementary File S1, (Zheng et al., 2018; Cairns et al., 2019b, 2019c)). 40-base pair regions targeted around the Cas9 cut site were used to replace the native promoter with the Tet-on conditional expression system (Wanka et al., 2016), thus placing the gene of interest under conditional control of this cassette (Supplementary File S1). Note that transformation plates were supplemented with Dox to ensure recovery of clones in which the gene of interest was essential (see Materials and Methods section). Transformants were screened using verification primers which spanned the insertion site of the Tet-on sequence, and single integration of the cassette was confirmed using Southern blot (Supplementary File S1 and Supplementary Table S4). A total of 13 strains corresponding to various morphogenes that passed PCR and Southern blot verification were further analysed (Table 1).

### Morphogene Titration During Submerged Growth Generates Multiple Chassis Strains for Interrogating Morphology and Productivity in *A. niger*

**FIGURE 2:**
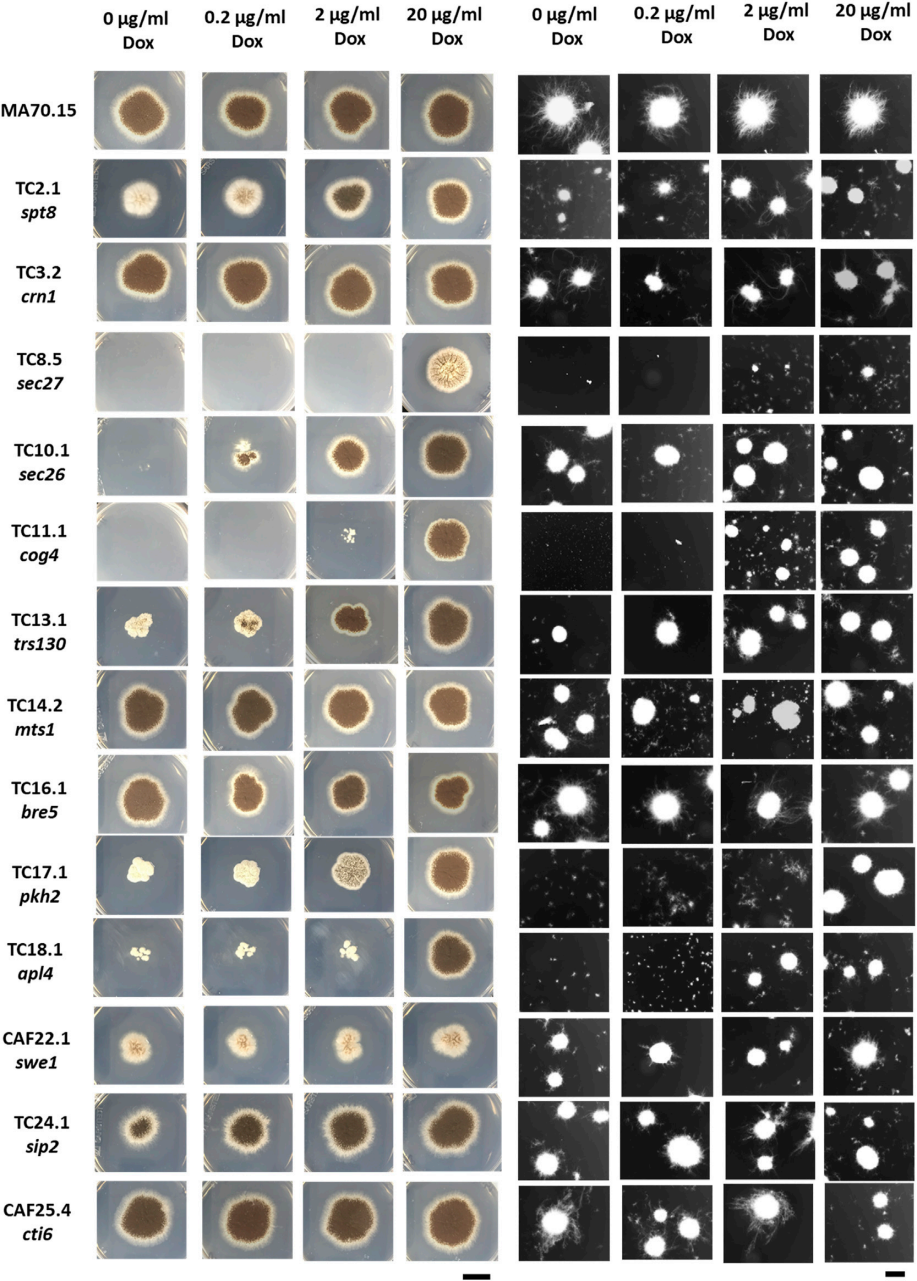
Solid and liquid growth of the morphogene library following titration using Dox. Colony development after 5 days growth at 30°C on solid MM (left panels). Scale bar = 1 cm. Representative images of submerged growth in CitACM media for 96 h at 220 RPM, 34°C (right panels). Scale bar = 1 mm.

In a preliminary growth assay, colony development of the conditional expression mutants were analysed on solid agar supplemented with 0, 0.2, 2 and 20 μg/ml Dox, equating to null, low, intermediate, or overexpression respectively. Various colony defects were observed for 9 out of 13 mutants at when Dox was omitted from media (TC2.1, TC8.5, TC10.1, TC11.1, TC13.1, TC17.1, TC18.1 CAF22.1, TC24.1, and Figure 2). Observed growth defects were titratable by increasing Dox concentration for all strains except isolate CAF22.1, although this mutant demonstrated some titration of conidiation when grown on MM supplemented with 0.2 M NaCl (Figure 2, and data not shown). Respective inability of strains TC8.5 and TC11.1 to grow on media lacking Dox demonstrate that genes *sec27* and *cog4* are essential in *A. niger*. This observation highlights the utility of the Tet-on conditional cassette, as analysis of *sec27* and *cog4* by conventional deletion approaches would not be possible. Growth of the s*ec26* conditional expression mutant (TC10.1) without Dox resulted in severe growth retardation, whereby development was only visible by close inspection of the plate. Based on this assay, we assumed that most mutants in the library would display titratable growth defects during submerged culture.

**FIGURE 3:**
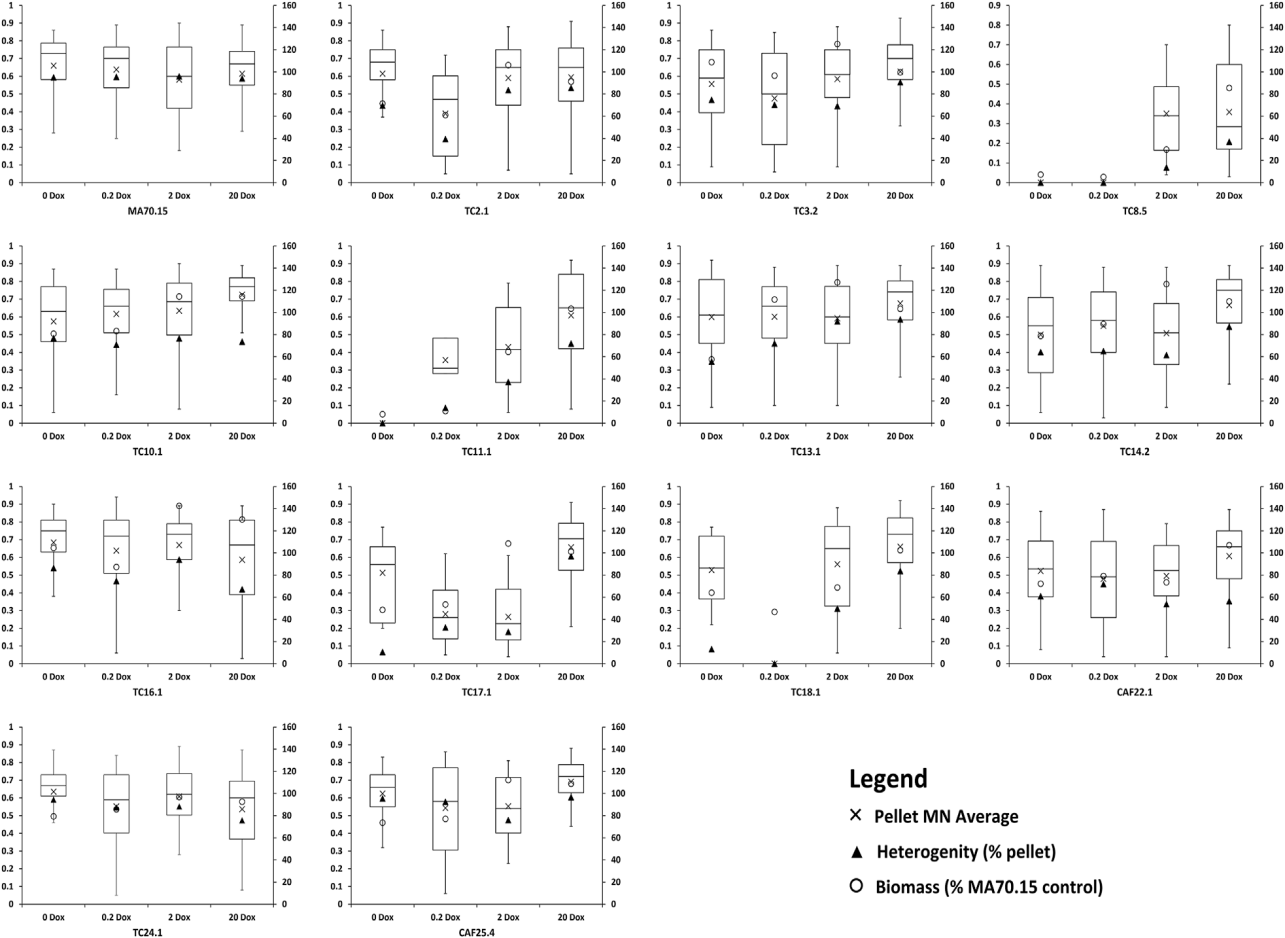
Quantification of culture heterogeneity, biomass, and pellet MNs following titration of respective morphogene expression during liquid fermentation. Left axis: MN, which vary between 0–1 and are represented by boxplots. Average MN values are depicted by a cross, with the middle horizontal line depicting the median MN. Right axis: Heterogeneity is given as a percentage of pellets for the total culture (see Materials and Methods section). Biomass is given as a percent of MA70.15 control at the respective Dox concentration.

In order to test this hypothesis, we modelled fermentation of the conditional expression mutants in commonly used submerged conditions using a standard complete medium that allows high citric acid production (CitACM, see Materials and Methods). Morphogene expression was titrated in shake flasks using the above four Dox concentrations, with representative images of culture macromorphologies shown (Figure 2). Macromorphological growth forms were quantified using the automated MPD image analysis plugin and are reported alongside culture dry weights and macromorphological heterogeneity at the end of fermentation (Figure 3). This analysis demonstrates a range of titratable culture parameters at both an individual strain level, and additionally throughout the library, including biomass, culture heterogeneity, and pellet Euclidian parameters, including the dimensionless morphology number (MN) (Figure 3), which varies between 0 (a one dimensional line, referring to a unbranched hyphae) and 1 (a perfect circle, referring to a perfect circular pellet; (Wucherpfennig et al., 2011)).

Two key macromorphologies that have received much research interest for morphological engineering are the degree of dispersed filamentous growth and pellet diameter (Wucherpfennig et al., 2011; Gonciarz and Bizukojc, 2014; Cairns et al., 2019a 2021). The library developed in this study delivers at least four options for strains with low levels of pellet production (e.g. 0–37% of the culture) with either elevated or reduced biomass (Table 2). Similarly, two exemplar instances where pellet diameter can be reduced (e.g. to ∼70% of the progenitor control) are given in Table 2, although many more possibilities are available throughout the library (Figures 2, 3). In all instances, titration of gene expression can be used to modify culture parameters to near-progenitor levels, thus enabling facile negative control cultures in macromorphological/productivity screens. For example, users wishing to test if dispersed growth elevates product titres could express a gene of interest in isolates TC17.1 and TC11.1. Growth at 2 μg/ml Dox will generate cultures with ∼70 and 60% dispersed growth, whereas control conditions with 20 μg/ml Dox will result in 4 and 30% dispersed growth, respectively. This library also allows determine and to adjust the degree of heterogeneity, i.e. the ratio between dispersed and pelleted macromorphologies, to study the impact of heterogeneity phenomena on the production of organic acids, proteins or secondary metabolites by *A. niger*.

**TABLE 2:** Exemplar macromorphological chassis options available from the morphogene library. Various options for dispersed morphologies, biomass, MN, and diameter are given.

Macromorphology chassis strain summary	Test condition		Parameter			
	Strain	Dox (µg/ml)	Biomass (% MA70.15 control)	% Pellet	MN	Pellet diameter (relative to MA70.15 control)
Dispersed macromorphology	TC17.1	2	108%	28%	0.26	85%
	TC8.5	20	85%	36%	0.35	80%
	TC11.1	2	64%	37%	0.42	70%
	TC18.1	0.2	46%	0%	—	—
Reduced pellet diameter	TC2.1	0	106%	69%	0.61	70%
	CAF22.1	0.2	79%	70%	0.47	71%

### Probing Interconnections Between Strain Fitness, Hyphal Growth, and Submerged Macromorphological Development

We noted a clear correlation between colony development on solid agar and pelleted growth in submerged culture (Figure 2), suggesting that strain fitness and defects in pellet formation are connected. In order quantify which (if any) aspects of strain fitness, i.e. hyphal growth are robustly correlated with submerged macromorphology, we further quantified the morphogene strain library following titration of gene expression. Firstly, as a broad measurement of strain fitness, we quantified colony radial growth rates which were reported as mm growth/24 h between days 5 and 10.

**FIGURE 4:**
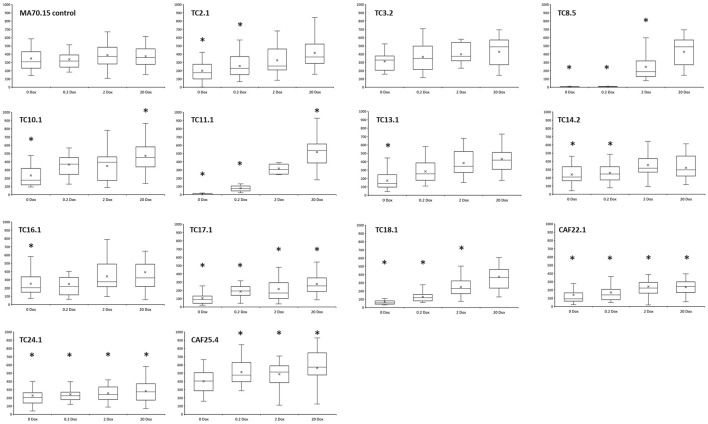
Quantification of hyphal length following growth on MM agar at 30°C for 18 h. Dox concentrations are indicated. Strains with values that significantly deviate from progenitor control at the respective Dox concentration are indicated with an asterisk (*t*-test). Approximately 25 hyphae per strain/Dox condition were analysed. *Y*-axis: Hyphal length [µm].

As a simple measurement of hyphal growth, recently germinated hyphae were analysed following 18 h incubation on solid agar, with length, branch rate, and number of hyphal tips quantified (Figure 4 and data not shown). The MA70.15 progenitor grew approximately 340 µm on all Dox concentrations, with an average of one branch generating a total of three tips (Figure 4). Among the library were numerous strains with significantly reduced hyphal length relative to control when growth media had Dox omitted (TC2.1, TC8.5, TC10.1, TC11.1, TC13.1, TC14.2, TC16.1, TC17.1, TC18.1, CAF22.1, and TC24.1). We could restore normal hyphal length by supplementing increased Dox concentrations into growth media in these strains (Figure 4). A single conditional expression strain with gene *cti6* (predicted to encode a component of SAGA histone deacetylase complex, Table 1) under control of Tet-on demonstrated increased hyphal growth rate relative control at 0.2, 2, and 20 μg/ml Dox, which is consistent with elevated dry weight in this strain in liquid culture at comparable Dox concentrations (Figure 2). Similarly, we found increased branching frequency and tip number in numerous strains throughout the library.

Finally, we reasoned that hyphal growth rates at low pH may be a crucial aspect of submerged macromorphological development of *A. niger* (Figure 2), especially for citric acid fermentation that occurs at low pH. We therefore quantified colony radial growth for each strain/Dox concentration when grown on MM agar with pH 3.5. From these data, we generated low pH growth coefficients by two normalization steps. Firstly, radial growth was normalized to the progenitor control at pH 3.5, and secondly to the growth between the mutant and control on standard MM agar (pH 5.6, Supplementary File S3). Several strains demonstrated that sensitivity to low pH at 0/0.2 μg/ml Dox, including mutants expressing *sec27* (strain TC8.5), *cog4* (strain TC11.1) and *pkh2* (strain TC17.1). Overexpression of *sec27* at 20 μg/ml Dox resulted in improved tolerance to lower pH. Additionally, expression of *trs130* in isolate TC13.1 at 0, 0.2, and 2 μg/ml Dox resulted in elevated resistance to low pH (Supplementary File S3). Taken together, we developed profiles of general strain fitness, hyphal morphology, and pH tolerance in each conditional expression mutant across four Dox concentrations.

**FIGURE 5:**
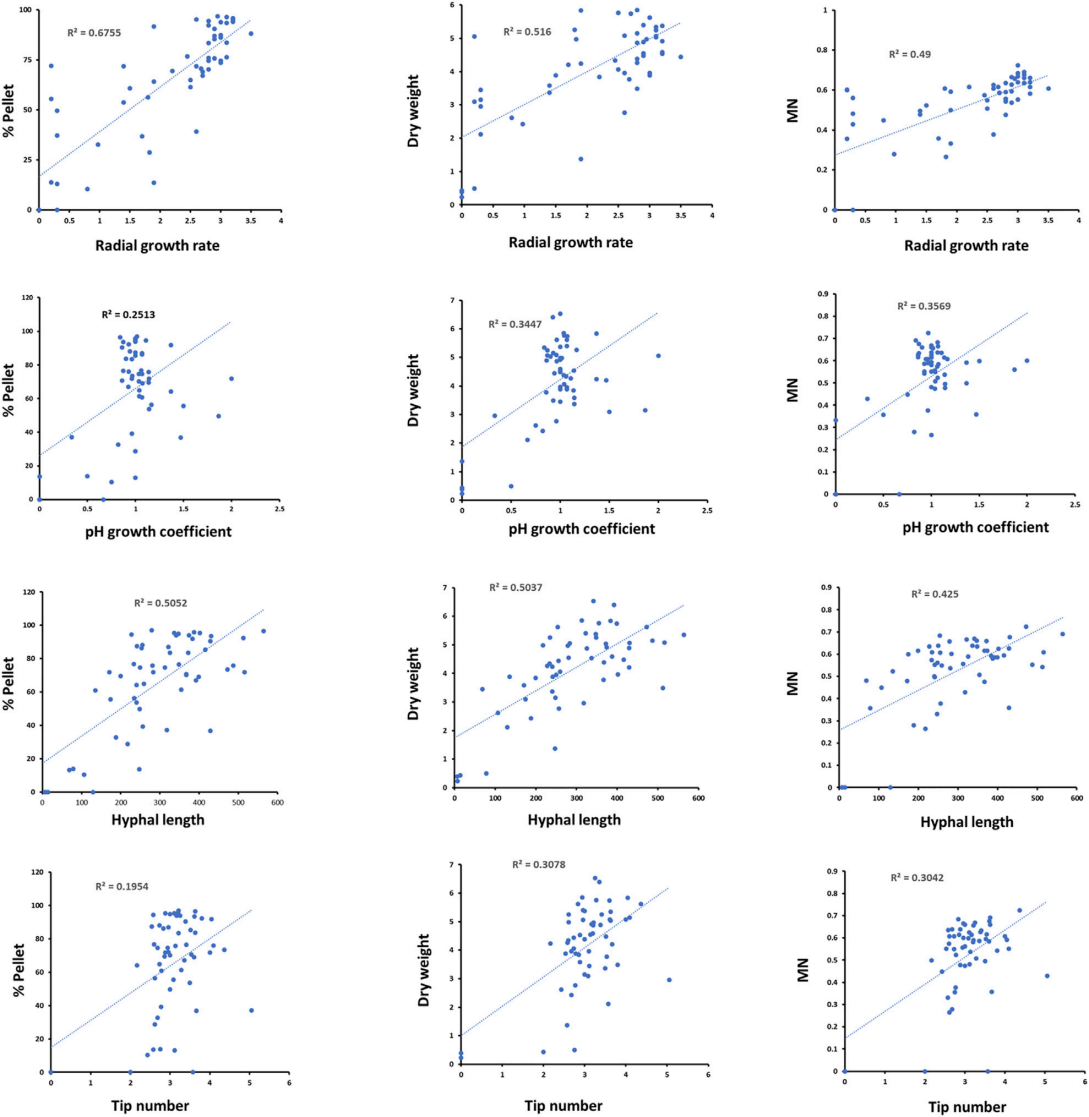
Pairwise correlations between strain radial growth rates, pH growth coefficients, hyphal length and tip number with submerged growth parameters. Each blue dot represents correlations between the indicated parameters at a specific strain/Dox concentration (*n* = 56). Line of best fit and *R* ^2^ values are given.

Next, we plotted the above measurements profiles against submerged culture parameters for each strain/Dox concentration (Figure 5). Amongst the comparisons, the strongest observed correlation was between strain radial growth rate and heterogeneity during liquid culture, whereby isolates with reduced radial growth tended to produce less pellets (*R* ^2^ = 0.67). Discernible positive correlations were also observed between radial growth and dry weight and MN (*R* ^2^ = 0.50 and 0.49, respectively). Moderate positive correlations were also observed between hyphal length and heterogeneity, dry weight, or MN (Figure 5). In general, pH growth coefficients and hyphal tip number were not well correlated with submerged growth (Figure 5), suggesting any cause/effect relationship between these growth parameters and submerged macromorphology is not detectable by simple pairwise comparisons.

### Multiple Linear Regression Modelling Connects Strain Fitness and Hyphal Growth With Submerged Macromorphological Development

**FIGURE 6:**
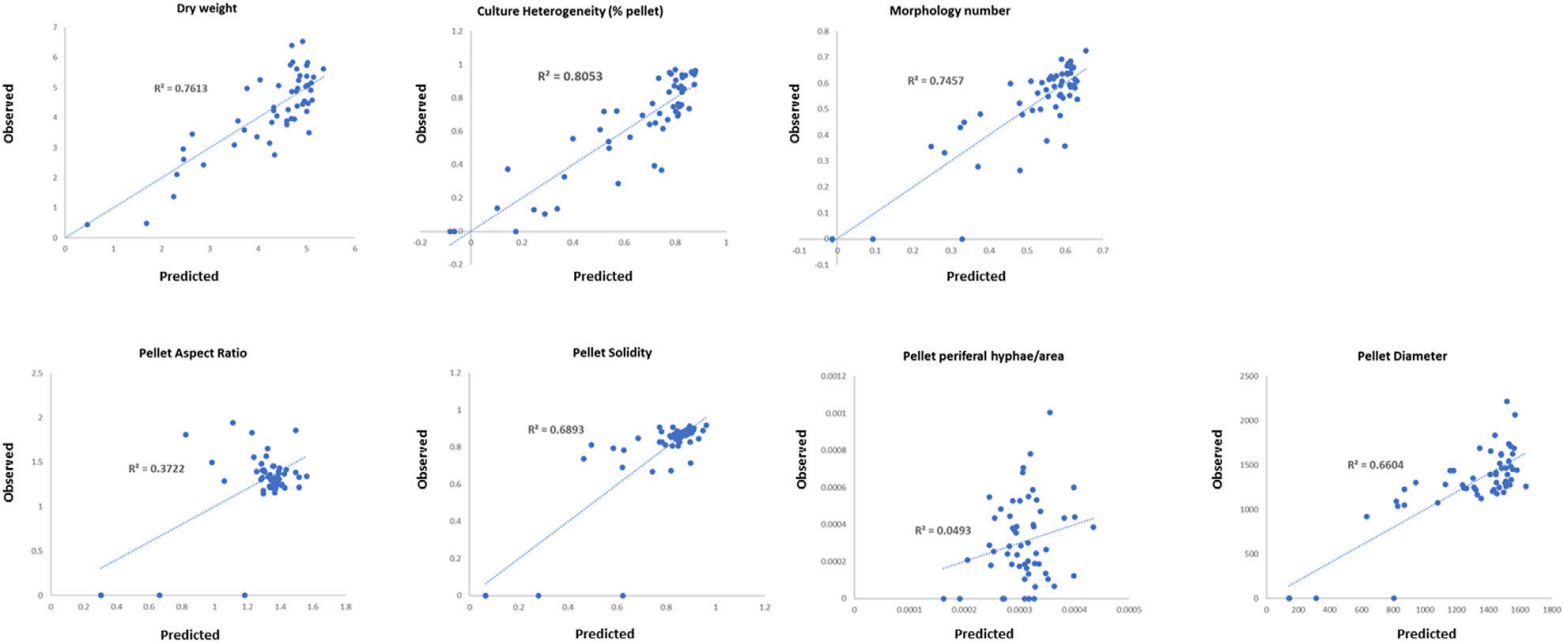
Multiple linear regression analysis of the conditional expression library. Correlations between values predicted for each strain/Dox concentration and those observed are given.

In order to further probe the interconnections between strain fitness, filamentous growth, and submerged culture, we conducted regression analyses whereby submerged growth parameters from the conditional expression library were used as dependent variables (% pellet, dry weight, MN, pellet solidity, pellet aspect ratio, pellet diameter, and pellet hyphal/core ratios) and measurements of fitness/hyphal growth were used as test variables (radial growth rate, hyphal length, hyphal tip number, and pH growth coefficient). Test variables were considered to significantly impact dependent variables when *p* < 0.05, and regression coefficients were used to predict the magnitude of the effect (Table 3). For each strain/Dox concentration, observed dependent variables were plotted as a function of those predicted by the regression model (Figure 6).

**TABLE 3:** Regression models used to predict how changes in strain fitness/hyphal growth impacted submerged macromorphologies. Test variables were only incorporated into the regression model where *p* < 0.05. Note that tip number was not predicted to significantly impact heterogeneity or dry weight.

Solid culture parameter	Change relative to wild type	Predicted change in submerged culture (% relative to wild type)			
		Pellet macromorphology		General growth aspects	
		Diameter	MN	Heterogeneity	Dry weight
Radial growth rate (mm/day)	25%	11	12	19	14
	50%	22	23	37	29
	75%	33	35	56	44
pH Adaptation Coefficient	25%	7	8	9	10
	50%	14	15	17	21
	75%	21	23	26	32
Average Tip No	25%	5	7	0	0
	50%	6	8	0	0
	75%	7	10	0	0

Pellet aspect ratio, solidity, and hyphal/core ratios demonstrated poor correlations between observed values and those predicted by the respective regression model (Figure 6), indicating that these aspects of pellet macromorphology cannot be predicted based on any of the test variables. Pellet diameter and MN showed reasonable correlations between observed/predicted values (Figure 6), with radial growth, pH growth coefficient, and average tip number all predicted to impact these variables (*p* < 0.05, Table 3). Culture dry weight and heterogeneity also demonstrated comparable concordance between observed/predicted values (*R* ^2^ = 0.76 and 0.80 respectively), yet were dependent on radial growth and pH growth coefficients alone, indicating that filamentous morphologies play a minor role in determining these aspects of liquid culture.

## Discussion

Filamentous fungal growth in submerged culture produces a range of macromorphologies, each of which have advantages and disadvantages from the perspective of product titres and process engineering (Cairns et al., 2019c). It is not currently possible to predict an optimal macromorphology for a specific product *a priori* (Meyer et al., 2021). Consequently, testing product titres in strains with distinct macromorphologies constitutes a major limitation to developing the next generation of ultra-efficient fungal cell factories (Cairns et al., 2021).

To address this issue, we developed a chassis strain library in *A. niger* using a titratable Tet-on cassette. Morphogenes were selected based on robust coexpression with the citric acid synthase encoding gene *citA*, therefore confirming their active expression during growth and primary metabolism of *A. niger* and thus during organic acid production. Given that many of the morphogenes are also putatively involved in vesicle trafficking at the Golgi, we are confident that they are also transcribed during protein secretion in *A. niger* and therefore useful as chassis for protein production studies. In general, the coupling of Golgi and TCA cycle at the transcriptional level provide further evidence that protein and organic acid production are more highly interconnected than previously thought (Cairns et al., 2019b).

The morphogene conditional expression library is a flexible tool for strain engineering, with multiple options for assaying product titres in chassis strains with various culture heterogeneities, dry weights, and pellet Euclidian parameters (Table 2). Different macromorphologies can be used as chassis expression hosts across numerous genetic backgrounds, several predicted biological processes, and at user defined gene expression levels. Given the well-established role of *A. niger* as a homologous and heterologous expression system (van der Straat et al., 2014; Steiniger et al., 2017; Boecker et al., 2018), the library can be used to test organic acid, secondary metabolite, and protein/enzymes in different morphological chassis.

Despite the need for modelling bioreactor fermentation conditions, the most common (and arguably still the highest-throughput) preliminary method for assaying *A. niger* strain productivity/macromorphological development remains shake flask culture in small volumes (reviewed in (Cairns et al., 2021)). We therefore conducted quantification of submerged growth in shake flasks using standard cultivation conditions, which can be easily extended in future studies to the multitude of conditions used by the *A. niger* community. Testing the chassis strains and regression models in other growth media will be simple for end users, with the MPD image analysis freely available as an ImageJ plugin (Cairns et al., 2019b).

This study also used the conditional expression library to interrogate and quantify the relationship between strain fitness, i.e. hyphal growth, and submerged macromorphology. We observed clear correlations between a simple measurement of fitness (colony radial growth, growth at low pH) and submerged macromorphology, in which strains with poor general fitness were also poor pellet formers (*p* < 0.001, *R* ^2^ = 0.80, Figure 6). Indeed, based on regression models a reduction in radial growth of 25% relative to the control strain is predicted to cause a 19% decrease in pellet formation (and conversely elevated amounts of dispersed mycelial growth, Table 3). Our data therefore suggest that among the 13 strains analysed in this study, most poor pellet formers were likely explainable by defective growth. We suggest that in future genetic screens for morphology engineering, mutants with defective submerged macromorphologies yet comparable radial growth rates to progenitor controls should be prioritized as they will be more likely to identify genes which mechanistically control pellet formation as opposed to general strain fitness. We predict that such control experiments during mutant analysis will more easily identify *bona fide* regulators of pellet formation in submerged culture.

One such example in this study followed intermediate expression of the putative kinase encoding gene *pkh2* (2 μg/ml Dox, strain TC17.1). Here, we observed decoupling of strain fitness, dry weight, culture heterogeneity, and MN numbers, which was rare among the conditional expression library. At 2 μg/ml Dox, TC17.1 demonstrated deviations between predicted (dry weight 3.7 g, pellet 58%, MN 0.48) and observed values (dry weight 4.9 g, pellet 29%, MN 0.26), respectively. Thus, submerged macromorphologies/growth rates following intermediate expression of *pkh2* are likely not explainable by defects in strain fitness or filamentous branch rates, leading us to hypothesize that this putative kinase may regulate a specific aspect of pellet formation or maintenance during liquid culture. In *Saccharomyces cerevisiae*, Pkh2 is required for maintenance of cell wall integrity (CWI, (Roelants et al., 2002)), a regulatory pathway known to be the ultimate determinant of the shape of fungal hyphae (Riquelme et al., 2018). In agreement, deletion of *pkh2* in *A. nidulans* results in strong defects in colony growth (De Souza et al., 2013), which we also observed in this study for *A. niger*. Our data is consistent with comparative genomic analyses between a mutagenized *A. niger* isolate that grew as dispersed mycelium (SH2) and pelleted control (CBS513.88, (Yin et al., 2014)), which found multiple non-synonymous SNPs in genes associated with CWI in strain SH2. Thus, this study supports growing evidence that genes of the fungal CWI pathway, including *pkh2*, are vital for controlling pellet formation.

Our analysis was also able to predict how fitness and hyphal growth impacted Euclidian parameters of pellets. As noted above, strain fitness was crucial for pellet macromorphology, but regression modelling also predicted that hyphal tip number had a minor but detectable impact on pellet diameter and MN (e.g., a 25% decrease in tip number relative to the control was predicted to reduce pellet diameter by 5%). Thus, our data suggest that formation of pellets is largely dependent on strain fitness, whereas pellet Euclidian parameters are affected mainly by strain fitness and to a limited extent on branch rates.

One possible confounding factor in our approach is that we measured filamentous branching on solid agar, which could conceivably be modified in submerged growth. In general, however, branching defects in *A. niger* mutants are highly reproducible between solid and liquid culture (Fiedler et al., 2018a). The use of solid and liquid culture demonstrates that it is possible to predict behaviour of submerged macromorphology from simple agar plates that are higher throughput when compared to shake flasks.

A further refinement of the regression model will be possible in the future, given that more data will become included that e.g. cover more conditions that likely impact submerged macromorphological development such as osmotic and shear stress during bioreactor conditions. With the strain library described in this study and the availability of further conditional expression mutants of the predicted 57 morphogenes present in the *citA* coexpression network this will be easily possible.

## Conclusion

We have developed a library of *A. niger* conditional expression mutants with distinct and titratable submerged macromorphologies for use a chassis during strain engineering programs. This library may be a useful resource that enables the identification of optimal macromorphological growth for elevated titres of useful molecules (organic acids, proteins, secondary metabolites) produced by *A. niger*. Quantitative analysis of this library suggests that pellet formation is highly connected with strain fitness, with most poor pellet formers being defective in hyphal growth. Using multiple linear regression modelling, we predict that pellet formation is dependent largely on fitness, whereas pellet Euclidian parameters depend on fitness and hyphal branching. Finally, we have shown that conditional expression of the putative kinase encoding gene *pkh2* can decouple fitness, dry weight, pellet macromorphology, and culture heterogeneity. We hypothesize that further analysis of this gene and encoded protein will enable more precise engineering of *A. niger* macromorphology in the future.

## Data Availability

The original contributions presented in the study are included in the article/Supplementary Material, further inquiries can be directed to the corresponding authors.

## Abbreviations

Dox, Doxycycline; GO, Gene Ontology; MPD, Morphology of Pelleted and Dispersed Growth; MN, Morphology Number.
