# Supplementary material for: A Library of Aspergillus niger Chassis Strains for Morphology Engineering Connects Strain Fitness and Filamentous Growth With Submerged Macromorphology
Source: Front Bioeng Biotechnol. 2022 Jan 17;9:820088. doi: 10.3389/fbioe.2021.820088 (PMC8801610; doi:10.3389/fbioe.2021.820088)
Supplement: Supplementary file 2 [file DataSheet3.pdf]

A library of *Aspergillus niger* chassis strains for morphology engineering connects strain fitness and filamentous growth with submerged macromorphology

Timothy C. Cairns[1], Xiaomei Zheng[2.3.4.5]Claudia Feurstein[1], Ping Zheng[2,3,4,5], Jibin Sun[2,3,4,5], and Vera Meyer[1]

[1]Technische Universität Berlin, Institute of Biotechnology, Chair of Applied and Molecular Microbiology, Straße des 17. Juni 135, 10623 Berlin, Germany
[2] Tianjin Institute of Industrial Biotechnology, Chinese Academy of Sciences, Tianjin, 300308, People's Republic of China
[3] Key Laboratory of Systems Microbial Biotechnology, Chinese Academy of Sciences, Tianjin 300308, People's Republic of China
[4] University of Chinese Academy of Sciences, Beijing, 100049 China
[5] College of Biotechnology, Tianjin University of Science & Technology, Tianjin, 300457 China

Timothy C. Cairns: t.cairns@tu-berlin.de ORCID: 0000-0001-7106-224X
Xiaomei Zheng: zheng_xm@tib.cas.cn ORCID: 0000-0001-9136-0666
Claudia Feurstein: c.feurstein@tu-berlin.de: ORCID: 0000-0001-7046-4183
Ping Zheng: zheng_p@tib.cas.cn: ORCID: 0000-0001-9434-9892
Jibin Sun: sun_jb@tib.cas.cn: ORCID: 0000-0002-0208-504X
Vera Meyer: vera.meyer@tu-berlin.de, ORCID 0000-0002-2298-2258

Contact details for corresponding authors:

Timothy C. Cairns, Tel.: +49 30 314 72750, Fax: +49 30 314 72922, E-mail: t.cairns@tu-berlin.de

Jibin Sun, Tel.: +86-8486 1949, Fax: +86-8486 1943, E-mail: sun_jb@tib.cas.cn

Vera Meyer, Tel.: +49 30 314 72750, Fax: +49 30 314 72922, E-mail: vera.meyer@tu-berlin.de

Supplementary File 3

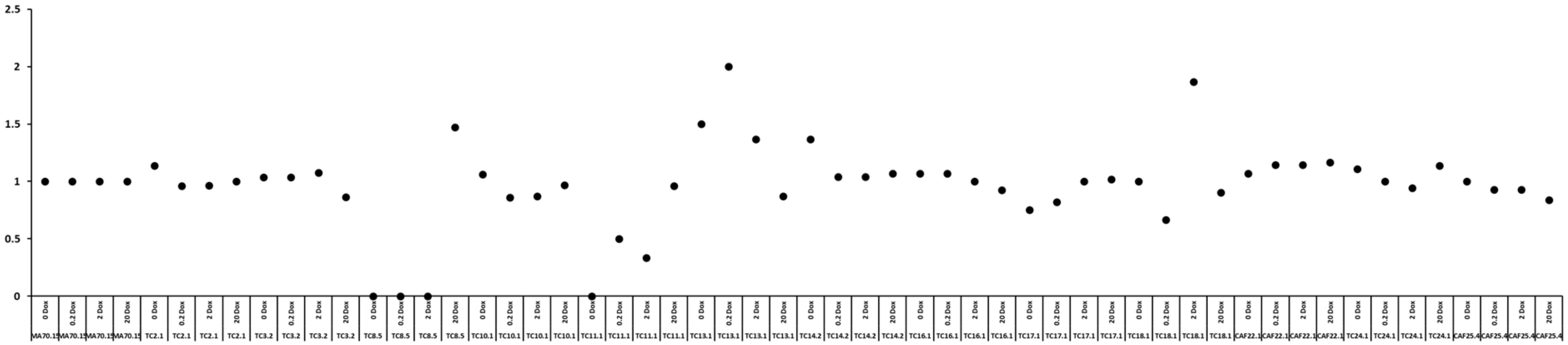

Growth coefficients at low pH. Colony radial growth rates were calculated from day 5-10 on MM (pH5.6) and MM (pH 3.5). Coefficients were calculated to reflect growth differences between mutant and control strain growth at low pH (3.5) vs standard MM (pH 5.6, see Materials and Methods section). Thus, a value of 1 indicates no change between mutant and control in either condition, <1 indicates poor growth of the conditional mutant at low pH, and >1 indicates improved growth. Zero values for strain TC8.5 (0, 0.2 µg/ml Dox) and TC11.1 at (0 µg/ml Dox) reflect no growth of these mutants at either pH.
