# Supplementary material for: A Library of Aspergillus niger Chassis Strains for Morphology Engineering Connects Strain Fitness and Filamentous Growth With Submerged Macromorphology
Source: Front Bioeng Biotechnol. 2022 Jan 17;9:820088. doi: 10.3389/fbioe.2021.820088 (PMC8801610; doi:10.3389/fbioe.2021.820088)
Supplement: Supplementary file 4 [file Table3.DOCX]

| **Gene** | **Spearman** | **Description** | **Name in Scer** | **Description SGD** | **Relevant literature** |
| --- | --- | --- | --- | --- | --- |
| An01g08160  (gcnE) | 0,7 | Ortholog(s) have histone acetyltransferase activity (H3-K14 specific), histone acetyltransferase activity (H3-K9 specific), lysine-acetylated histone binding activity | GCN5 | Catalytic subunit of ADA and SAGA histone acetyltransferase complexes; modifies N-terminal lysines on histones H2B and H3; acetylates Rsc4p, a subunit of the RSC chromatin-remodeling complex, altering replication stress tolerance; relocalizes to the cytosol in response to hypoxia; mutant displays reduced transcription elongation in the G-less-based run-on (GLRO) assay; greater involvement in repression of RNAPII-dependent transcription than in activation | [Genetics.](https://www.ncbi.nlm.nih.gov/pubmed/24907261) 2014 Aug;197(4):1175-89.  **The histone acetyltransferase GcnE (GCN5) plays a central role in the regulation of Aspergillus asexual development.**  [Cánovas D](https://www.ncbi.nlm.nih.gov/pubmed/?term=C%C3%A1novas%20D%5BAuthor%5D&cauthor=true&cauthor_uid=24907261)1, [Marcos AT](https://www.ncbi.nlm.nih.gov/pubmed/?term=Marcos%20AT%5BAuthor%5D&cauthor=true&cauthor_uid=24907261)2, [Gacek A](https://www.ncbi.nlm.nih.gov/pubmed/?term=Gacek%20A%5BAuthor%5D&cauthor=true&cauthor_uid=24907261)3, [Ramos MS](https://www.ncbi.nlm.nih.gov/pubmed/?term=Ramos%20MS%5BAuthor%5D&cauthor=true&cauthor_uid=24907261)2, [Gutiérrez G](https://www.ncbi.nlm.nih.gov/pubmed/?term=Guti%C3%A9rrez%20G%5BAuthor%5D&cauthor=true&cauthor_uid=24907261)2, [Reyes-Domínguez Y](https://www.ncbi.nlm.nih.gov/pubmed/?term=Reyes-Dom%C3%ADnguez%20Y%5BAuthor%5D&cauthor=true&cauthor_uid=24907261)3, [Strauss J](https://www.ncbi.nlm.nih.gov/pubmed/?term=Strauss%20J%5BAuthor%5D&cauthor=true&cauthor_uid=24907261)4.  Acetylation of histones is a key regulatory mechanism of gene expression in eukaryotes. GcnE is an acetyltransferase of Aspergillus nidulans involved in the acetylation of histone H3 at lysine 9 and lysine 14. Previous works have demonstrated that deletion of gcnE results in defects in primary and secondary metabolism. Here we unveil the role of GcnE in development and show that a ∆gcnE mutant strain has minor growth defects but is impaired in normal conidiophore development. No signs of conidiation were found after 3 days of incubation, and immature and aberrant conidiophores were found after 1 week of incubation. Centroid linkage clustering and principal component (PC) analysis of transcriptomic data suggest that GcnE occupies a central position in Aspergillus developmental regulation and that it is essential for inducing conidiation genes. GcnE function was found to be required for the acetylation of histone H3K9/K14 at the promoter of the master regulator of conidiation, brlA, as well as at the promoters of the upstream developmental regulators of conidiation flbA, flbB, flbC, and flbD (fluffy genes). However, analysis of the gene expression of brlA and the fluffy genes revealed that the lack of conidiation originated in a complete absence of brlA expression in the ∆gcnE strain. Ectopic induction of brlA from a heterologous alcA promoter did not remediate the conidiation defects in the ∆gcnE strain, suggesting that additional GcnE-mediated mechanisms must operate. Therefore, we conclude that GcnE is the only nonessential histone modifier with a strong role in fungal development found so far. |

| **Gene** | **Spearman** | **Description** | **Name in Scer** | **Description SGD** | **Relevant literature** |
| --- | --- | --- | --- | --- | --- |
| An07g04000 | 0,7 | Ortholog(s) have role in positive regulation of induction of conjugation with cellular fusion by regulation of transcription from RNA polymerase II promoter and SAGA complex, nuclear chromatin localization | SPT8 | Transcription factor,  subunit of the SAGA transcriptional regulatory complex; not present in SAGA-like complex SLIK/SALSA; required for SAGA-mediated inhibition at some promoters | [FEMS Microbiol Lett.](https://www.ncbi.nlm.nih.gov/pubmed/24289742) 2014 Feb;351(1):42-50.  **Transcription factors spt3 and spt8 are associated with conidiation, mycelium growth, and pathogenicity in Fusarium graminearum**.  [Gao T](https://www.ncbi.nlm.nih.gov/pubmed/?term=Gao%20T%5BAuthor%5D&cauthor=true&cauthor_uid=24289742)1, [Zheng Z](https://www.ncbi.nlm.nih.gov/pubmed/?term=Zheng%20Z%5BAuthor%5D&cauthor=true&cauthor_uid=24289742), [Hou Y](https://www.ncbi.nlm.nih.gov/pubmed/?term=Hou%20Y%5BAuthor%5D&cauthor=true&cauthor_uid=24289742), [Zhou M](https://www.ncbi.nlm.nih.gov/pubmed/?term=Zhou%20M%5BAuthor%5D&cauthor=true&cauthor_uid=24289742).  Fusarium graminearum (teleomorph: Gibberella zeae), the dominant pathogen of Fusarium head blight (FHB) on wheat, can cause substantial economic losses. The Spt-Ada-Gcn5-acetyltransferase (SAGA) transcription coactivator plays multiple roles in regulating transcription because of the presence of functionally independent modules of subunits within the complex. The transcription factors spt3 and spt8 are components of the SAGA complex and they are important in yeasts and filamentous fungi including F. graminearum. In this study, we identified Fgspt3 and Fgspt8, homologs of Saccharomyces cerevisiae spt3 and spt8 from F. graminearum using the blastp program. The aim of the present study was to investigate the functions of Fgspt3 and Fgspt8 in F. graminearum. The deletion mutants grew significantly more slowly than the wild-type parent and did not produce conidia. Expression of the sporulation-related genes FgFlbC and FgRen1 were significantly down-regulated in the mutants. The mutants exhibited no sexual reproduction on infected wheat kernels and a 90% decrease in virulence on wheat. Pigment formation was also greatly altered in the mutants. All of the defects were restored by genetic complementation of the mutant with wild-type Fgspt3 and Fgspt8 genes. Overall, Fgspt3 and Fgspt8 are essential genes in F. graminearum. |
| **Gene** | **Spearman** | **Description** | **Name in Scer** | **Description SGD** | **Relevant literature** |
| An02g01210 | 0,8 | Ortholog(s) have role in actin cytoskeleton organization, cell wall organization, conidium formation, hyphal growth, regulation of endocytosis, spore germination | CRN1 | Coronin; cortical actin cytoskeletal component that associates with the Arp2p/Arp3p complex to regulate its activity; plays a role in regulation of actin patch assembly | [Curr Protein Pept Sci.](https://www.ncbi.nlm.nih.gov/pubmed/26916159) 2016;17(6):603-11. The **Coronin Family and Human Disease**. [Liu X](https://www.ncbi.nlm.nih.gov/pubmed/?term=Liu%20X%5BAuthor%5D&cauthor=true&cauthor_uid=26916159)1, [Gao Y](https://www.ncbi.nlm.nih.gov/pubmed/?term=Gao%20Y%5BAuthor%5D&cauthor=true&cauthor_uid=26916159), [Lin X](https://www.ncbi.nlm.nih.gov/pubmed/?term=Lin%20X%5BAuthor%5D&cauthor=true&cauthor_uid=26916159), [Li L](https://www.ncbi.nlm.nih.gov/pubmed/?term=Li%20L%5BAuthor%5D&cauthor=true&cauthor_uid=26916159), [Han X](https://www.ncbi.nlm.nih.gov/pubmed/?term=Han%20X%5BAuthor%5D&cauthor=true&cauthor_uid=26916159), [Liu J](https://www.ncbi.nlm.nih.gov/pubmed/?term=Liu%20J%5BAuthor%5D&cauthor=true&cauthor_uid=26916159).  The Coronin family is one of the WD-repeat domain containing families that are diverse in both of their structures and functions. The first coronin was identified in the cytoskeleton composition of Dictyostelium discoideum, which was discovered to regulate the actin functions. So far, 723 coronins have been identified throughout the eukaryotic kingdom by bioinformatics analysis in 358 species. In mammals, 7 coronins have been identified to date, which are named through Coronin 1 to Coronin 7; all of these isoforms contain two structurally conservational region: a 7-bladed β-propeller scaffold in N-terminal and a C-terminal variable coiled coil domain. Although some studies were showing that mammalian coronins have regulated the actin dynamics, recently many other functions such as calcium signaling regulation, cAMP signaling regulation, have been also reported beyond the actin modulation. Furthermore, many diseases have been found to be extensively associated with the abnormal expression of coronins, such as auto-immunity, bacterial and virus infection, neuronal behavior disorder and cancer. In this review, we would like to systematically discuss the recent progresses of mammalian coronins and associated diseases, as well as possible underlying molecular mechanisms. |

| **Gene** | **Spearman** | **Description** | **Name in Scer** | **Description SGD** | **Relevant literature** |
| --- | --- | --- | --- | --- | --- |
| An07g02190 | 0,7 | Putative guanine nucleotide exchange factor; expression enhanced by maltose | SEC7 | Guanine nucleotide exchange factor (GEF) for ADP ribosylation factors; involved in proliferation of the Golgi, intra-Golgi transport and ER-to-Golgi transport; found in the cytoplasm and on Golgi-associated coated vesicles | [PLoS Genet.](https://www.ncbi.nlm.nih.gov/pubmed/29608571) 2018 Apr 2;14(4):e1007291.  **Endocytic recycling via the TGN underlies the polarized hyphal mode of life.** [Hernández-González M](https://www.ncbi.nlm.nih.gov/pubmed/?term=Hern%C3%A1ndez-Gonz%C3%A1lez%20M%5BAuthor%5D&cauthor=true&cauthor_uid=29608571)1, [Bravo-Plaza I](https://www.ncbi.nlm.nih.gov/pubmed/?term=Bravo-Plaza%20I%5BAuthor%5D&cauthor=true&cauthor_uid=29608571)1, [Pinar M](https://www.ncbi.nlm.nih.gov/pubmed/?term=Pinar%20M%5BAuthor%5D&cauthor=true&cauthor_uid=29608571)1, [de Los Ríos V](https://www.ncbi.nlm.nih.gov/pubmed/?term=de%20Los%20R%C3%ADos%20V%5BAuthor%5D&cauthor=true&cauthor_uid=29608571)2, [Arst HN Jr.](https://www.ncbi.nlm.nih.gov/pubmed/?term=Arst%20HN%20Jr.%5BAuthor%5D&cauthor=true&cauthor_uid=29608571)3, [Peñalva MA](https://www.ncbi.nlm.nih.gov/pubmed/?term=Pe%C3%B1alva%20MA%5BAuthor%5D&cauthor=true&cauthor_uid=29608571)1.  Intracellular traffic in Aspergillus nidulans hyphae must cope with the challenges that the high rates of apical extension (1μm/min) and the long intracellular distances (>100 μm) impose. Understanding the ways in which the hyphal tip cell coordinates traffic to meet these challenges is of basic importance, but is also of considerable applied interest, as fungal invasiveness of animals and plants depends critically upon maintaining these high rates of growth. Rapid apical extension requires localization of cell-wall-modifying enzymes to hyphal tips. By combining genetic blocks in different trafficking steps with multidimensional epifluorescence microscopy and quantitative image analyses we demonstrate that polarization of the essential chitin-synthase ChsB occurs by indirect endocytic recycling, involving delivery/exocytosis to apices followed by internalization by the sub-apical endocytic collar of actin patches and subsequent trafficking to TGN cisternae, where it accumulates for ~1 min before being re-delivered to the apex by a RAB11/TRAPPII-dependent pathway. Accordingly, ChsB is stranded at the TGN by Sec7 inactivation but re-polarizes to the apical dome if the block is bypassed by a mutation in geaAgea1 that restores growth in the absence of Sec7. That polarization is independent of RAB5, that ChsB predominates at apex-proximal cisternae, and that upon dynein impairment ChsB is stalled at the tips in an aggregated endosome indicate that endocytosed ChsB traffics to the TGN via sorting endosomes functionally located upstream of the RAB5 domain and that this step requires dynein-mediated basipetal transport. It also requires RAB6 and its effector GARP (Vps51/Vps52/Vps53/Vps54), whose composition we determined by MS/MS following affinity chromatography purification. Ablation of any GARP component diverts ChsB to vacuoles and impairs growth and morphology markedly, emphasizing the important physiological role played by this pathway that, we propose, is central to the hyphal mode of growth. |

| **Gene** | **Spearman** | **Description** | **Name in Scer** | **Description SGD** | **Relevant literature** |
| --- | --- | --- | --- | --- | --- |
| An11g02650 | 0,7 | Putative ARF GTPase activating protein effector; expression enhanced by maltose | AGE2 | ADP-ribosylation factor (ARF) GTPase activating protein (GAP) effector; involved in Trans-Golgi-Network (TGN) transport; contains C2C2H2 cysteine/histidine motif | [Mol Biol Cell.](https://www.ncbi.nlm.nih.gov/pubmed/12134061) 2002 Jul;13(7):2193-206.  **Activity of specific lipid-regulated ADP ribosylation factor-GTPase-activating proteins is required for Sec14p-dependent Golgi secretory function in yeast.**  [Yanagisawa LL](https://www.ncbi.nlm.nih.gov/pubmed/?term=Yanagisawa%20LL%5BAuthor%5D&cauthor=true&cauthor_uid=12134061)1, [Marchena J](https://www.ncbi.nlm.nih.gov/pubmed/?term=Marchena%20J%5BAuthor%5D&cauthor=true&cauthor_uid=12134061), [Xie Z](https://www.ncbi.nlm.nih.gov/pubmed/?term=Xie%20Z%5BAuthor%5D&cauthor=true&cauthor_uid=12134061), [Li X](https://www.ncbi.nlm.nih.gov/pubmed/?term=Li%20X%5BAuthor%5D&cauthor=true&cauthor_uid=12134061), [Poon PP](https://www.ncbi.nlm.nih.gov/pubmed/?term=Poon%20PP%5BAuthor%5D&cauthor=true&cauthor_uid=12134061), [Singer RA](https://www.ncbi.nlm.nih.gov/pubmed/?term=Singer%20RA%5BAuthor%5D&cauthor=true&cauthor_uid=12134061), [Johnston GC](https://www.ncbi.nlm.nih.gov/pubmed/?term=Johnston%20GC%5BAuthor%5D&cauthor=true&cauthor_uid=12134061), [Randazzo PA](https://www.ncbi.nlm.nih.gov/pubmed/?term=Randazzo%20PA%5BAuthor%5D&cauthor=true&cauthor_uid=12134061), [Bankaitis VA](https://www.ncbi.nlm.nih.gov/pubmed/?term=Bankaitis%20VA%5BAuthor%5D&cauthor=true&cauthor_uid=12134061).  Yeast phosphatidylinositol transfer protein (Sec14p) coordinates lipid metabolism with protein-trafficking events. This essential Sec14p requirement for Golgi function is bypassed by mutations in any one of seven genes that control phosphatidylcholine or phosphoinositide metabolism. In addition to these "bypass Sec14p" mutations, Sec14p-independent Golgi function requires phospholipase D activity. The identities of lipids that mediate Sec14p-dependent Golgi function, and the identity of the proteins that respond to Sec14p-mediated regulation of lipid metabolism, remain elusive. We now report genetic evidence to suggest that two ADP ribosylation factor-GTPase-activating proteins (ARFGAPs), Gcs1p and Age2p, may represent these lipid-responsive elements, and that Gcs1p/Age2p act downstream of Sec14p and phospholipase D in both Sec14p-dependent and Sec14p-independent pathways for yeast Golgi function. In support, biochemical data indicate that Gcs1p and Age2p ARFGAP activities are both modulated by lipids implicated in regulation of Sec14p pathway function. These results suggest ARFGAPs are stimulatory factors required for regulation of Golgi function by the Sec14p pathway, and that Sec14p-mediated regulation of lipid metabolism interfaces with the activity of proteins involved in control of the ARF cycle. |

| **Gene** | **Spearman** | **Description** | **Name in Scer** | **Description SGD** | **Relevant literature** |
| --- | --- | --- | --- | --- | --- |
| An18g02490  (geaA) | 0,7 | Putative guanine nucleotide exchange factor; expression enhanced by maltose | GEA2 | Guanine nucleotide exchange factor for ADP ribosylation factors (ARFs); involved in vesicular transport between the Golgi and ER, Golgi organization, and actin cytoskeleton organization; GEA1 has a paralog, GEA2, that arose from the whole genome duplication | [Mol Biol Cell.](https://www.ncbi.nlm.nih.gov/pubmed/28978742) 2017 Dec 1;28(25):3660-3671.  **Regulation of Arf activation occurs via distinct mechanisms at early and late Golgi compartments.**  [Gustafson MA](https://www.ncbi.nlm.nih.gov/pubmed/?term=Gustafson%20MA%5BAuthor%5D&cauthor=true&cauthor_uid=28978742)1, [Fromme JC](https://www.ncbi.nlm.nih.gov/pubmed/?term=Fromme%20JC%5BAuthor%5D&cauthor=true&cauthor_uid=28978742)2.  At the Golgi complex, the biosynthetic sorting center of the cell, the Arf GTPases are responsible for coordinating vesicle formation. The Arf-GEFs activate Arf GTPases and are therefore the key molecular decision-makers for trafficking from the Golgi. In Saccharomyces cerevisiae, three conserved Arf-GEFs function at the Golgi: Sec7, Gea1, and Gea2. Our group has described the regulation of Sec7, the trans-Golgi Arf-GEF, through autoinhibition, positive feedback, dimerization, and interactions with a suite of small GTPases. However, we lack a clear understanding of the regulation of the early Golgi Arf-GEFs Gea1 and Gea2. Here we demonstrate that Gea1 and Gea2 prefer neutral over anionic membrane surfaces in vitro, consistent with their localization to the early Golgi. We illustrate a requirement for a critical mass of either Gea1 or Gea2 for cell growth under stress conditions. We show that the C-terminal domains of Gea1 and Gea2 toggle roles in the cytosol and at the membrane surface, preventing membrane binding in the absence of a recruiting interaction but promoting maximum catalytic activity once recruited. We also identify the small GTPase Ypt1 as a recruiter for Gea1 and Gea2. Our findings illuminate core regulatory mechanisms unique to the early Golgi Arf-GEFs. |

| **Gene** | **Spearman** | **Description** | **Name in Scer** | **Description SGD** | **Relevant literature** |
| --- | --- | --- | --- | --- | --- |
| An07g06030 | 0,85 | COPI complex, gamma subunit; expression enhanced by maltose | SEC21 | Gamma subunit of coatomer; coatomer is a heptameric protein complex that together with Arf1p forms the COPI coat; involved in ER to Golgi transport of selective cargo | [Mol Biol Cell.](https://www.ncbi.nlm.nih.gov/pubmed/11294905) 2001 Apr;12(4):1035-45.  **The ADP ribosylation factor-nucleotide exchange factors Gea1p and Gea2p have overlapping, but not redundant functions in retrograde transport from the Golgi to the endoplasmic reticulum.**  [Spang A](https://www.ncbi.nlm.nih.gov/pubmed/?term=Spang%20A%5BAuthor%5D&cauthor=true&cauthor_uid=11294905)1, [Herrmann JM](https://www.ncbi.nlm.nih.gov/pubmed/?term=Herrmann%20JM%5BAuthor%5D&cauthor=true&cauthor_uid=11294905), [Hamamoto S](https://www.ncbi.nlm.nih.gov/pubmed/?term=Hamamoto%20S%5BAuthor%5D&cauthor=true&cauthor_uid=11294905), [Schekman R](https://www.ncbi.nlm.nih.gov/pubmed/?term=Schekman%20R%5BAuthor%5D&cauthor=true&cauthor_uid=11294905).  The activation of the small ras-like GTPase Arf1p requires the action of guanine nucleotide exchange factors. Four Arf1p guanine nucleotide exchange factors have been identified in yeast: Sec7p, Syt1p, Gea1p, and its homologue Gea2p. We identified GEA2 as a multicopy suppressor of a sec21-3 temperature-sensitive mutant. SEC21 encodes the gamma-subunit of coatomer, a heptameric protein complex that together with Arf1p forms the COPI coat. GEA1 and GEA2 have at least partially overlapping functions, because deletion of either gene results in no obvious phenotype, whereas the double null mutant is inviable. Conditional mutants defective in both GEA1 and GEA2 accumulate endoplasmic reticulum and Golgi membranes under restrictive conditions. The two genes do not serve completely overlapping functions because a Deltagea1 Deltaarf1 mutant is not more sickly than a Deltaarf1 strain, whereas Deltagea2 Deltaarf1 is inviable. Biochemical experiments revealed similar distributions and activities for the two proteins. Gea1p and Gea2p exist both in membrane-bound and in soluble forms. The membrane-bound forms, at least one of which, Gea2p, can be visualized on Golgi structures, are both required for vesicle budding and protein transport from the Golgi to the endoplasmic reticulum. In contrast, Sec7p, which is required for protein transport within the Golgi, is not required for retrograde protein trafficking. |

| **Gene** | **Spearman** | **Description** | **Name in Scer** | **Description SGD** | **Relevant literature** |
| --- | --- | --- | --- | --- | --- |
| An02g05870 | 0,85 | COPI complex, beta' subunit; expression enhanced by maltose | SEC27 | Essential beta'-coat protein of the COPI coatomer; involved in ER-to-Golgi and Golgi-to-ER transport; contains WD40 domains that mediate cargo selective interactions; 45% sequence identity to mammalian beta'-COP | [Mol Cell Biol.](https://www.ncbi.nlm.nih.gov/pubmed/17101773) 2007 Jan;27(2):526-40. Epub 2006 Nov 13.  **Involvement of specific COPI subunits in protein sorting from the late endosome to the vacuole in yeast.**  [Gabriely G](https://www.ncbi.nlm.nih.gov/pubmed/?term=Gabriely%20G%5BAuthor%5D&cauthor=true&cauthor_uid=17101773)1, [Kama R](https://www.ncbi.nlm.nih.gov/pubmed/?term=Kama%20R%5BAuthor%5D&cauthor=true&cauthor_uid=17101773), [Gerst JE](https://www.ncbi.nlm.nih.gov/pubmed/?term=Gerst%20JE%5BAuthor%5D&cauthor=true&cauthor_uid=17101773).  Although COPI function on the early secretory pathway in eukaryotes is well established, earlier studies also proposed a nonconventional role for this coat complex in endocytosis in mammalian cells. Here we present results that suggest an involvement for specific COPI subunits in the late steps of endosomal protein sorting in Saccharomyces cerevisiae. First, we found that carboxypeptidase Y (CPY) was partially missorted to the cell surface in certain mutants of the COPIB subcomplex (COPIb; Sec27, Sec28, and possibly Sec33), which indicates an impairment in endosomal transport. Second, integral membrane proteins destined for the vacuolar lumen (i.e., carboxypeptidase S [CPS1]; Fur4, Ste2, and Ste3) accumulated at an aberrant late endosomal compartment in these mutants. The observed phenotypes for COPIb mutants resemble those of class E vacuolar protein sorting (vps) mutants that are impaired in multivesicular body (MVB) protein sorting and biogenesis. Third, we observed physical interactions and colocalization between COPIb subunits and an MVB-associated protein, Vps27. Together, our findings suggest that certain COPI subunits could have a direct role in vacuolar protein sorting to the MVB compartment. |

| **Gene** | **Spearman** | **Description** | **Name in Scer** | **Description SGD** | **Relevant literature** |
| --- | --- | --- | --- | --- | --- |
| An01g04730 | 0,75 | COPII complexn subunit; expression enhanced by maltose | SEC23 | GTPase-activating protein, stimulates the GTPase activity of Sar1p; component of the Sec23p-Sec24p heterodimer of the COPII vesicle coat, involved in ER to Golgi transport; substrate of Ubp3/Bre5 complex; ubiquitylated by Ub-ligase Rsp5p; proteasome-mediated degradation of Sec23p is regulated by Cdc48p | [Nature.](https://www.ncbi.nlm.nih.gov/pubmed/21532587) 2011 May 12;473(7346):181-6.  **Sequential interactions with Sec23 control the direction of vesicle traffic.**  [Lord C](https://www.ncbi.nlm.nih.gov/pubmed/?term=Lord%20C%5BAuthor%5D&cauthor=true&cauthor_uid=21532587)1, [Bhandari D](https://www.ncbi.nlm.nih.gov/pubmed/?term=Bhandari%20D%5BAuthor%5D&cauthor=true&cauthor_uid=21532587), [Menon S](https://www.ncbi.nlm.nih.gov/pubmed/?term=Menon%20S%5BAuthor%5D&cauthor=true&cauthor_uid=21532587), [Ghassemian M](https://www.ncbi.nlm.nih.gov/pubmed/?term=Ghassemian%20M%5BAuthor%5D&cauthor=true&cauthor_uid=21532587), [Nycz D](https://www.ncbi.nlm.nih.gov/pubmed/?term=Nycz%20D%5BAuthor%5D&cauthor=true&cauthor_uid=21532587), [Hay J](https://www.ncbi.nlm.nih.gov/pubmed/?term=Hay%20J%5BAuthor%5D&cauthor=true&cauthor_uid=21532587), [Ghosh P](https://www.ncbi.nlm.nih.gov/pubmed/?term=Ghosh%20P%5BAuthor%5D&cauthor=true&cauthor_uid=21532587), [Ferro-Novick S](https://www.ncbi.nlm.nih.gov/pubmed/?term=Ferro-Novick%20S%5BAuthor%5D&cauthor=true&cauthor_uid=21532587).  How the directionality of vesicle traffic is achieved remains an important unanswered question in cell biology. The Sec23p/Sec24p coat complex sorts the fusion machinery (SNAREs) into vesicles as they bud from the endoplasmic reticulum (ER). Vesicle tethering to the Golgi begins when the tethering factor TRAPPI binds to Sec23p. Where the coat is released and how this event relates to membrane fusion is unknown. Here we use a yeast transport assay to demonstrate that an ER-derived vesicle retains its coat until it reaches the Golgi. A Golgi-associated kinase, Hrr25p (CK1δ orthologue), then phosphorylates the Sec23p/Sec24p complex. Coat phosphorylation and dephosphorylation are needed for vesicle fusion and budding, respectively. Additionally, we show that Sec23p interacts in a sequential manner with different binding partners, including TRAPPI and Hrr25p, to ensure the directionality of ER-Golgi traffic and prevent the back-fusion of a COPII vesicle with the ER. These events are conserved in mammalian cells. |

| **Gene** | **Spearman** | **Description** | **Name in Scer** | **Description SGD** | **Relevant literature** |
| --- | --- | --- | --- | --- | --- |
| An08g03270 | 0,75 | COPI complex, beta subunit; expression enhanced by maltose | SEC26 | Essential beta-coat protein of the COPI coatomer; involved in ER-to-Golgi protein trafficking and maintenance of normal ER morphology; shares 43% sequence identity with mammalian beta-coat protein (beta-COP) | [BMC Cell Biol.](https://www.ncbi.nlm.nih.gov/pubmed/18211691) 2008 Jan 22;9:3. doi: 10.1186/1471-2121-9-3.  **Mutational analysis of betaCOP (Sec26p) identifies an appendage domain critical for function.**  [DeRegis CJ](https://www.ncbi.nlm.nih.gov/pubmed/?term=DeRegis%20CJ%5BAuthor%5D&cauthor=true&cauthor_uid=18211691)1, [Rahl PB](https://www.ncbi.nlm.nih.gov/pubmed/?term=Rahl%20PB%5BAuthor%5D&cauthor=true&cauthor_uid=18211691), [Hoffman GR](https://www.ncbi.nlm.nih.gov/pubmed/?term=Hoffman%20GR%5BAuthor%5D&cauthor=true&cauthor_uid=18211691), [Cerione RA](https://www.ncbi.nlm.nih.gov/pubmed/?term=Cerione%20RA%5BAuthor%5D&cauthor=true&cauthor_uid=18211691), [Collins RN](https://www.ncbi.nlm.nih.gov/pubmed/?term=Collins%20RN%5BAuthor%5D&cauthor=true&cauthor_uid=18211691).  BACKGROUND:  The appendage domain of the gammaCOP subunit of the COPI vesicle coat bears a striking structural resemblance to adaptin-family appendages despite limited primary sequence homology. Both the gammaCOP appendage domain and an equivalent region on betaCOP contain the FxxxW motif; the conservation of this motif suggested the existence of a functional appendage domain in betaCOP.  RESULTS:  Sequence comparisons in combination with structural prediction tools show that the fold of the COOH-terminus of Sec26p is strongly predicted to closely mimic that of adaptin-family appendages. Deletion of the appendage domain of Sec26p results in inviability in yeast, over-expression of the deletion construct is dominant negative and mutagenesis of this region identifies residues critical for function. The ArfGAP Glo3p was identified via suppression screening as a potential downstream modulator of Sec26p in a manner that is independent of the GAP activity of Glo3p but requires the presence of the COOH-terminal ISS motifs.  CONCLUSION:  Together, these results indicate an essential function for the predicted betaCOP appendage and suggest that both COPI appendages perform a biologically active regulatory role with a structure related to adaptin-family appendage domains. |

| **Gene** | **Spearman** | **Description** | **Name in Scer** | **Description SGD** | **Relevant literature** |
| --- | --- | --- | --- | --- | --- |
| An02g14400  (SwoP) | 0,7 | Ortholog(s) have role in autophagy of peroxisome, establishment of cell polarity, filamentous growth of a population of unicellular organisms, macroautophagy and protein localization by the Cvt pathway, more | COG4 | Essential component of the conserved oligomeric Golgi complex; a cytosolic tethering complex (Cog1p through Cog8p) that functions in protein trafficking to mediate fusion of transport vesicles to Golgi compartments | [Fungal Genet Biol.](https://www.ncbi.nlm.nih.gov/pubmed/25312861) 2014 Dec;73:69-82.  **Mutations in proteins of the Conserved Oligomeric Golgi Complex affect polarity, cell wall structure, and glycosylation in the filamentous fungus Aspergillus nidulans.**  [Gremillion SK](https://www.ncbi.nlm.nih.gov/pubmed/?term=Gremillion%20SK%5BAuthor%5D&cauthor=true&cauthor_uid=25312861)1, [Harris SD](https://www.ncbi.nlm.nih.gov/pubmed/?term=Harris%20SD%5BAuthor%5D&cauthor=true&cauthor_uid=25312861)2, [Jackson-Hayes L](https://www.ncbi.nlm.nih.gov/pubmed/?term=Jackson-Hayes%20L%5BAuthor%5D&cauthor=true&cauthor_uid=25312861)3, [Kaminskyj SG](https://www.ncbi.nlm.nih.gov/pubmed/?term=Kaminskyj%20SG%5BAuthor%5D&cauthor=true&cauthor_uid=25312861)4, [Loprete DM](https://www.ncbi.nlm.nih.gov/pubmed/?term=Loprete%20DM%5BAuthor%5D&cauthor=true&cauthor_uid=25312861)3, [Gauthier AC](https://www.ncbi.nlm.nih.gov/pubmed/?term=Gauthier%20AC%5BAuthor%5D&cauthor=true&cauthor_uid=25312861)5, [Mercer S](https://www.ncbi.nlm.nih.gov/pubmed/?term=Mercer%20S%5BAuthor%5D&cauthor=true&cauthor_uid=25312861)6, [Ravita AJ](https://www.ncbi.nlm.nih.gov/pubmed/?term=Ravita%20AJ%5BAuthor%5D&cauthor=true&cauthor_uid=25312861)5, [Hill TW](https://www.ncbi.nlm.nih.gov/pubmed/?term=Hill%20TW%5BAuthor%5D&cauthor=true&cauthor_uid=25312861)6.  We have described two Aspergillus nidulans gene mutations, designated podB1 (polarity defective) and swoP1 (swollen cell), which cause temperature-sensitive defects during polarization. Mutant strains also displayed unevenness and abnormal thickness of cell walls. Un-polarized or poorly-polarized mutant cells were capable of establishing normal polarity after a shift to a permissive temperature, and mutant hyphae shifted from permissive to restrictive temperature show wall and polarity abnormalities in subsequent growth. The mutated genes (podB=AN8226.3; swoP=AN7462.3) were identified as homologues of COG2 and COG4, respectively, each predicted to encode a subunit of the multi-protein COG (Conserved Oligomeric Golgi) Complex involved in retrograde vesicle trafficking in the Golgi apparatus. Down-regulation of COG2 or COG4 resulted in abnormal polarization and cell wall staining. The GFP-tagged COG2 and COG4 homologues displayed punctate, Golgi-like localization. Lectin-blotting indicated that protein glycosylation was altered in the mutant strains compared to the wild type. A multicopy expression experiment showed evidence for functional interactions between the homologues COG2 and COG4 as well as between COG2 and COG3. To date, this work is the first regarding a functional role of the COG proteins in the development of a filamentous fungus. |

| **Gene** | **Spearman** | **Description** | **Name in Scer** | **Description SGD** | **Relevant literature** |
| --- | --- | --- | --- | --- | --- |
| An15g00470  (hypA) | 0,75 | Ortholog(s) have role in establishment or maintenance of cell polarity, hyphal growth and TRAPPII protein complex localization | TRS120 | Component of transport protein particle (TRAPP) complex II; TRAPPII is a multimeric guanine nucleotide-exchange factor for the GTPase Ypt1p, regulating intra-Golgi and endosome-Golgi traffic | [Proc Natl Acad Sci U S A.](https://www.ncbi.nlm.nih.gov/pubmed/25831508) 2015 Apr 7;112(14):4346-51.  **TRAPPII regulates exocytic Golgi exit by mediating nucleotide exchange on the Ypt31 ortholog RabERAB11**.  [Pinar M](https://www.ncbi.nlm.nih.gov/pubmed/?term=Pinar%20M%5BAuthor%5D&cauthor=true&cauthor_uid=25831508)1, [Arst HN Jr](https://www.ncbi.nlm.nih.gov/pubmed/?term=Arst%20HN%20Jr%5BAuthor%5D&cauthor=true&cauthor_uid=25831508)2, [Pantazopoulou A](https://www.ncbi.nlm.nih.gov/pubmed/?term=Pantazopoulou%20A%5BAuthor%5D&cauthor=true&cauthor_uid=25831508)1, [Tagua VG](https://www.ncbi.nlm.nih.gov/pubmed/?term=Tagua%20VG%5BAuthor%5D&cauthor=true&cauthor_uid=25831508)1, [de los Ríos V](https://www.ncbi.nlm.nih.gov/pubmed/?term=de%20los%20R%C3%ADos%20V%5BAuthor%5D&cauthor=true&cauthor_uid=25831508)1, [Rodríguez-Salarichs J](https://www.ncbi.nlm.nih.gov/pubmed/?term=Rodr%C3%ADguez-Salarichs%20J%5BAuthor%5D&cauthor=true&cauthor_uid=25831508)3, [Díaz JF](https://www.ncbi.nlm.nih.gov/pubmed/?term=D%C3%ADaz%20JF%5BAuthor%5D&cauthor=true&cauthor_uid=25831508)3, [Peñalva MA](https://www.ncbi.nlm.nih.gov/pubmed/?term=Pe%C3%B1alva%20MA%5BAuthor%5D&cauthor=true&cauthor_uid=25831508)4.  The oligomeric complex transport protein particle I (TRAPPI) mediates nucleotide exchange on the RAB GTPase RAB1/Ypt1. TRAPPII is composed of TRAPPI plus three additional subunits, Trs120, Trs130, and Trs65. Unclear is whether TRAPPII mediates nucleotide exchange on RAB1/Ypt1, RAB11/Ypt31, or both. In Aspergillus nidulans, RabO(RAB1) resides in the Golgi, RabE(RAB11) localizes to exocytic post-Golgi carriers undergoing transport to the apex, and hypA encodes Trs120. RabE(RAB11), but not RabO(RAB1), immunoprecipitates contain Trs120/Trs130/Trs65, demonstrating specific association of TRAPPII with RabE(RAB11) in vivo. hypA1(ts) rapidly shifts RabE(RAB11), but not RabO(RAB1), to the cytosol, consistent with HypA(Trs120) being specifically required for RabE(RAB11) activation. Missense mutations rescuing hypA1(ts) at 42 °C mapped to rabE, affecting seven residues. Substitutions in six, of which four resulted in 7- to 36-fold accelerated GDP release, rescued lethality associated to TRAPPII deficiency, whereas equivalent substitutions in RabO(RAB1) did not, establishing that the essential role of TRAPPII is facilitating RabE(RAB11) nucleotide exchange. In vitro, TRAPPII purified with HypA(Trs120)-S-tag accelerates nucleotide exchange on RabE(RAB11) and, paradoxically, to a lesser yet substantial extent, on RabO(RAB1). Evidence obtained by exploiting hypA1-mediated destabilization of HypA(Trs120)/HypC(Trs130)/Trs65 assembly onto the TRAPPI core indicates that these subunits sculpt a second RAB binding site on TRAPP apparently independent from that for RabO(RAB1), which would explain TRAPPII in vitro activity on two RABs. Using A. nidulans in vivo microscopy, we show that HypA(Trs120) colocalizes with RabE(RAB11), arriving at late Golgi cisternae as they dissipate into exocytic carriers. Thus, TRAPPII marks, and possibly determines, the Golgi-to-post-Golgi transition. |

| **Gene** | **Spearman** | **Description** | **Name in Scer** | **Description SGD** | **Relevant literature** |
| --- | --- | --- | --- | --- | --- |
| An08g05190  [Genes An08g05180 and An08g05190 were merged (and consequently deleted) to create An08g05185. See Locus History for details.](http://www.aspergillusgenome.org/cgi-bin/locusHistory.pl?dbid=ASPL0000190431)  (hypC) | 0,75 | Ortholog(s) have TRAPPII protein complex localization | TRS130 | Component of transport protein particle (TRAPP) complex II; TRAPPII is a multimeric guanine nucleotide-exchange factor for the GTPase Ypt1p, regulating intra-Golgi and endosome-Golgi traffic | [Proc Natl Acad Sci U S A.](https://www.ncbi.nlm.nih.gov/pubmed/25831508) 2015 Apr 7;112(14):4346-51.  **TRAPPII regulates exocytic Golgi exit by mediating nucleotide exchange on the Ypt31 ortholog RabERAB11**.  [Pinar M](https://www.ncbi.nlm.nih.gov/pubmed/?term=Pinar%20M%5BAuthor%5D&cauthor=true&cauthor_uid=25831508)1, [Arst HN Jr](https://www.ncbi.nlm.nih.gov/pubmed/?term=Arst%20HN%20Jr%5BAuthor%5D&cauthor=true&cauthor_uid=25831508)2, [Pantazopoulou A](https://www.ncbi.nlm.nih.gov/pubmed/?term=Pantazopoulou%20A%5BAuthor%5D&cauthor=true&cauthor_uid=25831508)1, [Tagua VG](https://www.ncbi.nlm.nih.gov/pubmed/?term=Tagua%20VG%5BAuthor%5D&cauthor=true&cauthor_uid=25831508)1, [de los Ríos V](https://www.ncbi.nlm.nih.gov/pubmed/?term=de%20los%20R%C3%ADos%20V%5BAuthor%5D&cauthor=true&cauthor_uid=25831508)1, [Rodríguez-Salarichs J](https://www.ncbi.nlm.nih.gov/pubmed/?term=Rodr%C3%ADguez-Salarichs%20J%5BAuthor%5D&cauthor=true&cauthor_uid=25831508)3, [Díaz JF](https://www.ncbi.nlm.nih.gov/pubmed/?term=D%C3%ADaz%20JF%5BAuthor%5D&cauthor=true&cauthor_uid=25831508)3, [Peñalva MA](https://www.ncbi.nlm.nih.gov/pubmed/?term=Pe%C3%B1alva%20MA%5BAuthor%5D&cauthor=true&cauthor_uid=25831508)4.  The oligomeric complex transport protein particle I (TRAPPI) mediates nucleotide exchange on the RAB GTPase RAB1/Ypt1. TRAPPII is composed of TRAPPI plus three additional subunits, Trs120, Trs130, and Trs65. Unclear is whether TRAPPII mediates nucleotide exchange on RAB1/Ypt1, RAB11/Ypt31, or both. In Aspergillus nidulans, RabO(RAB1) resides in the Golgi, RabE(RAB11) localizes to exocytic post-Golgi carriers undergoing transport to the apex, and hypA encodes Trs120. RabE(RAB11), but not RabO(RAB1), immunoprecipitates contain Trs120/Trs130/Trs65, demonstrating specific association of TRAPPII with RabE(RAB11) in vivo. hypA1(ts) rapidly shifts RabE(RAB11), but not RabO(RAB1), to the cytosol, consistent with HypA(Trs120) being specifically required for RabE(RAB11) activation. Missense mutations rescuing hypA1(ts) at 42 °C mapped to rabE, affecting seven residues. Substitutions in six, of which four resulted in 7- to 36-fold accelerated GDP release, rescued lethality associated to TRAPPII deficiency, whereas equivalent substitutions in RabO(RAB1) did not, establishing that the essential role of TRAPPII is facilitating RabE(RAB11) nucleotide exchange. In vitro, TRAPPII purified with HypA(Trs120)-S-tag accelerates nucleotide exchange on RabE(RAB11) and, paradoxically, to a lesser yet substantial extent, on RabO(RAB1). Evidence obtained by exploiting hypA1-mediated destabilization of HypA(Trs120)/HypC(Trs130)/Trs65 assembly onto the TRAPPI core indicates that these subunits sculpt a second RAB binding site on TRAPP apparently independent from that for RabO(RAB1), which would explain TRAPPII in vitro activity on two RABs. Using A. nidulans in vivo microscopy, we show that HypA(Trs120) colocalizes with RabE(RAB11), arriving at late Golgi cisternae as they dissipate into exocytic carriers. Thus, TRAPPII marks, and possibly determines, the Golgi-to-post-Golgi transition. |
| **Gene** | **Spearman** | **Description** | **Name in Scer** | **Description SGD** | **Relevant literature** |
| An13g00740 | 0,7 | Ortholog(s) have methyltransferase activity, role in filamentous growth, glucosylceramide biosynthetic process, pathogenesis and plasma membrane localization | /  MTS1 (C. albicans) | /  Sphingolipid C9-methyltransferase; catalyzes methylation of the 9th carbon in the long chain base component of glucosylceramides; glucosylceramide biosynthesis is important for virulence; Spider biofilm repressed | [Microbiology.](https://www.ncbi.nlm.nih.gov/pubmed/20019081) 2010 Apr;156(Pt 4):1234-43.  **Candida albicans sphingolipid C9-methyltransferase is involved in hyphal elongation.**  [Oura T](https://www.ncbi.nlm.nih.gov/pubmed/?term=Oura%20T%5BAuthor%5D&cauthor=true&cauthor_uid=20019081)1, [Kajiwara S](https://www.ncbi.nlm.nih.gov/pubmed/?term=Kajiwara%20S%5BAuthor%5D&cauthor=true&cauthor_uid=20019081).  C9-methylated glucosylceramide is a fungus-specific sphingolipid. This lipid is a major membrane component in the cell and is thought to play important roles in the growth and virulence of several fungal species. To investigate the importance of the methyl branch of the long-chain base in glucosylceramides in pathogenic fungi, we identified and characterized a sphingolipid C9-methyltransferase gene (MTS1, C9-MethylTransferase for Sphingolipid 1) in the pathogenic yeast Candida albicans. The mts1 disruptant lacked (E,E)-9-methylsphinga-4,8-dienine in its glucosylceramides and contained (E)-sphing-4-enine and (E,E)-sphinga-4,8-dienine. Reintroducing the MTS1 gene into the mts1 disruptant restored the synthesis of (E,E)-9-methylsphinga-4,8-dienine in the glucosylceramides. We also created a disruptant of the HSX11 gene, encoding glucosylceramide synthase, which catalyses the final step of glucosylceramide synthesis, in C. albicans and compared this mutant with the mts1 disruptant. The C. albicans mts1 and hsx11 disruptants both had a decreased hyphal growth rate compared to the wild-type strain. The hsx11 disruptant showed increased susceptibility to SDS and fluconazole, similar to a previously reported sld1 disruptant that contained only (E)-sphing-4-enine in its glucosylceramides, suggesting that these strains have defects in their cell membrane structures. In contrast, the mts1 disruptant grew similarly to wild-type in medium containing SDS or fluconazole. These results suggest that the C9-methyl group of a long-chain base in glucosylceramides plays an important role in the hyphal elongation of C. albicans independent of lipid membrane disruption. |

| **Gene** | **Spearman** | **Description** | **Name in Scer** | **Description SGD** | **Relevant literature** |
| --- | --- | --- | --- | --- | --- |
| An08g07210  (flbE) | 0,7 | activator functionally associated with FlbB | / | / | [Mol Microbiol.](https://www.ncbi.nlm.nih.gov/pubmed/26256571) 2015 Nov;98(4):607-24.  **Tip-to-nucleus migration dynamics of the asexual development regulator FlbB in vegetative cells.**  [Herrero-Garcia E](https://www.ncbi.nlm.nih.gov/pubmed/?term=Herrero-Garcia%20E%5BAuthor%5D&cauthor=true&cauthor_uid=26256571)1, [Perez-de-Nanclares-Arregi E](https://www.ncbi.nlm.nih.gov/pubmed/?term=Perez-de-Nanclares-Arregi%20E%5BAuthor%5D&cauthor=true&cauthor_uid=26256571)2, [Cortese MS](https://www.ncbi.nlm.nih.gov/pubmed/?term=Cortese%20MS%5BAuthor%5D&cauthor=true&cauthor_uid=26256571)2, [Markina-Iñarrairaegui A](https://www.ncbi.nlm.nih.gov/pubmed/?term=Markina-I%C3%B1arrairaegui%20A%5BAuthor%5D&cauthor=true&cauthor_uid=26256571)2, [Oiartzabal-Arano E](https://www.ncbi.nlm.nih.gov/pubmed/?term=Oiartzabal-Arano%20E%5BAuthor%5D&cauthor=true&cauthor_uid=26256571)2, [Etxebeste O](https://www.ncbi.nlm.nih.gov/pubmed/?term=Etxebeste%20O%5BAuthor%5D&cauthor=true&cauthor_uid=26256571)2, [Ugalde U](https://www.ncbi.nlm.nih.gov/pubmed/?term=Ugalde%20U%5BAuthor%5D&cauthor=true&cauthor_uid=26256571)2, [Espeso EA](https://www.ncbi.nlm.nih.gov/pubmed/?term=Espeso%20EA%5BAuthor%5D&cauthor=true&cauthor_uid=26256571)1.  In Aspergillus nidulans, asexual differentiation requires the presence of the transcription factor FlbB at the cell tip and apical nuclei. Understanding the relationship between these two pools is crucial for elucidating the biochemical processes mediating conidia production. Tip-to-nucleus communication was demonstrated by photo-convertible FlbB::Dendra2 visualization. Tip localization of FlbB depends on Cys382 in the C-terminus and the bZIP DNA-binding domain in the N-terminus. FlbE, a critical FlbB interactor, binds the bZIP domain. Furthermore, the absence of FlbE results in loss of tip localization but not nuclear accumulation. flbE deletion also abrogates transcriptional activity indicating that FlbB gains transcriptional competence from interactions with FlbE at the tip. Finally, a bipartite nuclear localization signal is required for nuclear localization of FlbB. Those motifs of FlbB may play various roles in the sequence of events necessary for the distribution and activation of this transcriptionally active developmental factor. The tip accumulation, FlbE-dependent activation, transport and nuclear import sketch out a process of relaying an environmentally triggered signal from the tip to the nuclei. As the first known instance of transcription factor-mediated tip-to-nucleus communication in filamentous fungi, this provides a general framework for analyses focused on elucidating the set of molecular mechanisms coupling apical signals to transcriptional events. |

| **Gene** | **Spearman** | **Description** | **Name in Scer** | **Description SGD** | **Relevant literature** |
| --- | --- | --- | --- | --- | --- |
| An09g06580 | 0,75 | Has domain(s) with predicted nucleic acid binding, nucleotide binding activity, role in transport and intracellular localization | BRE5 | Ubiquitin protease cofactor; forms deubiquitination complex with Ubp3p that coregulates anterograde and retrograde transport between the endoplasmic reticulum and Golgi compartments; null is sensitive to brefeldin A | [Biochem Biophys Res Commun.](https://www.ncbi.nlm.nih.gov/pubmed/26657849) 2016 Jan 15;469(3):333-9.  **Rck1 promotes pseudohyphal growth via the activation of Ubp3 phosphorylation in Saccharomyces cerevisiae.**  [Kang CM](https://www.ncbi.nlm.nih.gov/pubmed/?term=Kang%20CM%5BAuthor%5D&cauthor=true&cauthor_uid=26657849)1, [Chang M](https://www.ncbi.nlm.nih.gov/pubmed/?term=Chang%20M%5BAuthor%5D&cauthor=true&cauthor_uid=26657849)1, [Park YS](https://www.ncbi.nlm.nih.gov/pubmed/?term=Park%20YS%5BAuthor%5D&cauthor=true&cauthor_uid=26657849)1, [Yun CW](https://www.ncbi.nlm.nih.gov/pubmed/?term=Yun%20CW%5BAuthor%5D&cauthor=true&cauthor_uid=26657849)2.  [Author information](https://www.ncbi.nlm.nih.gov/pubmed/26657849)  Abstract  Previously, we reported that Rck1 up-regulates Ras2 and pseudohyphal growth of Saccharomyces cerevisiae. Here, we further investigate the involvement of Rck1 in the activation of pseudohyphal growth. Rck1 activated phosphorylation of the deubiquitinase Ubp3 through a direct protein interaction between Rck1 and Ubp3. The N-terminal Bre5 binding region of Ubp3 physically interacted with Rck1, and Ubp3 and Rck1 co-precipitated. Overexpression of UBP3 using a high-copy plasmid resulted in the upregulation of Ras2, and deletion of UBP3 blocked the upregulation of Ras2 by RCK1 overexpression. Treatment with the proteasome inhibitor MG132 resulted in accumulation of Ras2, indicating that Rck1 is involved in Ras2 degradation in a proteasome-dependent manner. Furthermore, deletion of UBP3 blocked the upregulation of FLO11, a flocculin required for pseudohyphal and invasive growth induced by RCK1 overexpression in S. cerevisiae. Taken together, these results demonstrate that Rck1 promotes S. cerevisiae pseudohyphal growth via the activation of Ubp3 phosphorylation. |

| **Gene** | **Spearman** | **Description** | **Name in Scer** | **Description SGD** | **Relevant literature** |
| --- | --- | --- | --- | --- | --- |
| An02g08630 | 0,7 | Ortholog(s) have protein serine/threonine kinase activity and role in cellular response to glucose starvation, fungal-type cell wall organization or biogenesis, hyphal growth, response to oxidative stress | PKH2 | Serine/threonine protein kinase; involved in sphingolipid-mediated signaling pathway that controls endocytosis; activates Ypk1p and Ykr2p, components of signaling cascade required for maintenance of cell wall integrity; contains a PH-like domain; redundant with Pkh1p; PKH2 has a paralog, PKH1, that arose from the whole genome duplication | [EMBO J.](https://www.ncbi.nlm.nih.gov/pubmed/11726514) 2001 Dec 3;20(23):6783-92.  **Sphingoid base signaling via Pkh kinases is required for endocytosis in yeast.**  [Friant S](https://www.ncbi.nlm.nih.gov/pubmed/?term=Friant%20S%5BAuthor%5D&cauthor=true&cauthor_uid=11726514)1, [Lombardi R](https://www.ncbi.nlm.nih.gov/pubmed/?term=Lombardi%20R%5BAuthor%5D&cauthor=true&cauthor_uid=11726514), [Schmelzle T](https://www.ncbi.nlm.nih.gov/pubmed/?term=Schmelzle%20T%5BAuthor%5D&cauthor=true&cauthor_uid=11726514), [Hall MN](https://www.ncbi.nlm.nih.gov/pubmed/?term=Hall%20MN%5BAuthor%5D&cauthor=true&cauthor_uid=11726514), [Riezman H](https://www.ncbi.nlm.nih.gov/pubmed/?term=Riezman%20H%5BAuthor%5D&cauthor=true&cauthor_uid=11726514).  In yeast, sphingoid base synthesis is required for the internalization step of endocytosis and organization of the actin cytoskeleton. We show that overexpression of either one of the two kinases Pkh1p or Pkh2p, that are homologous to mammalian 3-phosphoinositide-dependent kinase-1 (PDK1), can specifically suppress the sphingoid base synthesis requirement for endocytosis. Pkh1p and Pkh2p have an overlapping function because only a mutant with impaired function of both kinases is defective for endocytosis. Pkh1/2p kinases are activated in vitro by nanomolar concentrations of sphingoid base. These results suggest that Pkh1/2p kinases are part of a sphingoid base-mediated signaling pathway that is required for the internalization step of endocytosis. The Pkc1p kinase that is phosphorylated by Pkh1/2p kinases and plays a role in endocytosis was identified as one of the downstream effectors of this signaling cascade.  [Mol Biol Cell.](https://www.ncbi.nlm.nih.gov/pubmed/12221112) 2002 Sep;13(9):3005-28.  **Pkh1 and Pkh2 differentially phosphorylate and activate Ypk1 and Ykr2 and define protein kinase modules required for maintenance of cell wall integrity.**  [Roelants FM](https://www.ncbi.nlm.nih.gov/pubmed/?term=Roelants%20FM%5BAuthor%5D&cauthor=true&cauthor_uid=12221112)1, [Torrance PD](https://www.ncbi.nlm.nih.gov/pubmed/?term=Torrance%20PD%5BAuthor%5D&cauthor=true&cauthor_uid=12221112), [Bezman N](https://www.ncbi.nlm.nih.gov/pubmed/?term=Bezman%20N%5BAuthor%5D&cauthor=true&cauthor_uid=12221112), [Thorner J](https://www.ncbi.nlm.nih.gov/pubmed/?term=Thorner%20J%5BAuthor%5D&cauthor=true&cauthor_uid=12221112).  Saccharomyces cerevisiae Pkh1 and Pkh2 are functionally redundant homologs of mammalian protein kinase, phosphoinositide-dependent protein kinase-1. They activate two closely related, functionally redundant enzymes, Ypk1 and Ykr2 (homologs of mammalian protein kinase, serum- and glucocorticoid-inducible protein kinase). We found that Ypk1 has a more prominent role than Ykr2 in mediating their shared essential function. … |

| **Gene** | **Spearman** | **Description** | **Name in Scer** | **Description SGD** | **Relevant literature** |
| --- | --- | --- | --- | --- | --- |
| An01g02600 | 0,75 | Ortholog(s) have role in endosomal transport and AP-1 adaptor complex, endosome localization | APL4 | Gamma-adaptin; large subunit of the clathrin-associated protein (AP-1) complex; binds clathrin; involved in vesicle mediated transport | [Mol Biol Cell.](https://www.ncbi.nlm.nih.gov/pubmed/20739461) 2010 Oct 15;21(20):3552-66. **Alpha-arrestins Aly1 and Aly2 regulate intracellular trafficking in response to nutrient signaling.**  [O'Donnell AF](https://www.ncbi.nlm.nih.gov/pubmed/?term=O'Donnell%20AF%5BAuthor%5D&cauthor=true&cauthor_uid=20739461)1, [Apffel A](https://www.ncbi.nlm.nih.gov/pubmed/?term=Apffel%20A%5BAuthor%5D&cauthor=true&cauthor_uid=20739461), [Gardner RG](https://www.ncbi.nlm.nih.gov/pubmed/?term=Gardner%20RG%5BAuthor%5D&cauthor=true&cauthor_uid=20739461), [Cyert MS](https://www.ncbi.nlm.nih.gov/pubmed/?term=Cyert%20MS%5BAuthor%5D&cauthor=true&cauthor_uid=20739461).  Extracellular signals regulate trafficking events to reorganize proteins at the plasma membrane (PM); however, few effectors of this regulation have been identified. β-Arrestins relay signaling cues to the trafficking machinery by controlling agonist-stimulated endocytosis of G-protein-coupled receptors. In contrast, we show that yeast α-arrestins, Aly1 and Aly2, control intracellular sorting of Gap1, the general amino acid permease, in response to nutrients. These studies are the first to demonstrate association of α-arrestins with clathrin and clathrin adaptor proteins (AP) and show that Aly1 and Aly2 interact directly with the γ-subunit of AP-1, Apl4. Aly2-dependent trafficking of Gap1 requires AP-1, which mediates endosome-to-Golgi transport, and the nutrient-regulated kinase, Npr1, which phosphorylates Aly2. During nitrogen starvation, Npr1 phosphorylation of Aly2 may stimulate Gap1 incorporation into AP-1/clathrin-coated vesicles to promote Gap1 trafficking from endosomes to the trans-Golgi network. Ultimately, increased Aly1-/Aly2-mediated recycling of Gap1 from endosomes results in higher Gap1 levels within cells and at the PM by diverting Gap away from trafficking pathways that lead to vacuolar degradation. This work defines a new role for arrestins in membrane trafficking and offers insight into how α-arrestins coordinate signaling events with protein trafficking. |

| **Gene** | **Spearman** | **Description** | **Name in Scer** | **Description SGD** | **Relevant literature** |
| --- | --- | --- | --- | --- | --- |
| An01g07420 | 0,75 | Ortholog(s) have cyclin-dependent protein serine/threonine kinase activator activity | CLB2 | B-type cyclin involved in cell cycle progression; activates Cdc28p to promote the transition from G2 to M phase; accumulates during G2 and M, then targeted via a destruction box motif for ubiquitin-mediated degradation by the proteasome; CLB2 has a paralog, CLB1, that arose from the whole genome duplication | [Curr Opin Microbiol.](https://www.ncbi.nlm.nih.gov/pubmed/11731325) 2001 Dec;4(6):720-7. **Cell cycle control of yeast filamentous growth.** [Rua D](https://www.ncbi.nlm.nih.gov/pubmed/?term=Rua%20D%5BAuthor%5D&cauthor=true&cauthor_uid=11731325)1, [Tobe BT](https://www.ncbi.nlm.nih.gov/pubmed/?term=Tobe%20BT%5BAuthor%5D&cauthor=true&cauthor_uid=11731325), [Kron SJ](https://www.ncbi.nlm.nih.gov/pubmed/?term=Kron%20SJ%5BAuthor%5D&cauthor=true&cauthor_uid=11731325).  Great progress has been made toward dissecting the signal transduction pathways and transcriptional outputs regulating yeast pseudohyphal growth. However, the mechanism underlying polarized morphogenesis in filamentous growth remains unclear. A synthesis of the data suggests that the ultimate target of these pathways is to repress the activity of the mitotic cyclin Clb2 as an antagonist of polarized growth. Here, we discuss how this cell cycle regulation, in concert with control of transcription, ubiquitin-dependent proteolysis and cytoskeletal polarity, may mediate the switch to filamentous growth. |

| **Gene** | **Spearman** | **Description** | **Name in Scer** | **Description SGD** | **Relevant literature** |
| --- | --- | --- | --- | --- | --- |
| An02g08660  (ugmA) | 0,7 | UDP-galactopyranose mutase, involved in cell wall biosynthesis; induced by growth on starch and lactate | / | / | [Cell Microbiol.](https://www.ncbi.nlm.nih.gov/pubmed/27264789) 2016 Sep;18(9):1268-84.  **Transcriptomic and molecular genetic analysis of the cell wall salvage response of Aspergillus niger to the absence of galactofuranose synthesis.**  [Park J](https://www.ncbi.nlm.nih.gov/pubmed/?term=Park%20J%5BAuthor%5D&cauthor=true&cauthor_uid=27264789)1, [Hulsman M](https://www.ncbi.nlm.nih.gov/pubmed/?term=Hulsman%20M%5BAuthor%5D&cauthor=true&cauthor_uid=27264789)2, [Arentshorst M](https://www.ncbi.nlm.nih.gov/pubmed/?term=Arentshorst%20M%5BAuthor%5D&cauthor=true&cauthor_uid=27264789)1, [Breeman M](https://www.ncbi.nlm.nih.gov/pubmed/?term=Breeman%20M%5BAuthor%5D&cauthor=true&cauthor_uid=27264789)1, [Alazi E](https://www.ncbi.nlm.nih.gov/pubmed/?term=Alazi%20E%5BAuthor%5D&cauthor=true&cauthor_uid=27264789)1, [Lagendijk EL](https://www.ncbi.nlm.nih.gov/pubmed/?term=Lagendijk%20EL%5BAuthor%5D&cauthor=true&cauthor_uid=27264789)1, [Rocha MC](https://www.ncbi.nlm.nih.gov/pubmed/?term=Rocha%20MC%5BAuthor%5D&cauthor=true&cauthor_uid=27264789)3, [Malavazi I](https://www.ncbi.nlm.nih.gov/pubmed/?term=Malavazi%20I%5BAuthor%5D&cauthor=true&cauthor_uid=27264789)3, [Nitsche BM](https://www.ncbi.nlm.nih.gov/pubmed/?term=Nitsche%20BM%5BAuthor%5D&cauthor=true&cauthor_uid=27264789)4, [van den Hondel CA](https://www.ncbi.nlm.nih.gov/pubmed/?term=van%20den%20Hondel%20CA%5BAuthor%5D&cauthor=true&cauthor_uid=27264789)1, [Meyer V](https://www.ncbi.nlm.nih.gov/pubmed/?term=Meyer%20V%5BAuthor%5D&cauthor=true&cauthor_uid=27264789)4, [Ram AF](https://www.ncbi.nlm.nih.gov/pubmed/?term=Ram%20AF%5BAuthor%5D&cauthor=true&cauthor_uid=27264789)1.  The biosynthesis of cell surface-located galactofuranose (Galf)-containing glycostructures such as galactomannan, N-glycans and O-glycans in filamentous fungi is important to secure the integrity of the cell wall. UgmA encodes an UDP-galactopyranose mutase, which is essential for the formation of Galf. Consequently, the ΔugmA mutant lacks Galf-containing molecules. Our previous work in Aspergillus niger work suggested that loss of function of ugmA results in activation of the cell wall integrity (CWI) pathway which is characterized by increased expression of the agsA gene, encoding an α-glucan synthase. In this study, the transcriptional response of the ΔugmA mutant was further linked to the CWI pathway by showing the induced and constitutive phosphorylation of the CWI-MAP kinase in the ΔugmA mutant. To identify genes involved in cell wall remodelling in response to the absence of galactofuranose biosynthesis, a genome-wide expression analysis was performed using RNAseq. Over 400 genes were higher expressed in the ΔugmA mutant compared to the wild-type. These include genes that encode enzymes involved in chitin (gfaB, gnsA, chsA) and α-glucan synthesis (agsA), and in β-glucan remodelling (bgxA, gelF and dfgC), and also include several glycosylphosphatidylinositol (GPI)-anchored cell wall protein-encoding genes. In silico analysis of the 1-kb promoter regions of the up-regulated genes in the ΔugmA mutant indicated overrepresentation of genes with RlmA, MsnA, PacC and SteA-binding sites. The importance of these transcription factors for survival of the ΔugmA mutant was analysed by constructing the respective double mutants. The ΔugmA/ΔrlmA and ΔugmA/ΔmsnA double mutants showed strong synthetic growth defects, indicating the importance of these transcription factors to maintain cell wall integrity in the absence of Galf biosynthesis. |

| **Gene** | **Spearman** | **Description** | **Name in Scer** | **Description SGD** | **Relevant literature** |
| --- | --- | --- | --- | --- | --- |
| An02g09230 | 0,7 | Has domain(s) with predicted phosphatidylinositol N-acetylglucosaminyltransferase activity | GPI15 | Protein involved in the synthesis of GlcNAc-PI; GlcNAc-PI is the first intermediate in the synthesis of glycosylphosphatidylinositol (GPI) anchors; homologous to the human PIG-H protein; GlcNAc-PI stands for N-acetylglucosaminyl phosphatidylinositol | [Yeast.](https://www.ncbi.nlm.nih.gov/pubmed/11746600) 2001 Nov;18(15):1383-9.  **Ynl038wp (Gpi15p) is the Saccharomyces cerevisiae homologue of human Pig-Hp and participates in the first step in glycosylphosphatidylinositol assembly.**  [Yan BC](https://www.ncbi.nlm.nih.gov/pubmed/?term=Yan%20BC%5BAuthor%5D&cauthor=true&cauthor_uid=11746600)1, [Westfall BA](https://www.ncbi.nlm.nih.gov/pubmed/?term=Westfall%20BA%5BAuthor%5D&cauthor=true&cauthor_uid=11746600), [Orlean P](https://www.ncbi.nlm.nih.gov/pubmed/?term=Orlean%20P%5BAuthor%5D&cauthor=true&cauthor_uid=11746600).  Glycosylphosphatidylinositols (GPIs) are found in all eukaryotes and are synthesized in a pathway that starts with the transfer of N-acetylglucosamine (GlcNAc) from UDP-GlcNAc to phosphatidylinositol (PI). This reaction is carried out by a protein complex, three of whose subunits in humans, hGpi1p, Pig-Cp and Pig-Ap, have sequence and functional homologues in the Saccharomyces cerevisiae Gpi1, Gpi2 and Gpi3 proteins, respectively. Human GlcNAc-PI synthase contains two further subunits, Pig-Hp and PigPp. We report that the essential YNL038w gene encodes the S. cerevisiae homologue of Pig-Hp. Haploid YNL038w-deletion strains were created, in which Ynl038wp could be depleted by repressing YNL038w expression using the GAL10 promoter. Depletion of Ynl038wp from membranes virtually abolished in vitro GlcNAc-PI synthetic activity, indicating that Ynl038wp is necessary for GlcNAc-PI synthesis in vitro. Further, depletion of Ynl038wp in an smp3 mutant background prevented the formation of the trimannosylated GPI intermediates that normally accumulate in this late-stage GPI assembly mutant. Ynl038wp is therefore required for GPI synthesis in vivo. Because YNL038w encodes a protein involved in GPI biosynthesis, we designate the gene GPI15. Potential Pig-Hp/Gpi15p counterparts are also encoded in the genomes of Schizosacchomyces pombe and Candida albicans. |

| **Gene** | **Spearman** | **Description** | **Name in Scer** | **Description SGD** | **Relevant literature** |
| --- | --- | --- | --- | --- | --- |
| An05g00280 | 0,75 | Ortholog(s) have protein serine/threonine kinase activity, protein tyrosine kinase activity | SWE1 | Protein kinase that regulates the G2/M transition; negative regulator of the Cdc28p kinase; morphogenesis checkpoint kinase; positive regulator of sphingolipid biosynthesis via Orm2p; phosphorylates a tyrosine residue in the N-terminus of Hsp90 in a cell-cycle associated manner, thus modulating the ability of Hsp90 to chaperone a selected clientele; localizes to the nucleus and to the daughter side of the mother-bud neck; homolog of S. pombe Wee1p; potential Cdc28p substrate | [Sci Rep.](https://www.ncbi.nlm.nih.gov/pubmed/29654251) 2018 Apr 13;8(1):5964. doi: 10.1038/s41598-018-24341-y.  **Deciphering the mechanism of action of 089, a compound impairing the fungal cell cycle.**  [Stefanini I](https://www.ncbi.nlm.nih.gov/pubmed/?term=Stefanini%20I%5BAuthor%5D&cauthor=true&cauthor_uid=29654251)1,2, [Rizzetto L](https://www.ncbi.nlm.nih.gov/pubmed/?term=Rizzetto%20L%5BAuthor%5D&cauthor=true&cauthor_uid=29654251)3, [Rivero D](https://www.ncbi.nlm.nih.gov/pubmed/?term=Rivero%20D%5BAuthor%5D&cauthor=true&cauthor_uid=29654251)4, [Carbonell S](https://www.ncbi.nlm.nih.gov/pubmed/?term=Carbonell%20S%5BAuthor%5D&cauthor=true&cauthor_uid=29654251)5,6, [Gut M](https://www.ncbi.nlm.nih.gov/pubmed/?term=Gut%20M%5BAuthor%5D&cauthor=true&cauthor_uid=29654251)5,6, [Heath S](https://www.ncbi.nlm.nih.gov/pubmed/?term=Heath%20S%5BAuthor%5D&cauthor=true&cauthor_uid=29654251)5,6, [Gut IG](https://www.ncbi.nlm.nih.gov/pubmed/?term=Gut%20IG%5BAuthor%5D&cauthor=true&cauthor_uid=29654251)5,6, [Trabocchi A](https://www.ncbi.nlm.nih.gov/pubmed/?term=Trabocchi%20A%5BAuthor%5D&cauthor=true&cauthor_uid=29654251)7, [Guarna A](https://www.ncbi.nlm.nih.gov/pubmed/?term=Guarna%20A%5BAuthor%5D&cauthor=true&cauthor_uid=29654251)7, [Ben Ghazzi N](https://www.ncbi.nlm.nih.gov/pubmed/?term=Ben%20Ghazzi%20N%5BAuthor%5D&cauthor=true&cauthor_uid=29654251)8,9, [Bowyer P](https://www.ncbi.nlm.nih.gov/pubmed/?term=Bowyer%20P%5BAuthor%5D&cauthor=true&cauthor_uid=29654251)8,9, [Kapushesky M](https://www.ncbi.nlm.nih.gov/pubmed/?term=Kapushesky%20M%5BAuthor%5D&cauthor=true&cauthor_uid=29654251)10, [Cavalieri D](https://www.ncbi.nlm.nih.gov/pubmed/?term=Cavalieri%20D%5BAuthor%5D&cauthor=true&cauthor_uid=29654251)11,12.  Fungal infections represent an increasingly relevant clinical problem, primarily because of the increased survival of severely immune-compromised patients. Despite the availability of active and selective drugs and of well-established prophylaxis, classical antifungals are often ineffective as resistance is frequently observed. The quest for anti-fungal drugs with novel mechanisms of action is thus important. Here we show that a new compound, 089, acts by arresting fungal cells in the G2 phase of the cell cycle through targeting of SWE1, a mechanism of action unexploited by current anti-fungal drugs. The cell cycle impairment also induces a modification of fungal cell morphology which makes fungal cells recognizable by immune cells. This new class of molecules holds promise to be a valuable source of novel antifungals, allowing the clearance of pathogenic fungi by both direct killing of the fungus and enhancing the recognition of the pathogen by the host immune system.  [EMBO Rep.](https://www.ncbi.nlm.nih.gov/pubmed/16485023) 2006 May;7(5):519-24.  **Involvement of calcineurin-dependent degradation of Yap1p in Ca2+-induced G2 cell-cycle regulation in Saccharomyces cerevisiae.**  [Yokoyama H](https://www.ncbi.nlm.nih.gov/pubmed/?term=Yokoyama%20H%5BAuthor%5D&cauthor=true&cauthor_uid=16485023)1, [Mizunuma M](https://www.ncbi.nlm.nih.gov/pubmed/?term=Mizunuma%20M%5BAuthor%5D&cauthor=true&cauthor_uid=16485023), [Okamoto M](https://www.ncbi.nlm.nih.gov/pubmed/?term=Okamoto%20M%5BAuthor%5D&cauthor=true&cauthor_uid=16485023), [Yamamoto J](https://www.ncbi.nlm.nih.gov/pubmed/?term=Yamamoto%20J%5BAuthor%5D&cauthor=true&cauthor_uid=16485023), [Hirata D](https://www.ncbi.nlm.nih.gov/pubmed/?term=Hirata%20D%5BAuthor%5D&cauthor=true&cauthor_uid=16485023), [Miyakawa T](https://www.ncbi.nlm.nih.gov/pubmed/?term=Miyakawa%20T%5BAuthor%5D&cauthor=true&cauthor_uid=16485023).  The Ca2+-activated pathways in Saccharomyces cerevisiae induce a delay in the onset of mitosis through the activation of Swe1p, a negative regulatory kinase that inhibits the Cdc28p/Clb complex. We isolated the YAP1 gene as a multicopy suppressor of calcium sensitivity owing to the loss of ZDS1, a negative regulator of SWE1 and CLN2 gene expression. YAP1 deletion on a zds1delta background exacerbated the Ca2+-related phenotype. Yap1p was degraded in a calcineurin-dependent manner when cells were exposed to calcium. In yap1delta cells, the expression level of the RPN4 gene encoding a transcription factor for the subunits of the ubiquitin-proteasome system was diminished. The deletion of YAP1 gene or RPN4 gene led to the accumulation of Swe1p and Cln2p. Yap1p was a substrate of calcineurin in vivo and in vitro. The calcineurin-mediated Yap1p degradation seems to be a long adaptive response that assures a G2 delay in response to a stress that causes the activation of the calcium signalling pathways. |

| **Gene** | **Spearman** | **Description** | **Name in Scer** | **Description SGD** | **Relevant literature** |
| --- | --- | --- | --- | --- | --- |
| An08g04400 | 0,75 | Ortholog(s) have zinc ion transmembrane transporter activity, role in cellular zinc ion homeostasis, zinc ion transmembrane transport and Golgi membrane localization | zrg17 | Endoplasmic reticulum zinc transporter; part of a heterodimeric transporter with Msc2p that transfers zinc from the cytosol to the ER lumen; member of the cation diffusion facilitator family of efflux pumps; zinc-regulated directly through Zap1p; transcription induced under conditions of zinc deficiency | [Biochem J.](https://www.ncbi.nlm.nih.gov/pubmed/21250939) 2011 Apr 1;435(1):259-66. doi: 10.1042/BJ20102003.  **Transcriptional regulation of the Zrg17 zinc transporter of the yeast secretory pathway.**  [Wu YH](https://www.ncbi.nlm.nih.gov/pubmed/?term=Wu%20YH%5BAuthor%5D&cauthor=true&cauthor_uid=21250939)1, [Frey AG](https://www.ncbi.nlm.nih.gov/pubmed/?term=Frey%20AG%5BAuthor%5D&cauthor=true&cauthor_uid=21250939), [Eide DJ](https://www.ncbi.nlm.nih.gov/pubmed/?term=Eide%20DJ%5BAuthor%5D&cauthor=true&cauthor_uid=21250939).  The Msc2 and Zrg17 proteins of Saccharomyces cerevisiae are members of the cation diffusion facilitator family of zinc transporters. These proteins form heteromeric complexes that transport zinc into the ER (endoplasmic reticulum). Previous studies suggested that the ZRG17 gene is regulated in response to zinc status by the Zap1 transcription factor. Zap1 activates the expression of many genes in zinc-deficient cells. In the present study, we assessed whether ZRG17 is a direct Zap1 target gene. We showed that ZRG17 mRNA levels were elevated in zinc-limited cells in a Zap1-dependent manner and were also elevated in zinc-replete cells expressing a constitutively active allele of Zap1. Furthermore, Zrg17 protein levels correlated closely with mRNA levels. A candidate Zap1-binding site [ZRE (zinc-responsive element)] in the ZRG17 promoter was required for this induction. Using electrophoretic mobility-shift assays and chromatin immunoprecipitation, we demonstrated that Zap1 binds specifically to the ZRG17 ZRE both in vitro and in vivo. By using a chromosomal ZRG17 mutant with a non-functional ZRE, we found that Zap1 induction of ZRG17 is required for ER function as indicated by elevated ER stress under zinc-limited conditions. Together, these results establish that ZRG17 is a direct Zap1 target gene and its regulation has biological importance in maintaining ER function. |

| **Gene** | **Spearman** | **Description** | **Name in Scer** | **Description SGD** | **Relevant literature** |
| --- | --- | --- | --- | --- | --- |
| An15g00910 | 0,75 | Ortholog(s) have AMP-activated protein kinase activity, receptor signaling complex scaffold activity | SIP2 | One of three beta subunits of the Snf1 kinase complex; involved in the response to glucose starvation; null mutants exhibit accelerated aging; N-myristoylprotein localized to the cytoplasm and the plasma membrane; SIP2 has a paralog, GAL83, that arose from the whole genome duplication | [EXS.](https://www.ncbi.nlm.nih.gov/pubmed/27812987) 2016;107:353-374. **AMPK in Yeast: The SNF1 (Sucrose Non-fermenting 1) Protein Kinase Complex.**  [Sanz P](https://www.ncbi.nlm.nih.gov/pubmed/?term=Sanz%20P%5BAuthor%5D&cauthor=true&cauthor_uid=27812987)1, [Viana R](https://www.ncbi.nlm.nih.gov/pubmed/?term=Viana%20R%5BAuthor%5D&cauthor=true&cauthor_uid=27812987)2, [Garcia-Gimeno MA](https://www.ncbi.nlm.nih.gov/pubmed/?term=Garcia-Gimeno%20MA%5BAuthor%5D&cauthor=true&cauthor_uid=27812987)3.  In yeast, SNF1 protein kinase is the orthologue of mammalian AMPK complex. It is a trimeric complex composed of Snf1 protein kinase (orthologue of AMPKα catalytic subunit), Snf4 (orthologue of AMPKγ regulatory subunit), and a member of the Gal83/Sip1/Sip2 family of proteins (orthologues of AMPKβ subunit) that act as scaffolds and also regulate the subcellular localization of the complex. In this chapter, we review the recent literature on the characteristics of SNF1 complex subunits, the structure and regulation of the activity of the SNF1 complex, its role at the level of transcriptional regulation of relevant target genes and also at the level of posttranslational modification of targeted substrates. We also review the crosstalk of SNF1 complex activity with other key protein kinase pathways such as cAMP-PKA, TORC1, and PAS kinase. |

| **Gene** | **Spearman** | **Description** | **Name in Scer** | **Description SGD** | **Relevant literature** |
| --- | --- | --- | --- | --- | --- |
| An01g10200 | 0,75 | Has domain(s) with predicted zinc ion binding activity | CTI6 | Component of the Rpd3L histone deacetylase complex; relieves transcriptional repression by binding to the Cyc8p-Tup1p corepressor and recruiting the SAGA complex to the repressed promoter; contains a PHD finger domain | [J Biol Chem.](https://www.ncbi.nlm.nih.gov/pubmed/23733183) 2013 Jul 12;288(28):20633-45.  **The phosphatidylinositol 3,5-bisphosphate (PI(3,5)P2)-dependent Tup1 conversion (PIPTC) regulates metabolic reprogramming from glycolysis to gluconeogenesis.**  [Han BK](https://www.ncbi.nlm.nih.gov/pubmed/?term=Han%20BK%5BAuthor%5D&cauthor=true&cauthor_uid=23733183)1, [Emr SD](https://www.ncbi.nlm.nih.gov/pubmed/?term=Emr%20SD%5BAuthor%5D&cauthor=true&cauthor_uid=23733183).  Glucose/carbon metabolism is a fundamental cellular process in living cells. In response to varying environments, eukaryotic cells reprogram their glucose/carbon metabolism between aerobic or anaerobic glycolysis, oxidative phosphorylation, and/or gluconeogenesis. The distinct type of glucose/carbon metabolism that a cell carries out has significant effects on the cell's proliferation and differentiation. However, it is poorly understood how the reprogramming of glucose/carbon metabolism is regulated. Here, we report a novel endosomal PI(3,5)P2 lipid-dependent regulatory mechanism that is required for metabolic reprogramming from glycolysis to gluconeogenesis in Saccharomyces cerevisiae. Certain gluconeogenesis genes, such as FBP1 (encoding fructose-1,6-bisphosphatase 1) and ICL1 (encoding isocitrate lyase 1) are under control of the Mig1 repressor and Cyc8-Tup1 corepressor complex. We previously identified the PI(3,5)P2-dependent Tup1 conversion (PIPTC), a mechanism to convert Cyc8-Tup1 corepressor to Cti6-Cyc8-Tup1 coactivator. We demonstrate that the PIPTC plays a critical role for transcriptional activation of FBP1 and ICL1. Furthermore, without the PIPTC, the Cat8 and Sip4 transcriptional activators cannot be efficiently recruited to the promoters of FBP1 and ICL1, suggesting a key role for the PIPTC in remodulating the chromatin architecture at the promoters. Our findings expand our understanding of the regulatory mechanisms for metabolic reprogramming in eukaryotes to include key regulation steps outside the nucleus. Given that Tup1 and the metabolic enzymes that control PI(3,5)P2 are highly conserved among eukaryotes, our findings may provide important insights toward understanding glucose/carbon metabolic reprogramming in other eukaryotes, including humans. |

| **Gene** | **Spearman** | **Description** | **Name in Scer** | **Description SGD** | **Relevant literature** |
| --- | --- | --- | --- | --- | --- |
| An01g13700 | -0,7 | Has domain(s) with predicted nucleic acid binding, zinc ion binding activity | CRZ1 | Transcription factor, activates transcription of stress response genes; nuclear localization is positively regulated by calcineurin-mediated dephosphorylation; rapidly localizes to the nucleus under blue light stress; can be activated in stochastic pulses of nuclear localization in response to calcium | [Fungal Biol Biotechnol.](https://www.ncbi.nlm.nih.gov/pubmed/28955447) 2014 Dec 1;1:5.  **The capacity of Aspergillus niger to sense and respond to cell wall stress requires at least three transcription factors: RlmA, MsnA and CrzA.**  [Fiedler MR](https://www.ncbi.nlm.nih.gov/pubmed/?term=Fiedler%20MR%5BAuthor%5D&cauthor=true&cauthor_uid=28955447)1, [Lorenz A](https://www.ncbi.nlm.nih.gov/pubmed/?term=Lorenz%20A%5BAuthor%5D&cauthor=true&cauthor_uid=28955447)2, [Nitsche BM](https://www.ncbi.nlm.nih.gov/pubmed/?term=Nitsche%20BM%5BAuthor%5D&cauthor=true&cauthor_uid=28955447)1,2, [van den Hondel CA](https://www.ncbi.nlm.nih.gov/pubmed/?term=van%20den%20Hondel%20CA%5BAuthor%5D&cauthor=true&cauthor_uid=28955447)3, [Ram AF](https://www.ncbi.nlm.nih.gov/pubmed/?term=Ram%20AF%5BAuthor%5D&cauthor=true&cauthor_uid=28955447)2,4, [Meyer V](https://www.ncbi.nlm.nih.gov/pubmed/?term=Meyer%20V%5BAuthor%5D&cauthor=true&cauthor_uid=28955447)1,2,4.  Cell wall integrity, vesicle transport and protein secretion are key factors contributing to the vitality and productivity of filamentous fungal cell factories such as Aspergillus niger. In order to pioneer rational strain improvement programs, fundamental knowledge on the genetic basis of these processes is required. The aim of the present study was thus to unravel survival strategies of A. niger when challenged with compounds interfering directly or indirectly with its cell wall integrity: calcofluor white, caspofungin, aureobasidin A, FK506 and fenpropimorph.  Transcriptomics signatures of A. niger and phenotypic analyses of selected null mutant strains were used to predict regulator proteins mediating the survival responses against these stressors. This integrated approach allowed us to reconstruct a model for the cell wall salvage gene network of A. niger that ensures survival of the fungus upon cell surface stress. The model predicts that (i) caspofungin and aureobasidin A induce the cell wall integrity pathway as a main compensatory response via induction of RhoB and RhoD, respectively, eventually activating the mitogen-activated protein kinase kinase MkkA and the transcription factor RlmA. (ii) RlmA is the main transcription factor required for the protection against calcofluor white but it cooperates with MsnA and CrzA to ensure survival of A. niger when challenged with caspofungin and aureobasidin A. (iii) Membrane stress provoked by aureobasidin A via disturbance of sphingolipid synthesis induces cell wall stress, whereas fenpropimorph-induced disturbance of ergosterol synthesis does not.  The present work uncovered a sophisticated defence system of A. niger which employs at least three transcription factors - RlmA, MsnA and CrzA - to protect itself against cell wall stress. The transcriptomic data furthermore predicts a fourth transfactor, SrbA, which seems to be specifically important to survive fenpropimorph-induced cell membrane stress. Future studies will disclose how these regulators are interlocked in different signaling pathways to secure survival of A. niger under different cell wall stress conditions. |
| **Gene** | **Spearman** | **Description** | **Name in Scer** | **Description SGD** | **Relevant literature** |
| An15g02370 | -0,7 | Has domain(s) with predicted transcription corepressor activity | OPI1 | Transcriptional regulator of a variety of genes; phosphorylation by protein kinase A stimulates Opi1p function in negative regulation of phospholipid biosynthetic genes; involved in telomere maintenance; null exhibits disrupted mitochondrial metabolism and low cardiolipin content, strongly correlated with overproduction of inositol; binds to phosphatidic acid | [J Biol Chem.](https://www.ncbi.nlm.nih.gov/pubmed/29127205) 2017 Nov 10;292(45):18729-18730.  **Orchestrating phospholipid biosynthesis: Phosphatidic acid conducts and Opi1p performs.**  [Salsaa M](https://www.ncbi.nlm.nih.gov/pubmed/?term=Salsaa%20M%5BAuthor%5D&cauthor=true&cauthor_uid=29127205)1, [Case K](https://www.ncbi.nlm.nih.gov/pubmed/?term=Case%20K%5BAuthor%5D&cauthor=true&cauthor_uid=29127205)1, [Greenberg ML](https://www.ncbi.nlm.nih.gov/pubmed/?term=Greenberg%20ML%5BAuthor%5D&cauthor=true&cauthor_uid=29127205)2.  Phosphatidic acid (PA) and the conserved integral ER membrane protein Scs2p regulate localization of the transcriptional repressor Opi1p, which controls expression of phospholipid biosynthesis genes, but the mechanisms conducting Opi1p localization are not fully understood. A new study suggests the existence of a distinct pool of PA in the ER that is required for regulation of Opi1p localization and thus phospholipid metabolism in yeast. |

| **Gene** | **Spearman** | **Description** | **Name in Scer** | **Description SGD** | **Relevant literature** |
| --- | --- | --- | --- | --- | --- |
| An12g10750 | 0,75 | Has domain(s) with predicted DNA binding, RNA polymerase II transcription factor activity, sequence-specific DNA binding, zinc ion binding activity and role in regulation of transcription | prt1 (S. pombe) | / | predicted Zn(II)2Cys6 transcription factor, uncharacterized in S. pombe, C. albicans |

| **Gene** | **Spearman** | **Description** | **Name in Scer** | **Description SGD** | **Relevant literature** |
| --- | --- | --- | --- | --- | --- |
| An01g12410 | 0,7 | Has domain(s) with predicted DNA binding, RNA polymerase II transcription factor activity, sequence-specific DNA binding, zinc ion binding activity and role in regulation of transcription, DNA-templated, transcription, DNA-templated | ARO80 | [Zinc finger transcriptional activator of the Zn2Cys6 family; activates transcription of aromatic amino acid catabolic genes in the presence of aromatic amino acids](https://www.yeastgenome.org/locus/S000002829#reference) | [Mol Microbiol.](https://www.ncbi.nlm.nih.gov/pubmed/23651256) 2013 Jun;88(6):1120-34.  **Interplay of Aro80 and GATA activators in regulation of genes for catabolism of aromatic amino acids in Saccharomyces cerevisiae**.  [Lee K](https://www.ncbi.nlm.nih.gov/pubmed/?term=Lee%20K%5BAuthor%5D&cauthor=true&cauthor_uid=23651256)1, [Hahn JS](https://www.ncbi.nlm.nih.gov/pubmed/?term=Hahn%20JS%5BAuthor%5D&cauthor=true&cauthor_uid=23651256).  Aro80, a member of the Zn(2)Cys(6) family proteins, activates expression of the ARO9 and ARO10 genes involved in catabolism of aromatic amino acids in response to aromatic amino acids that act as inducers. ARO9 and ARO10 are also under the control of nitrogen catabolite repression, but the direct roles for GATA factors, Gat1 and Gln3, in this regulation have not yet been elucidated. Here, we demonstrate that Aro80 is constitutively bound to its target promoters and activated by inducers at the level of transactivation. Although Aro80 also binds to its own promoter, ARO80 expression is induced only by rapamycin, but not by tryptophan. We show that Aro80 is absolutely required for Gat1 binding to the ARO9, ARO10 and ARO80 promoters upon rapamycin treatment. Gln3 binding to these promoters shows a partial requirement for Aro80. Rapamycin-dependent Gat1 and Gln3 binding to the Aro80 target promoters is not affected by tryptophan availability, suggesting that transactivation activity of Aro80 is not necessary for the recruitment of GATA factors. Rapamycin-dependent induction of Aro80 target genes also requires PP2A phosphatase complex, but not Sit4 phosphatase, acting downstream of TORC1. |

| **Gene** | **Spearman** | **Description** | **Name in Scer** | **Description SGD** | **Relevant literature** |
| --- | --- | --- | --- | --- | --- |
| An02g08950 | 0,7 | Has domain(s) with predicted DNA binding, zinc ion binding activity | /  HPZ1 (S. pombe) | /  zf-PARP type zinc finger protein, G1-S transition regulator Hpz1 | [PLoS One.](https://www.ncbi.nlm.nih.gov/pubmed/?term=hpz1+pombe) 2012;7(9):e44539.  Hpz1 modulates the G1-S transition in fission yeast.  [Bøe CA](https://www.ncbi.nlm.nih.gov/pubmed/?term=B%C3%B8e%20CA%5BAuthor%5D&cauthor=true&cauthor_uid=22970243)1, [Knutsen JH](https://www.ncbi.nlm.nih.gov/pubmed/?term=Knutsen%20JH%5BAuthor%5D&cauthor=true&cauthor_uid=22970243), [Boye E](https://www.ncbi.nlm.nih.gov/pubmed/?term=Boye%20E%5BAuthor%5D&cauthor=true&cauthor_uid=22970243), [Grallert B](https://www.ncbi.nlm.nih.gov/pubmed/?term=Grallert%20B%5BAuthor%5D&cauthor=true&cauthor_uid=22970243).  [Author information](https://www.ncbi.nlm.nih.gov/pubmed/?term=hpz1+pombe)  Here we characterize a novel protein in S. pombe. It has a high degree of homology with the Zn-finger domain of the human Poly(ADP-ribose) polymerase (PARP). Surprisingly, the gene for this protein is, in many fungi, fused with and in the same reading frame as that encoding Rad3, the homologue of the human ATR checkpoint protein. We name the protein Hpz1 (Homologue of PARP-type Zn-finger). Hpz1 does not possess PARP activity, but is important for resistance to ultraviolet light in the G1 phase and to treatment with hydroxyurea, a drug that arrests DNA replication forks in the S phase. However, we find no evidence of a checkpoint function of Hpz1. Furthermore, absence of Hpz1 results in an advancement of S-phase entry after a G1 arrest as well as earlier recovery from a hydroxyurea block. The hpz1 gene is expressed mainly in the G1 phase and Hpz1 is localized to the nucleus. We conclude that Hpz1 regulates the initiation of the S phase and may cooperate with Rad3 in this function. |

| **Gene** | **Spearman** | **Description** | **Name in Scer** | **Description SGD** | **Relevant literature** |
| --- | --- | --- | --- | --- | --- |
| An03g01480 | 0,7 | Has domain(s) with predicted DNA binding, nucleic acid binding, zinc ion binding activity, role in transcription, DNA-templated and nucleus localization | SDD4 | Putative transcription factor  SDD: Suppressor of Degenerative Death | [Nature.](https://www.ncbi.nlm.nih.gov/pubmed/26192197) 2015 Aug 27;524(7566):481-4.  **A cytosolic network suppressing mitochondria-mediated proteostatic stress and cell death.**  [Wang X](https://www.ncbi.nlm.nih.gov/pubmed/?term=Wang%20X%5BAuthor%5D&cauthor=true&cauthor_uid=26192197)1, [Chen XJ](https://www.ncbi.nlm.nih.gov/pubmed/?term=Chen%20XJ%5BAuthor%5D&cauthor=true&cauthor_uid=26192197)1.  Mitochondria are multifunctional organelles whose dysfunction leads to neuromuscular degeneration and ageing. The multi-functionality poses a great challenge for understanding the mechanisms by which mitochondrial dysfunction causes specific pathologies. Among the leading mitochondrial mediators of cell death are energy depletion, free radical production, defects in iron-sulfur cluster biosynthesis, the release of pro-apoptotic and non-cell-autonomous signalling molecules, and altered stress signalling. Here we identify a new pathway of mitochondria-mediated cell death in yeast. This pathway was named mitochondrial precursor over-accumulation stress (mPOS), and is characterized by aberrant accumulation of mitochondrial precursors in the cytosol. mPOS can be triggered by clinically relevant mitochondrial damage that is not limited to the core machineries of protein import. We also discover a large network of genes that suppress mPOS, by modulating ribosomal biogenesis, messenger RNA decapping, transcript-specific translation, protein chaperoning and turnover. In response to mPOS, several ribosome-associated proteins were upregulated, including Gis2 and Nog2, which promote cap-independent translation and inhibit the nuclear export of the 60S ribosomal subunit, respectively. Gis2 and Nog2 upregulation promotes cell survival, which may be part of a feedback loop that attenuates mPOS. Our data indicate that mitochondrial dysfunction contributes directly to cytosolic proteostatic stress, and provide an explanation for the association between these two hallmarks of degenerative diseases and ageing. The results are relevant to understanding diseases (for example, spinocerebellar ataxia, amyotrophic lateral sclerosis and myotonic dystrophy) that involve mutations within the anti-degenerative network. |
| **Gene** | **Spearman** | **Description** | **Name in Scer** | **Description SGD** | **Relevant literature** |
| An08g02110 | 0,7 | Has domain(s) with predicted nucleic acid binding activity | USV1 | Putative transcription factor containing a C2H2 zinc finger; mutation affects transcriptional regulation of genes involved in growth on non-fermentable carbon sources, response to salt stress and cell wall biosynthesis; USV1 has a paralog, RGM1, that arose from the whole genome duplication | [Appl Microbiol Biotechnol.](https://www.ncbi.nlm.nih.gov/pubmed/?term=usv1+cerevisiae) 2013 Sep;97(18):8227-38..  **Mutations of the TATA-binding protein confer enhanced tolerance to hyperosmotic stress in Saccharomyces cerevisiae.**  [Kim NR](https://www.ncbi.nlm.nih.gov/pubmed/?term=Kim%20NR%5BAuthor%5D&cauthor=true&cauthor_uid=23709042)1, [Yang J](https://www.ncbi.nlm.nih.gov/pubmed/?term=Yang%20J%5BAuthor%5D&cauthor=true&cauthor_uid=23709042), [Kwon H](https://www.ncbi.nlm.nih.gov/pubmed/?term=Kwon%20H%5BAuthor%5D&cauthor=true&cauthor_uid=23709042), [An J](https://www.ncbi.nlm.nih.gov/pubmed/?term=An%20J%5BAuthor%5D&cauthor=true&cauthor_uid=23709042), [Choi W](https://www.ncbi.nlm.nih.gov/pubmed/?term=Choi%20W%5BAuthor%5D&cauthor=true&cauthor_uid=23709042), [Kim W](https://www.ncbi.nlm.nih.gov/pubmed/?term=Kim%20W%5BAuthor%5D&cauthor=true&cauthor_uid=23709042).  Previously, it was shown that overexpression of either of two SPT15 mutant alleles, SPT15-M2 and SPT15-M3, which encode mutant TATA-binding proteins, confer enhanced ethanol tolerance in Saccharomyces cerevisiae. In this study, we demonstrated that strains overexpressing SPT15-M2 or SPT15-M3 were tolerant to hyperosmotic stress caused by high concentrations of glucose, salt, and sorbitol. The enhanced tolerance to high glucose concentrations in particular improved ethanol production from very high gravity (VHG) ethanol fermentations. The strains displayed constitutive and sustained activation of Hog1, a central kinase in the high osmolarity glycerol (HOG) signal transduction pathway of S. cerevisiae. However, the cell growth defect known to be caused by constitutive and sustained activation of Hog1 was not observed. We also found that reactive oxygen species (ROS) were accumulated to a less extent upon exposure to high glucose concentration in our osmotolerant strains. We identified six new genes (GPH1, HSP12, AIM17, SSA4, USV1, and IGD1), the individual deletion of which renders cells sensitive to 50 % glucose. In spite of the presence of multiple copies of stress response element in their promoters, it was apparent that those genes were not controlled at the transcriptional level by the HOG pathway under the high glucose conditions. Combined with previously published results, overexpression of SPT15-M2 or SPT15-M3 clearly provides a basis for improved tolerance to ethanol and osmotic stress, which enables construction of strains of any genetic background that need enhanced tolerance to high concentrations of ethanol and glucose, promoting the feasibility for VHG ethanol fermentation. |

| **Gene** | **Spearman** | **Description** | **Name in Scer** | **Description SGD** | **Relevant literature** |
| --- | --- | --- | --- | --- | --- |
| An11g09470 | -0,75 | Has domain(s) with predicted DNA binding, chromatin binding activity | Teb1 (S. pombe) | / | [EMBO J.](https://www.ncbi.nlm.nih.gov/pubmed/23314747) 2013 Feb 6;32(3):450-60.  Myb-domain protein Teb1 controls histone levels and centromere assembly in fission yeast.  [Valente LP](https://www.ncbi.nlm.nih.gov/pubmed/?term=Valente%20LP%5BAuthor%5D&cauthor=true&cauthor_uid=23314747)1, [Dehé PM](https://www.ncbi.nlm.nih.gov/pubmed/?term=Deh%C3%A9%20PM%5BAuthor%5D&cauthor=true&cauthor_uid=23314747), [Klutstein M](https://www.ncbi.nlm.nih.gov/pubmed/?term=Klutstein%20M%5BAuthor%5D&cauthor=true&cauthor_uid=23314747), [Aligianni S](https://www.ncbi.nlm.nih.gov/pubmed/?term=Aligianni%20S%5BAuthor%5D&cauthor=true&cauthor_uid=23314747), [Watt S](https://www.ncbi.nlm.nih.gov/pubmed/?term=Watt%20S%5BAuthor%5D&cauthor=true&cauthor_uid=23314747), [Bähler J](https://www.ncbi.nlm.nih.gov/pubmed/?term=B%C3%A4hler%20J%5BAuthor%5D&cauthor=true&cauthor_uid=23314747), [Cooper JP](https://www.ncbi.nlm.nih.gov/pubmed/?term=Cooper%20JP%5BAuthor%5D&cauthor=true&cauthor_uid=23314747).  The TTAGGG motif is common to two seemingly unrelated dimensions of chromatin function-the vertebrate telomere repeat and the promoter regions of many Schizosaccharomyces pombe genes, including all of those encoding canonical histones. The essential S. pombe protein Teb1 contains two Myb-like DNA binding domains related to those found in telomere proteins and binds the human telomere repeat sequence TTAGGG. Here, we analyse Teb1 binding throughout the genome and the consequences of reduced Teb1 function. Chromatin immunoprecipitation (ChIP)-on-chip analysis reveals robust Teb1 binding at many promoters, notably including all of those controlling canonical histone gene expression. A hypomorphic allele, teb1-1, confers reduced binding and reduced levels of histone transcripts. Prompted by previously suggested connections between histone expression and centromere identity, we examined localization of the centromeric histone H3 variant Cnp1 and found reduced centromeric binding along with reduced centromeric silencing. These data identify Teb1 as a master regulator of histone levels and centromere identity. |

| **Gene** | **Spearman** | **Description** | **Name in Scer** | **Description SGD** | **Relevant literature** |
| --- | --- | --- | --- | --- | --- |
| An08g02200 | 0,7 | Ortholog(s) have nucleosome-dependent ATPase activity | CHD1 | Chromatin remodeler that regulates various aspects of transcription; acts in in conjunction with Isw1b to regulate chromatin structure and maintain chromatin integrity during transcription elongation by RNAP II by preventing trans-histone exchange over coding regions; contains a chromo domain, a helicase domain and a DNA-binding domain; component of both the SAGA and SLIK complexes | [Nature.](https://www.ncbi.nlm.nih.gov/pubmed/15647753) 2005 Jan 27;433(7024):434-8.  **Chd1 chromodomain links histone H3 methylation with SAGA- and SLIK-dependent acetylation**.  [Pray-Grant MG](https://www.ncbi.nlm.nih.gov/pubmed/?term=Pray-Grant%20MG%5BAuthor%5D&cauthor=true&cauthor_uid=15647753)1, [Daniel JA](https://www.ncbi.nlm.nih.gov/pubmed/?term=Daniel%20JA%5BAuthor%5D&cauthor=true&cauthor_uid=15647753), [Schieltz D](https://www.ncbi.nlm.nih.gov/pubmed/?term=Schieltz%20D%5BAuthor%5D&cauthor=true&cauthor_uid=15647753), [Yates JR 3rd](https://www.ncbi.nlm.nih.gov/pubmed/?term=Yates%20JR%203rd%5BAuthor%5D&cauthor=true&cauthor_uid=15647753), [Grant PA](https://www.ncbi.nlm.nih.gov/pubmed/?term=Grant%20PA%5BAuthor%5D&cauthor=true&cauthor_uid=15647753).  The specific post-translational modifications to histones influence many nuclear processes including gene regulation, DNA repair and replication. Recent studies have identified effector proteins that recognize patterns of histone modification and transduce their function in downstream processes. For example, histone acetyltransferases (HATs) have been shown to participate in many essential cellular processes, particularly those associated with activation of transcription. Yeast SAGA (Spt-Ada-Gcn5 acetyltransferase) and SLIK (SAGA-like) are two highly homologous and conserved multi-subunit HAT complexes, which preferentially acetylate histones H3 and H2B and deubiquitinate histone H2B. Here we identify the chromatin remodelling protein Chd1 (chromo-ATPase/helicase-DNA binding domain 1) as a component of SAGA and SLIK. Our findings indicate that one of the two chromodomains of Chd1 specifically interacts with the methylated lysine 4 mark on histone H3 that is associated with transcriptional activity. Furthermore, the SLIK complex shows enhanced acetylation of a methylated substrate and this activity is dependent upon a functional methyl-binding chromodomain, both in vitro and in vivo. Our study identifies the first chromodomain that recognizes methylated histone H3 (Lys 4) and possibly identifies a larger subfamily of chromodomain proteins with similar recognition properties. |

| **Gene** | **Spearman** | **Description** | **Name in Scer** | **Description SGD** | **Relevant literature** |
| --- | --- | --- | --- | --- | --- |
| An08g01090 | -0,75 | Protein with similarity to Ada Histone acetyltransferase complex component Ahc1p of S. cerevisiae | AHC1 | Subunit of the Ada histone acetyltransferase complex; required for structural integrity of the complex; Ahc2p and Ahc1p are unique to the ADA complex and not shared with the related SAGA and SLIK complexes; Ahc2p may tether Ahc1p to the complex | [Mol Cell Biol.](https://www.ncbi.nlm.nih.gov/pubmed/10490601) 1999 Oct;19(10):6621-31.  **The ADA complex is a distinct histone acetyltransferase complex in Saccharomyces cerevisiae.**  [Eberharter A](https://www.ncbi.nlm.nih.gov/pubmed/?term=Eberharter%20A%5BAuthor%5D&cauthor=true&cauthor_uid=10490601)1, [Sterner DE](https://www.ncbi.nlm.nih.gov/pubmed/?term=Sterner%20DE%5BAuthor%5D&cauthor=true&cauthor_uid=10490601), [Schieltz D](https://www.ncbi.nlm.nih.gov/pubmed/?term=Schieltz%20D%5BAuthor%5D&cauthor=true&cauthor_uid=10490601), [Hassan A](https://www.ncbi.nlm.nih.gov/pubmed/?term=Hassan%20A%5BAuthor%5D&cauthor=true&cauthor_uid=10490601), [Yates JR 3rd](https://www.ncbi.nlm.nih.gov/pubmed/?term=Yates%20JR%203rd%5BAuthor%5D&cauthor=true&cauthor_uid=10490601), [Berger SL](https://www.ncbi.nlm.nih.gov/pubmed/?term=Berger%20SL%5BAuthor%5D&cauthor=true&cauthor_uid=10490601), [Workman JL](https://www.ncbi.nlm.nih.gov/pubmed/?term=Workman%20JL%5BAuthor%5D&cauthor=true&cauthor_uid=10490601).  We have identified two Gcn5-dependent histone acetyltransferase (HAT) complexes from Saccharomyces cerevisiae, the 0.8-MDa ADA complex and the 1.8-MDa SAGA complex. The SAGA (Spt-Ada-Gcn5-acetyltransferase) complex contains several subunits which also function as part of other protein complexes, including a subset of TATA box binding protein-associated factors (TAFIIs) and Tra1. These observations raise the question of whether the 0.8-MDa ADA complex is a subcomplex of SAGA or whether it is a distinct HAT complex that also shares subunits with SAGA. To address this issue, we sought to determine if the ADA complex contained subunits that are not present in the SAGA complex. In this study, we report the purification of the ADA complex over 10 chromatographic steps. By a combination of mass spectrometry analysis and immunoblotting, we demonstrate that the adapter proteins Ada2, Ada3, and Gcn5 are indeed integral components of ADA. Furthermore, we identify the product of the S. cerevisiae gene YOR023C as a novel subunit of the ADA complex and name it Ahc1 for ADA HAT complex component 1. Biochemical functions of YOR023C have not been reported. However, AHC1 in high copy numbers suppresses the cold sensitivity caused by particular mutations in HTA1 (I. Pinto and F. Winston, personal communication), which encodes histone H2A (J. N. Hirschhorn et al., Mol. Cell. Biol. 15:1999-2009, 1995). Deletion of AHC1 disrupted the integrity of the ADA complex but did not affect SAGA or give rise to classic Ada(-) phenotypes. These results indicate that Gcn5, Ada2, and Ada3 function as part of a unique HAT complex (ADA) and represent shared subunits between this complex and SAGA. |

| **Gene** | **Spearman** | **Description** | **Name in Scer** | **Description SGD** | **Relevant literature** |
| --- | --- | --- | --- | --- | --- |
| An08g04960, An08g04970  systematic  name:  An08g04965  (merged) | 0,7 | Ortholog(s) have histone demethylase activity (H3-K36 specific) activity and role in histone demethylation, regulation of transcription by RNA polymerase II | rph1 | JmjC domain-containing histone demethylase; targets tri- and dimethylated H3K36; associates with actively transcribed regions and promotes elongation; repressor of autophagy-related genes in nutrient-replete conditions; damage-responsive repressor of PHR1; phosphorylated by the Rad53p-dependent DNA damage checkpoint pathway and by a Rim1p-mediated event during starvation; target of stress-induced hormesis; RPH1 has a paralog, GIS1, that arose from the whole genome duplication | [Genes Dev.](https://www.ncbi.nlm.nih.gov/pubmed/26159996) 2015 Jul 1;29(13):1362-76.  **H3K36 methylation promotes longevity by enhancing transcriptional fidelity.**  [Sen P](https://www.ncbi.nlm.nih.gov/pubmed/?term=Sen%20P%5BAuthor%5D&cauthor=true&cauthor_uid=26159996)1, [Dang W](https://www.ncbi.nlm.nih.gov/pubmed/?term=Dang%20W%5BAuthor%5D&cauthor=true&cauthor_uid=26159996)2, [Donahue G](https://www.ncbi.nlm.nih.gov/pubmed/?term=Donahue%20G%5BAuthor%5D&cauthor=true&cauthor_uid=26159996)1, [Dai J](https://www.ncbi.nlm.nih.gov/pubmed/?term=Dai%20J%5BAuthor%5D&cauthor=true&cauthor_uid=26159996)3, [Dorsey J](https://www.ncbi.nlm.nih.gov/pubmed/?term=Dorsey%20J%5BAuthor%5D&cauthor=true&cauthor_uid=26159996)1, [Cao X](https://www.ncbi.nlm.nih.gov/pubmed/?term=Cao%20X%5BAuthor%5D&cauthor=true&cauthor_uid=26159996)4, [Liu W](https://www.ncbi.nlm.nih.gov/pubmed/?term=Liu%20W%5BAuthor%5D&cauthor=true&cauthor_uid=26159996)3, [Cao K](https://www.ncbi.nlm.nih.gov/pubmed/?term=Cao%20K%5BAuthor%5D&cauthor=true&cauthor_uid=26159996)1, [Perry R](https://www.ncbi.nlm.nih.gov/pubmed/?term=Perry%20R%5BAuthor%5D&cauthor=true&cauthor_uid=26159996)1, [Lee JY](https://www.ncbi.nlm.nih.gov/pubmed/?term=Lee%20JY%5BAuthor%5D&cauthor=true&cauthor_uid=26159996)5, [Wasko BM](https://www.ncbi.nlm.nih.gov/pubmed/?term=Wasko%20BM%5BAuthor%5D&cauthor=true&cauthor_uid=26159996)6, [Carr DT](https://www.ncbi.nlm.nih.gov/pubmed/?term=Carr%20DT%5BAuthor%5D&cauthor=true&cauthor_uid=26159996)6, [He C](https://www.ncbi.nlm.nih.gov/pubmed/?term=He%20C%5BAuthor%5D&cauthor=true&cauthor_uid=26159996)7, [Robison B](https://www.ncbi.nlm.nih.gov/pubmed/?term=Robison%20B%5BAuthor%5D&cauthor=true&cauthor_uid=26159996)7, [Wagner J](https://www.ncbi.nlm.nih.gov/pubmed/?term=Wagner%20J%5BAuthor%5D&cauthor=true&cauthor_uid=26159996)5, [Gregory BD](https://www.ncbi.nlm.nih.gov/pubmed/?term=Gregory%20BD%5BAuthor%5D&cauthor=true&cauthor_uid=26159996)5, [Kaeberlein M](https://www.ncbi.nlm.nih.gov/pubmed/?term=Kaeberlein%20M%5BAuthor%5D&cauthor=true&cauthor_uid=26159996)6, [Kennedy BK](https://www.ncbi.nlm.nih.gov/pubmed/?term=Kennedy%20BK%5BAuthor%5D&cauthor=true&cauthor_uid=26159996)7, [Boeke JD](https://www.ncbi.nlm.nih.gov/pubmed/?term=Boeke%20JD%5BAuthor%5D&cauthor=true&cauthor_uid=26159996)8, [Berger SL](https://www.ncbi.nlm.nih.gov/pubmed/?term=Berger%20SL%5BAuthor%5D&cauthor=true&cauthor_uid=26159996)1.  Epigenetic mechanisms, including histone post-translational modifications, control longevity in diverse organisms. Relatedly, loss of proper transcriptional regulation on a global scale is an emerging phenomenon of shortened life span, but the specific mechanisms linking these observations remain to be uncovered. Here, we describe a life span screen in Saccharomyces cerevisiae that is designed to identify amino acid residues of histones that regulate yeast replicative aging. Our results reveal that lack of sustained histone H3K36 methylation is commensurate with increased cryptic transcription in a subset of genes in old cells and with shorter life span. In contrast, deletion of the K36me2/3 demethylase Rph1 increases H3K36me3 within these genes, suppresses cryptic transcript initiation, and extends life span. We show that this aging phenomenon is conserved, as cryptic transcription also increases in old worms. We propose that epigenetic misregulation in aging cells leads to loss of transcriptional precision that is detrimental to life span, and, importantly, this acceleration in aging can be reversed by restoring transcriptional fidelity. |

| **Gene** | **Spearman** | **Description** | **Name in Scer** | **Description SGD** | **Relevant literature** |
| --- | --- | --- | --- | --- | --- |
| An08g02810 | 0,7 | Ortholog(s) have DNA replication origin binding activity, role in mitotic DNA replication, mitotic DNA replication checkpoint and nuclear chromatin, nuclear origin of replication recognition complex localization | ORC1  Origin Recognition Complex | Largest subunit of the origin recognition complex; involved in directing DNA replication by binding to replication origins; also involved in transcriptional silencing; exhibits ATPase activity; ORC1 has a paralog, SIR3, that arose from the whole genome duplication | [Genome Res.](https://www.ncbi.nlm.nih.gov/pubmed/27895110) 2017 Feb;27(2):269-277.  **Nucleosome occupancy as a novel chromatin parameter for replication origin functions.**  [Rodriguez J](https://www.ncbi.nlm.nih.gov/pubmed/?term=Rodriguez%20J%5BAuthor%5D&cauthor=true&cauthor_uid=27895110)1, [Lee L](https://www.ncbi.nlm.nih.gov/pubmed/?term=Lee%20L%5BAuthor%5D&cauthor=true&cauthor_uid=27895110)1,2, [Lynch B](https://www.ncbi.nlm.nih.gov/pubmed/?term=Lynch%20B%5BAuthor%5D&cauthor=true&cauthor_uid=27895110)1, [Tsukiyama T](https://www.ncbi.nlm.nih.gov/pubmed/?term=Tsukiyama%20T%5BAuthor%5D&cauthor=true&cauthor_uid=27895110)1.  Eukaryotic DNA replication initiates from multiple discrete sites in the genome, termed origins of replication (origins). Prior to S phase, multiple origins are poised to initiate replication by recruitment of the pre-replicative complex (pre-RC). For proper replication to occur, origin activation must be tightly regulated. At the population level, each origin has a distinct firing time and frequency of activation within S phase. Many studies have shown that chromatin can strongly influence initiation of DNA replication. However, the chromatin parameters that affect properties of origins have not been thoroughly established. We found that nucleosome occupancy in G1 varies greatly around origins across the S. cerevisiae genome, and nucleosome occupancy around origins significantly correlates with the activation time and efficiency of origins, as well as pre-RC formation. We further demonstrate that nucleosome occupancy around origins in G1 is established during transition from G2/M to G1 in a pre-RC-dependent manner. Importantly, the diminished cell-cycle changes in nucleosome occupancy around origins in the orc1-161 mutant are associated with an abnormal global origin usage profile, suggesting that proper establishment of nucleosome occupancy around origins is a critical step for regulation of global origin activities. Our work thus establishes nucleosome occupancy as a novel and key chromatin parameter for proper origin regulation. |

| **Gene** | **Spearman** | **Description** | **Name in Scer** | **Description SGD** | **Relevant literature** |
| --- | --- | --- | --- | --- | --- |
| An11g02670 | 0,7 | Ortholog(s) have RSC-type complex localization | RSC9 | Component of the RSC chromatin remodeling complex; DNA-binding protein involved in the synthesis of rRNA and in transcriptional repression and activation of genes regulated by the Target of Rapamycin (TOR) pathway | [Mol Cell.](https://www.ncbi.nlm.nih.gov/pubmed/11931764) 2002 Mar;9(3):563-73.  **The genome-wide localization of Rsc9, a component of the RSC chromatin-remodeling complex, changes in response to stress.**  [Damelin M](https://www.ncbi.nlm.nih.gov/pubmed/?term=Damelin%20M%5BAuthor%5D&cauthor=true&cauthor_uid=11931764)1, [Simon I](https://www.ncbi.nlm.nih.gov/pubmed/?term=Simon%20I%5BAuthor%5D&cauthor=true&cauthor_uid=11931764), [Moy TI](https://www.ncbi.nlm.nih.gov/pubmed/?term=Moy%20TI%5BAuthor%5D&cauthor=true&cauthor_uid=11931764), [Wilson B](https://www.ncbi.nlm.nih.gov/pubmed/?term=Wilson%20B%5BAuthor%5D&cauthor=true&cauthor_uid=11931764), [Komili S](https://www.ncbi.nlm.nih.gov/pubmed/?term=Komili%20S%5BAuthor%5D&cauthor=true&cauthor_uid=11931764), [Tempst P](https://www.ncbi.nlm.nih.gov/pubmed/?term=Tempst%20P%5BAuthor%5D&cauthor=true&cauthor_uid=11931764), [Roth FP](https://www.ncbi.nlm.nih.gov/pubmed/?term=Roth%20FP%5BAuthor%5D&cauthor=true&cauthor_uid=11931764), [Young RA](https://www.ncbi.nlm.nih.gov/pubmed/?term=Young%20RA%5BAuthor%5D&cauthor=true&cauthor_uid=11931764), [Cairns BR](https://www.ncbi.nlm.nih.gov/pubmed/?term=Cairns%20BR%5BAuthor%5D&cauthor=true&cauthor_uid=11931764), [Silver PA](https://www.ncbi.nlm.nih.gov/pubmed/?term=Silver%20PA%5BAuthor%5D&cauthor=true&cauthor_uid=11931764).  The cellular response to environmental changes includes widespread modifications in gene expression. Here we report the identification and characterization of Rsc9, a member of the RSC chromatin-remodeling complex in yeast. The genome-wide localization of Rsc9 indicated a relationship between genes targeted by Rsc9 and genes regulated by stress; treatment with hydrogen peroxide or rapamycin, which inhibits TOR signaling, resulted in genome-wide changes in Rsc9 occupancy. We further show that Rsc9 is involved in both repression and activation of mRNAs regulated by TOR as well as the synthesis of rRNA. Our results illustrate the response of a chromatin-remodeling factor to signaling cascades and suggest that changes in the activity of chromatin-remodeling factors are reflected in changes in their localization in the genome. |

| **Gene** | **Spearman** | **Description** | **Name in Scer** | **Description SGD** | **Relevant literature** |
| --- | --- | --- | --- | --- | --- |
| An16g01520 | 0,8 | Ortholog(s) have role in ascospore formation, conidium formation, hyphal growth, regulation of ascospore formation, regulation of meiosis I and regulation of mitotic nuclear division, more | RSA4 | WD-repeat protein involved in ribosome biogenesis; may interact with ribosomes; required for maturation and efficient intra-nuclear transport or pre-60S ribosomal subunits, localizes to the nucleolus | [Nature.](https://www.ncbi.nlm.nih.gov/pubmed/24240281) 2014 Jan 2;505(7481):112-116. **Coupled GTPase and remodelling ATPase activities form a checkpoint for ribosome export.**  [Matsuo Y](https://www.ncbi.nlm.nih.gov/pubmed/?term=Matsuo%20Y%5BAuthor%5D&cauthor=true&cauthor_uid=24240281)1, [Granneman S](https://www.ncbi.nlm.nih.gov/pubmed/?term=Granneman%20S%5BAuthor%5D&cauthor=true&cauthor_uid=24240281)#2,3, [Thoms M](https://www.ncbi.nlm.nih.gov/pubmed/?term=Thoms%20M%5BAuthor%5D&cauthor=true&cauthor_uid=24240281)#1, [Manikas RG](https://www.ncbi.nlm.nih.gov/pubmed/?term=Manikas%20RG%5BAuthor%5D&cauthor=true&cauthor_uid=24240281)1, [Tollervey D](https://www.ncbi.nlm.nih.gov/pubmed/?term=Tollervey%20D%5BAuthor%5D&cauthor=true&cauthor_uid=24240281)2, [Hurt E](https://www.ncbi.nlm.nih.gov/pubmed/?term=Hurt%20E%5BAuthor%5D&cauthor=true&cauthor_uid=24240281)1.  Eukaryotic ribosomes are assembled by a complex pathway that extends from the nucleolus to the cytoplasm and is powered by many energy-consuming enzymes. Nuclear export is a key, irreversible step in pre-ribosome maturation, but mechanisms underlying the timely acquisition of export competence remain poorly understood. Here we show that a conserved Saccharomyces cerevisiae GTPase Nug2 (also known as Nog2, and as NGP-1, GNL2 or nucleostemin 2 in human) has a key role in the timing of export competence. Nug2 binds the inter-subunit face of maturing, nucleoplasmic pre-60S particles, and the location clashes with the position of Nmd3, a key pre-60S export adaptor. Nug2 and Nmd3 are not present on the same pre-60S particles, with Nug2 binding before Nmd3. Depletion of Nug2 causes premature Nmd3 binding to the pre-60S particles, whereas mutations in the G-domain of Nug2 block Nmd3 recruitment, resulting in severe 60S export defects. Two pre-60S remodelling factors, the Rea1 ATPase and its co-substrate Rsa4, are present on Nug2-associated particles, and both show synthetic lethal interactions with nug2 mutants. Release of Nug2 from pre-60S particles requires both its K(+)-dependent GTPase activity and the remodelling ATPase activity of Rea1. We conclude that Nug2 is a regulatory GTPase that monitors pre-60S maturation, with release from its placeholder site linked to recruitment of the nuclear export machinery. |

| **Gene** | **Spearman** | **Description** | **Name in Scer** | **Description SGD** | **Relevant literature** |
| --- | --- | --- | --- | --- | --- |
| An07g03760 | 0,75 | Has domain(s) with predicted nucleic acid binding activity | Snd1 (H.sapiens) | snd1 of H. sapiens is as coactivator involved in gene transcription probably by interacting with TFIIE and other myb transcription factors.; Phenotype: H. sapiens snd1 overexpression specifically augmented EBNA 2 acidic domain-mediated activation of gene transcription.; Phenotype: snd1 of H. sapiens is essential for normal cell growth, since cell viability was reduced by antisense p100 RNA and restored by sense p100 RNA expression.; Title: strong similarity to 100 kDa coactivator snd1 - Homo sapiens | [Oncotarget.](https://www.ncbi.nlm.nih.gov/pubmed/29296233) 2017 Nov 21;8(64):108181-108194.  **SREBP-2-driven transcriptional activation of human SND1 oncogene.**  [Armengol S](https://www.ncbi.nlm.nih.gov/pubmed/?term=Armengol%20S%5BAuthor%5D&cauthor=true&cauthor_uid=29296233)1, [Arretxe E](https://www.ncbi.nlm.nih.gov/pubmed/?term=Arretxe%20E%5BAuthor%5D&cauthor=true&cauthor_uid=29296233)1, [Enzunza L](https://www.ncbi.nlm.nih.gov/pubmed/?term=Enzunza%20L%5BAuthor%5D&cauthor=true&cauthor_uid=29296233)1, [Llorente I](https://www.ncbi.nlm.nih.gov/pubmed/?term=Llorente%20I%5BAuthor%5D&cauthor=true&cauthor_uid=29296233)1, [Mendibil U](https://www.ncbi.nlm.nih.gov/pubmed/?term=Mendibil%20U%5BAuthor%5D&cauthor=true&cauthor_uid=29296233)1, [Navarro-Imaz H](https://www.ncbi.nlm.nih.gov/pubmed/?term=Navarro-Imaz%20H%5BAuthor%5D&cauthor=true&cauthor_uid=29296233)1, [Ochoa B](https://www.ncbi.nlm.nih.gov/pubmed/?term=Ochoa%20B%5BAuthor%5D&cauthor=true&cauthor_uid=29296233)1, [Chico Y](https://www.ncbi.nlm.nih.gov/pubmed/?term=Chico%20Y%5BAuthor%5D&cauthor=true&cauthor_uid=29296233)1, [Martínez MJ](https://www.ncbi.nlm.nih.gov/pubmed/?term=Mart%C3%ADnez%20MJ%5BAuthor%5D&cauthor=true&cauthor_uid=29296233)1.  Upregulation of Staphylococcal nuclease and tudor domain containing 1 (SND1) is linked to cancer progression and metastatic spread. Increasing evidence indicates that SND1 plays a role in lipid homeostasis. Recently, it has been shown that SND1-overexpressing hepatocellular carcinoma cells present an increased de novo cholesterol synthesis and cholesteryl ester accumulation. Here we reveal that SND1 oncogene is a novel target for SREBPs. Exposure of HepG2 cells to the cholesterol-lowering drug simvastatin or to a lipoprotein-deficient medium triggers SREBP-2 activation and increases SND1 promoter activity and transcript levels. Similar increases in SND1 promoter activity and mRNA are mimicked by overexpressing nuclear SREBP-2 through expression vector transfection. Conversely, SREBP-2 suppression with specific siRNA or the addition of cholesterol/25-hydroxycholesterol to cell culture medium reduces transcriptional activity of SND1 promoter and SND1 mRNA abundance. Chromatin immunoprecipitation assays and site-directed mutagenesis show that SREBP-2 binds to the SND1 proximal promoter in a region containing one SRE and one E-box motif which are critical for maximal transcriptional activity under basal conditions. SREBP-1, in contrast, binds exclusively to the SRE element. Remarkably, while ectopic expression of SREBP-1c or -1a reduces SND1 promoter activity, knocking-down of SREBP-1 enhances SND1 mRNA and protein levels but failed to affect SND1 promoter activity. These findings reveal that SREBP-2 and SREBP-1 bind to specific sites in SND1 promoter and regulate SND1 transcription in opposite ways; it is induced by SREBP-2 activating conditions and repressed by SREBP-1 overexpression. We anticipate the contribution of a SREBPs/SND1 pathway to lipid metabolism reprogramming of human hepatoma cells. |

| **Gene** | **Spearman** | **Description** | **Name in Scer** | **Description SGD** | **Relevant literature** |
| --- | --- | --- | --- | --- | --- |
| An07g08430 | 0,75 | Has domain(s) with predicted role in protein targeting to mitochondrion and mitochondrial outer membrane localization  closest homolog in N. crassa: tob37 | An07g08430 | / | AspGD: note = unnamed protein product; Complex: a yeast two-hybrid screen with metaxin 1 as bait has identified a novel protein, termed metaxin 2, as a metaxin 1-binding protein.; Function: in cell culture overexpression of human metaxin resulted in impaired mitochondrial import of natural and chimeric preproteins and in their accumulation.; Function: metaxin is an outer membrane protein of mammalian mitochondria which is suggested to be involved in protein import into the organelle.; Remark: ORF 3'truncated due to end of contig.; Title: strong similarity to mitochondrial preprotein import complex subunit metaxin  [PLoS One.](https://www.ncbi.nlm.nih.gov/pubmed/21980517) 2011;6(9):e25650. **The Neurospora crassa TOB complex: analysis of the topology and function of Tob38 and Tob37.**  [Lackey SW](https://www.ncbi.nlm.nih.gov/pubmed/?term=Lackey%20SW%5BAuthor%5D&cauthor=true&cauthor_uid=21980517)1, [Wideman JG](https://www.ncbi.nlm.nih.gov/pubmed/?term=Wideman%20JG%5BAuthor%5D&cauthor=true&cauthor_uid=21980517), [Kennedy EK](https://www.ncbi.nlm.nih.gov/pubmed/?term=Kennedy%20EK%5BAuthor%5D&cauthor=true&cauthor_uid=21980517), [Go NE](https://www.ncbi.nlm.nih.gov/pubmed/?term=Go%20NE%5BAuthor%5D&cauthor=true&cauthor_uid=21980517), [Nargang FE](https://www.ncbi.nlm.nih.gov/pubmed/?term=Nargang%20FE%5BAuthor%5D&cauthor=true&cauthor_uid=21980517).  The TOB or SAM complex is responsible for assembling several proteins into the mitochondrial outer membrane, including all β-barrel proteins. We have identified several forms of the complex in Neurospora crassa. One form contains Tob55, Tob38, and Tob37; another contains these three subunits plus the Mdm10 protein; while additional complexes contain only Tob55. As previously shown for Tob55, both Tob37 and Tob38 are essential for viability of the organism. Mitochondria deficient in Tob37 or Tob38 have reduced ability to assemble β-barrel proteins. The function of two hydrophobic domains in the C-terminal region of the Tob37 protein was investigated. Mutant Tob37 proteins lacking either or both of these regions are able to restore viability to cells lacking the protein. One of the domains was found to anchor the protein to the outer mitochondrial membrane but was not necessary for targeting or association of the protein with mitochondria. Examination of the import properties of mitochondria containing Tob37 with deletions of the hydrophobic domains reveals that the topology of Tob37 may be important for interactions between specific classes of β-barrel precursors and the TOB complex. |

| **Gene** | **Spearman** | **Description** | **Name in Scer** | **Description SGD** | **Relevant literature** |
| --- | --- | --- | --- | --- | --- |
| An01g14720 | 0,75 | Has domain(s) with predicted DNA binding, zinc ion binding activity, role in transcription, DNA-templated and nucleus localization | - | / | ortholog of zas1 (S.pombe)  [J Cell Biol.](https://www.ncbi.nlm.nih.gov/pubmed/?term=zas1++pombe) 2018 May 7. pii: jcb.201711097.  **Control of mitotic chromosome condensation by the fission yeast transcription factor Zas1.**  [Schiklenk C](https://www.ncbi.nlm.nih.gov/pubmed/?term=Schiklenk%20C%5BAuthor%5D&cauthor=true&cauthor_uid=29735745)1, [Petrova B](https://www.ncbi.nlm.nih.gov/pubmed/?term=Petrova%20B%5BAuthor%5D&cauthor=true&cauthor_uid=29735745)1, [Kschonsak M](https://www.ncbi.nlm.nih.gov/pubmed/?term=Kschonsak%20M%5BAuthor%5D&cauthor=true&cauthor_uid=29735745)1, [Hassler M](https://www.ncbi.nlm.nih.gov/pubmed/?term=Hassler%20M%5BAuthor%5D&cauthor=true&cauthor_uid=29735745)1, [Klein C](https://www.ncbi.nlm.nih.gov/pubmed/?term=Klein%20C%5BAuthor%5D&cauthor=true&cauthor_uid=29735745)1, [Gibson TJ](https://www.ncbi.nlm.nih.gov/pubmed/?term=Gibson%20TJ%5BAuthor%5D&cauthor=true&cauthor_uid=29735745)2, [Haering CH](https://www.ncbi.nlm.nih.gov/pubmed/?term=Haering%20CH%5BAuthor%5D&cauthor=true&cauthor_uid=29735745)3,2.  Although the formation of rod-shaped chromosomes is vital for the correct segregation of eukaryotic genomes during cell divisions, the molecular mechanisms that control the chromosome condensation process have remained largely unknown. Here, we identify the C2H2 zinc-finger transcription factor Zas1 as a key regulator of mitotic condensation dynamics in a quantitative live-cell microscopy screen of the fission yeast Schizosaccharomyces pombe By binding to specific DNA target sequences in their promoter regions, Zas1 controls expression of the Cnd1 subunit of the condensin protein complex and several other target genes, whose combined misregulation in zas1 mutants results in defects in chromosome condensation and segregation. Genetic and biochemical analysis reveals an evolutionarily conserved transactivation domain motif in Zas1 that is pivotal to its function in gene regulation. Our results suggest that this motif, together with the Zas1 C-terminal helical domain to which it binds, creates a cis/trans switch module for transcriptional regulation of genes that control chromosome condensation. |

| **Gene** | **Spearman** | **Description** | **Name in Scer** | **Description SGD** | **Relevant literature** |
| --- | --- | --- | --- | --- | --- |
| An16g08540 | 0,75 | Ortholog(s) have microtubule plus-end binding activity and role in dynein-driven meiotic oscillatory nuclear movement, syncytium formation by plasma membrane fusion | / | [/](https://www.yeastgenome.org/locus/S000004340#reference) | ortholog of meiotic dynein intermediate chain Dic1 in S. pombe  [Genes Cells.](https://www.ncbi.nlm.nih.gov/pubmed/?term=DIC1+pombe) 2010 Apr 1;15(4):359-72.  **Contribution of dynein light intermediate and intermediate chains to subcellular localization of the dynein-dynactin motor complex in Schizosaccharomyces pombe**.  [Fujita I](https://www.ncbi.nlm.nih.gov/pubmed/?term=Fujita%20I%5BAuthor%5D&cauthor=true&cauthor_uid=20298435)1, [Yamashita A](https://www.ncbi.nlm.nih.gov/pubmed/?term=Yamashita%20A%5BAuthor%5D&cauthor=true&cauthor_uid=20298435), [Yamamoto M](https://www.ncbi.nlm.nih.gov/pubmed/?term=Yamamoto%20M%5BAuthor%5D&cauthor=true&cauthor_uid=20298435).  In fission yeast Schizosaccharomyces pombe, cytoplasmic dynein drives oscillatory nuclear movement during meiotic prophase, which may facilitate pairing of homologous chromosomes. Here, we report the identification of a dynein light intermediate chain (LIC) in fission yeast, termed Dli1p, and show that Dli1p and dynein intermediate chain (IC) Dic1p are essential for the appropriate subcellular localization and proper function of dynein during meiotic prophase. Expression of both the dli1 and dic1 genes was observed only in cells undergoing meiosis. Dli1p interacted and colocalized with dynein heavy chain Dhc1p. The subcellular localization of Dli1p was dependent on Dhc1p, and vice versa. The Dhc1p-Dli1p subcomplex could localize to the spindle pole body (SPB) with no aid of Dic1p and dynactin subunit Ssm4p, but its localization to microtubules was dependent on these two proteins. Dic1p localized to microtubules depending on Ssm4p, but not on Dhc1p and Dli1p. Its localization to the SPB, however, was dependent on Dhc1p and Dli1p. Localization of Ssm4p to the SPB was largely dependent on Dhc1p, Dli1p and Dic1p. Thus, Dli1p and Dic1p contribute differently in localizing the dynein-dynactin motor complex to organelles, providing novel insight into the in vivo function of dynein subunits in fission yeast. |

| **Gene** | **Spearman** | **Description** | **Name in Scer** | **Description SGD** | **Relevant literature** |
| --- | --- | --- | --- | --- | --- |
| An14g05920 | 0,75 | Has domain(s) with predicted ATP binding, microtubule binding, microtubule motor activity, role in microtubule-based movement and microtubule associated complex localization | KIP3 | Kinesin-related antiparallel sliding motor protein; involved in mitotic spindle positioning; sliding activity promotes bipolar spindle assembly and maintenance of genome stability; inhibits spindle elongation, destabilizing late anaphase spindle microtubules that polymerize beyond the midzone | [Curr Biol.](https://www.ncbi.nlm.nih.gov/pubmed/25088560) 2014 Aug 18;24(16):1826-35.  **Spatial control of microtubule length and lifetime by opposing stabilizing and destabilizing functions of Kinesin-8.**  [Fukuda Y](https://www.ncbi.nlm.nih.gov/pubmed/?term=Fukuda%20Y%5BAuthor%5D&cauthor=true&cauthor_uid=25088560)1, [Luchniak A](https://www.ncbi.nlm.nih.gov/pubmed/?term=Luchniak%20A%5BAuthor%5D&cauthor=true&cauthor_uid=25088560)2, [Murphy ER](https://www.ncbi.nlm.nih.gov/pubmed/?term=Murphy%20ER%5BAuthor%5D&cauthor=true&cauthor_uid=25088560)1, [Gupta ML Jr](https://www.ncbi.nlm.nih.gov/pubmed/?term=Gupta%20ML%20Jr%5BAuthor%5D&cauthor=true&cauthor_uid=25088560)3.  BACKGROUND:  To function in diverse cellular processes, the dynamic behavior of microtubules (MTs) must be differentially regulated within the cell. In budding yeast, the spindle position checkpoint (SPOC) inhibits mitotic exit in response to mispositioned spindles. To maintain SPOC-mediated anaphase arrest, astral MTs must maintain persistent interactions with and/or extend through the bud neck. However, the molecular mechanisms that ensure the stability of these interactions are not known.  RESULTS:  The presence of an MT extending through and/or interacting with the bud neck is maintained by spatial control of catastrophe and rescue, which extends MT lifetime >25-fold and controls the length of dynamic MTs within the bud compartment. Moreover, the single kinesin-8 motor Kip3 alternately mediates both catastrophe and rescue of the bud MT. Kip3 accumulates in a length-dependent manner along the lattice of MTs within the bud, yet induces catastrophe spatially near the bud tip. Rather, this accumulation of Kip3 facilitates its association with depolymerizing MT plus ends, where Kip3 promotes rescue before MTs exit the bud. MT rescue within the bud requires the tail domain of Kip3, whereas the motor domain mediates catastrophe at the bud tip. In vitro, Kip3 exerts both stabilizing and destabilizing effects on reconstituted yeast MTs.  CONCLUSIONS:  The kinesin-8 Kip3 is a multifunctional regulator that differentially stabilizes and destabilizes specific MTs. Control over MT catastrophe and rescue by Kip3 defines the length and lifetime of MTs within the bud compartment of cells with mispositioned spindles. This subcellular regulation of MT dynamics is critical to maintaining mitotic arrest in response to mispositioned spindles. |

| **Gene** | **Spearman** | **Description** | **Name in Scer** | **Description SGD** | **Relevant literature** |
| --- | --- | --- | --- | --- | --- |
| An02g07690 | 0,7 | Ortholog(s) have ATPase activator activity, cytoskeletal adaptor activity, microtubule plus-end binding, protein homodimerization activity, structural constituent of cytoskeleton activity | BIM1 | Microtubule plus end-tracking protein; together with Kar9p makes up the cortical microtubule capture site and delays the exit from mitosis when the spindle is oriented abnormally; homolog of human end binding protein 1 (EB1) | [Mol Syst Biol.](https://www.ncbi.nlm.nih.gov/pubmed/29618636) 2018 Apr 4;14(4):e7390.  **Single-cell profiling screen identifies microtubule-dependent reduction of variability in signaling.**  [Pesce CG](https://www.ncbi.nlm.nih.gov/pubmed/?term=Pesce%20CG%5BAuthor%5D&cauthor=true&cauthor_uid=29618636)1, [Zdraljevic S](https://www.ncbi.nlm.nih.gov/pubmed/?term=Zdraljevic%20S%5BAuthor%5D&cauthor=true&cauthor_uid=29618636)2, [Peria WJ](https://www.ncbi.nlm.nih.gov/pubmed/?term=Peria%20WJ%5BAuthor%5D&cauthor=true&cauthor_uid=29618636)3, [Bush A](https://www.ncbi.nlm.nih.gov/pubmed/?term=Bush%20A%5BAuthor%5D&cauthor=true&cauthor_uid=29618636)4, [Repetto MV](https://www.ncbi.nlm.nih.gov/pubmed/?term=Repetto%20MV%5BAuthor%5D&cauthor=true&cauthor_uid=29618636)4, [Rockwell D](https://www.ncbi.nlm.nih.gov/pubmed/?term=Rockwell%20D%5BAuthor%5D&cauthor=true&cauthor_uid=29618636)1, [Yu RC](https://www.ncbi.nlm.nih.gov/pubmed/?term=Yu%20RC%5BAuthor%5D&cauthor=true&cauthor_uid=29618636)1, [Colman-Lerner A](https://www.ncbi.nlm.nih.gov/pubmed/?term=Colman-Lerner%20A%5BAuthor%5D&cauthor=true&cauthor_uid=29618636)4, [Brent R](https://www.ncbi.nlm.nih.gov/pubmed/?term=Brent%20R%5BAuthor%5D&cauthor=true&cauthor_uid=29618636)5.  Populations of isogenic cells often respond coherently to signals, despite differences in protein abundance and cell state. Previously, we uncovered processes in the Saccharomyces cerevisiae pheromone response system (PRS) that reduced cell-to-cell variability in signal strength and cellular response. Here, we screened 1,141 non-essential genes to identify 50 "variability genes". Most had distinct, separable effects on strength and variability of the PRS, defining these quantities as genetically distinct "axes" of system behavior. Three genes affected cytoplasmic microtubule function: BIM1, GIM2, and GIM4 We used genetic and chemical perturbations to show that, without microtubules, PRS output is reduced but variability is unaffected, while, when microtubules are present but their function is perturbed, output is sometimes lowered, but its variability is always high. The increased variability caused by microtubule perturbations required the PRS MAP kinase Fus3 and a process at or upstream of Ste5, the membrane-localized scaffold to which Fus3 must bind to be activated. Visualization of Ste5 localization dynamics demonstrated that perturbing microtubules destabilized Ste5 at the membrane signaling site. The fact that such microtubule perturbations cause aberrant fate and polarity decisions in mammals suggests that microtubule-dependent signal stabilization might also operate throughout metazoans. |

| **Gene** | **Spearman** | **Description** | **Name in Scer** | **Description SGD** | **Relevant literature** |
| --- | --- | --- | --- | --- | --- |
| An18g02650 | 0,75 | Has domain(s) with predicted role in transmembrane transport and integral component of membrane localization | VBA5 | Plasma membrane protein of the Major Facilitator Superfamily (MFS); involved in amino acid uptake and drug sensitivity; VBA5 has a paralog, VBA3, that arose from a segmental duplication | [Biosci Biotechnol Biochem.](https://www.ncbi.nlm.nih.gov/pubmed/23047103) 2012;76(10):1993-5. Epub 2012 Oct 7.  **Vba5p, a novel plasma membrane protein involved in amino acid uptake and drug sensitivity in Saccharomyces cerevisiae.**  [Shimazu M](https://www.ncbi.nlm.nih.gov/pubmed/?term=Shimazu%20M%5BAuthor%5D&cauthor=true&cauthor_uid=23047103)1, [Itaya T](https://www.ncbi.nlm.nih.gov/pubmed/?term=Itaya%20T%5BAuthor%5D&cauthor=true&cauthor_uid=23047103), [Pongcharoen P](https://www.ncbi.nlm.nih.gov/pubmed/?term=Pongcharoen%20P%5BAuthor%5D&cauthor=true&cauthor_uid=23047103), [Sekito T](https://www.ncbi.nlm.nih.gov/pubmed/?term=Sekito%20T%5BAuthor%5D&cauthor=true&cauthor_uid=23047103), [Kawano-Kawada M](https://www.ncbi.nlm.nih.gov/pubmed/?term=Kawano-Kawada%20M%5BAuthor%5D&cauthor=true&cauthor_uid=23047103), [Kakinuma Y](https://www.ncbi.nlm.nih.gov/pubmed/?term=Kakinuma%20Y%5BAuthor%5D&cauthor=true&cauthor_uid=23047103).  Vba5p is closest to Vba3p in the vacuolar transporter for basic amino acids (VBA) family of Saccharomyces cerevisiae. We found that green fluorescence protein (GFP)-tagged Vba5p localized exclusively to the plasma membrane. The uptake of lysine and arginine by whole cells was little affected by deletion of the VBA5 gene, but was stimulated by overexpression of the VBA5 gene. The inhibitory effect of 4-nitroquinoline N-oxide on cell growth was accelerated by expression of the VBA5 gene, and was lessened by the addition of arginine. These results suggest that Vba5p is a plasma membrane protein involved in amino acid uptake and drug sensitivity. |

| **Gene** | **Spearman** | **Description** | **Name in Scer** | **Description SGD** | **Relevant literature** |
| --- | --- | --- | --- | --- | --- |
| An03g04340 | 0,7 | Protein with a predicted role in ER SRP-dependent and -independent protein targeting; SEC61 complex subunit; expression enhanced by maltose | SEC61 | Conserved ER protein translocation channel; essential subunit of Sec61 complex (Sec61p, Sbh1p, and Sss1p); forms channel for SRP-dependent protein import; with Sec63 complex allows SRP-independent protein import into ER; involved in posttranslational soluble protein import into the ER, ERAD of soluble substrates, and misfolded soluble protein export from the ER | [PLoS One.](https://www.ncbi.nlm.nih.gov/pubmed/25658429) 2015 Feb 6;10(2):e0117260.  **Proteasome 19S RP binding to the Sec61 channel plays a key role in ERAD.**  [Kaiser ML](https://www.ncbi.nlm.nih.gov/pubmed/?term=Kaiser%20ML%5BAuthor%5D&cauthor=true&cauthor_uid=25658429)1, [Römisch K](https://www.ncbi.nlm.nih.gov/pubmed/?term=R%C3%B6misch%20K%5BAuthor%5D&cauthor=true&cauthor_uid=25658429)1.  Import of secretory proteins into the Endoplasmic Reticulum (ER) is an established function of the Sec61 channel. The contribution of the Sec61 channel to export of misfolded proteins from the ER for degradation by proteasomes is still controversial, but the proteasome 19S regulatory particle (RP) is necessary and sufficient for extraction of specific misfolded proteins from the ER, and binds directly to the Sec61 channel. In this work we have identified an import-competent sec61 mutant, S353C, carrying a point mutation in ER-lumenal loop 7 which reduces affinity of the cytoplasmic face of the Sec61 channel for the 19S RP. This indicates that the interaction between the 19S RP and the Sec61 channel is dependent on conformational changes in Sec61p hinging on loop 7. The sec61-S353C mutant had no measurable ER import defects and did not cause ER stress in intact cells, but reduced ER-export of a 19S RP-dependent misfolded protein when proteasomes were limiting in a cell-free assay. Our data suggest that the interaction between the 19S RP and the Sec61 channel is essential for the export of specific substrates from the ER to the cytosol for proteasomal degradation. |

| **Gene** | **Spearman** | **Description** | **Name in Scer** | **Description SGD** | **Relevant literature** |
| --- | --- | --- | --- | --- | --- |
| An08g02230 | 0,7 | Has domain(s) with predicted role in intra-Golgi vesicle-mediated transport and TRAPPII protein complex localization | / | / |  |

| **Gene** | **Spearman** | **Description** | **Name in Scer** | **Description SGD** | **Relevant literature** |
| --- | --- | --- | --- | --- | --- |
| An02g09470 | 0,7 | Potassium- or calcium-transporting ATPase | SPF1 | P-type ATPase, ion transporter of the ER membrane; required to maintain normal lipid composition of intracellular compartments and proper targeting of mitochondrial outer membrane tail-anchored proteins; involved in ER function and Ca2+ homeostasis; required for regulating Hmg2p degradation | [PLoS One.](https://www.ncbi.nlm.nih.gov/pubmed/24392018) 2013 Dec 31;8(12):e85519.  **The yeast p5 type ATPase, spf1, regulates manganese transport into the endoplasmic reticulum.**  [Cohen Y](https://www.ncbi.nlm.nih.gov/pubmed/?term=Cohen%20Y%5BAuthor%5D&cauthor=true&cauthor_uid=24392018)1, [Megyeri M](https://www.ncbi.nlm.nih.gov/pubmed/?term=Megyeri%20M%5BAuthor%5D&cauthor=true&cauthor_uid=24392018)2, [Chen OC](https://www.ncbi.nlm.nih.gov/pubmed/?term=Chen%20OC%5BAuthor%5D&cauthor=true&cauthor_uid=24392018)3, [Condomitti G](https://www.ncbi.nlm.nih.gov/pubmed/?term=Condomitti%20G%5BAuthor%5D&cauthor=true&cauthor_uid=24392018)4, [Riezman I](https://www.ncbi.nlm.nih.gov/pubmed/?term=Riezman%20I%5BAuthor%5D&cauthor=true&cauthor_uid=24392018)5, [Loizides-Mangold U](https://www.ncbi.nlm.nih.gov/pubmed/?term=Loizides-Mangold%20U%5BAuthor%5D&cauthor=true&cauthor_uid=24392018)5, [Abdul-Sada A](https://www.ncbi.nlm.nih.gov/pubmed/?term=Abdul-Sada%20A%5BAuthor%5D&cauthor=true&cauthor_uid=24392018)6, [Rimon N](https://www.ncbi.nlm.nih.gov/pubmed/?term=Rimon%20N%5BAuthor%5D&cauthor=true&cauthor_uid=24392018)1, [Riezman H](https://www.ncbi.nlm.nih.gov/pubmed/?term=Riezman%20H%5BAuthor%5D&cauthor=true&cauthor_uid=24392018)7, [Platt FM](https://www.ncbi.nlm.nih.gov/pubmed/?term=Platt%20FM%5BAuthor%5D&cauthor=true&cauthor_uid=24392018)3, [Futerman AH](https://www.ncbi.nlm.nih.gov/pubmed/?term=Futerman%20AH%5BAuthor%5D&cauthor=true&cauthor_uid=24392018)8, [Schuldiner M](https://www.ncbi.nlm.nih.gov/pubmed/?term=Schuldiner%20M%5BAuthor%5D&cauthor=true&cauthor_uid=24392018)1.  The endoplasmic reticulum (ER) is a large, multifunctional and essential organelle. Despite intense research, the function of more than a third of ER proteins remains unknown even in the well-studied model organism Saccharomyces cerevisiae. One such protein is Spf1, which is a highly conserved, ER localized, putative P-type ATPase. Deletion of SPF1 causes a wide variety of phenotypes including severe ER stress suggesting that this protein is essential for the normal function of the ER. The closest homologue of Spf1 is the vacuolar P-type ATPase Ypk9 that influences Mn(2+) homeostasis. However in vitro reconstitution assays with Spf1 have not yielded insight into its transport specificity. Here we took an in vivo approach to detect the direct and indirect effects of deleting SPF1. We found a specific reduction in the luminal concentration of Mn(2+) in ∆spf1 cells and an increase following it's overexpression. In agreement with the observed loss of luminal Mn(2+) we could observe concurrent reduction in many Mn(2+)-related process in the ER lumen. Conversely, cytosolic Mn(2+)-dependent processes were increased. Together, these data support a role for Spf1p in Mn(2+) transport in the cell. We also demonstrate that the human sequence homologue, ATP13A1, is a functionally conserved orthologue. Since ATP13A1 is highly expressed in developing neuronal tissues and in the brain, this should help in the study of Mn(2+)-dependent neurological disorders. |

| **Gene** | **Spearman** | **Description** | **Name in Scer** | **Description SGD** | **Relevant literature** |
| --- | --- | --- | --- | --- | --- |
| An04g03600 | 0,7 | Has domain(s) with predicted DNA binding activity | / | / |  |

| **Gene** | **Spearman** | **Description** | **Name in Scer** | **Description SGD** | **Relevant literature** |
| --- | --- | --- | --- | --- | --- |
| An07g01060 | 0,7 | Has domain(s) with predicted role in cell wall macromolecule catabolic process | / | /  AspGD:  unnamed protein product; Remark: possible functions for the CIH1 protein in the establishment and maintenance of biotrophy are discussed.; Title: similarity to CIH1 gene for intracellular hyphae protein 1 - Colletotrichum lindemuthianum | [Mol Plant Pathol.](https://www.ncbi.nlm.nih.gov/pubmed/20572968) 2000 Jul 1;1(4):213-21.  **The distribution and expression of a biotrophy-related gene, CIH1, within the genus Colletotrichum.**  [Perfect SE](https://www.ncbi.nlm.nih.gov/pubmed/?term=Perfect%20SE%5BAuthor%5D&cauthor=true&cauthor_uid=20572968)1, [Pixton KL](https://www.ncbi.nlm.nih.gov/pubmed/?term=Pixton%20KL%5BAuthor%5D&cauthor=true&cauthor_uid=20572968), [O'Connell RJ](https://www.ncbi.nlm.nih.gov/pubmed/?term=O'Connell%20RJ%5BAuthor%5D&cauthor=true&cauthor_uid=20572968), [Green JR](https://www.ncbi.nlm.nih.gov/pubmed/?term=Green%20JR%5BAuthor%5D&cauthor=true&cauthor_uid=20572968).  During the biotrophic phase of the infection process of the hemibiotrophic anthracnose fungus Colletotrichum lindemuthianum, an intracellular hypha develops within epidermal cells of its host, Phaseolus vulgaris. This is followed by the formation of secondary hyphae during the necrotrophic phase. Previous work using a monoclonal antibody, UB25, has identified a glycoprotein that is specific to the interfacial matrix that forms between the wall of the intracellular hypha and the invaginated host plasma membrane. The gene encoding the protein identified by UB25 was cloned by immunoscreening and designated CIH1. The predicted amino acid sequence revealed a proline-rich glycoprotein, and biochemical evidence suggested that it formed a cross-linked structure at the biotrophic interface. Although CIH1 is a fungal gene, its product has several similarities to plant cell wall proteins. In this paper, we have surveyed the distribution and expression of CIH1 within the genus Colletotrichum, encompassing both necrotrophic and hemibiotrophic species. The results show that homologues of the CIH1 gene are present in all the Colletotrichum species tested. Northern blot studies of the time course of the infection process in planta have shown that CIH1 is expressed by both C. lindemuthianum in bean and C. trifolii in alfalfa during the biotrophic phase of fungal development. Immunofluorescence labelling of infected epidermal strips with UB25 revealed that the intracellular hyphae formed by C. destructivum as it infects alfalfa were specifically labelled in a similar way to those formed by C. lindemuthianum in bean. Northern and Western analysis showed that CIH1 was also expressed by C. lindemuthianum in vitro, though not constitutively. |
| **Gene** | **Spearman** | **Description** | **Name in Scer** | **Description SGD** | **Relevant literature** |
| An09g06500 | 0,7 | Ortholog(s) have role in cellular response to drug, filamentous growth of a population of unicellular organisms | SGT1 | Sgt1p physically associates with Skp1p in vivo and in vitro, and thus also associates with SCF (Skp1p/Cdc53p/F box protein) ubiquitin ligase | [Sci Rep.](https://www.ncbi.nlm.nih.gov/pubmed/28139700) 2017 Jan 31;7:41626.  **The crystal structure of the Sgt1-Skp1 complex: the link between Hsp90 and both SCF E3 ubiquitin ligases and kinetochores.**  [Willhoft O](https://www.ncbi.nlm.nih.gov/pubmed/?term=Willhoft%20O%5BAuthor%5D&cauthor=true&cauthor_uid=28139700)1, [Kerr R](https://www.ncbi.nlm.nih.gov/pubmed/?term=Kerr%20R%5BAuthor%5D&cauthor=true&cauthor_uid=28139700)2, [Patel D](https://www.ncbi.nlm.nih.gov/pubmed/?term=Patel%20D%5BAuthor%5D&cauthor=true&cauthor_uid=28139700)1, [Zhang W](https://www.ncbi.nlm.nih.gov/pubmed/?term=Zhang%20W%5BAuthor%5D&cauthor=true&cauthor_uid=28139700)1, [Al-Jassar C](https://www.ncbi.nlm.nih.gov/pubmed/?term=Al-Jassar%20C%5BAuthor%5D&cauthor=true&cauthor_uid=28139700)1, [Daviter T](https://www.ncbi.nlm.nih.gov/pubmed/?term=Daviter%20T%5BAuthor%5D&cauthor=true&cauthor_uid=28139700)1, [Millson SH](https://www.ncbi.nlm.nih.gov/pubmed/?term=Millson%20SH%5BAuthor%5D&cauthor=true&cauthor_uid=28139700)3, [Thalassinos K](https://www.ncbi.nlm.nih.gov/pubmed/?term=Thalassinos%20K%5BAuthor%5D&cauthor=true&cauthor_uid=28139700)2, [Vaughan CK](https://www.ncbi.nlm.nih.gov/pubmed/?term=Vaughan%20CK%5BAuthor%5D&cauthor=true&cauthor_uid=28139700)1.  The essential cochaperone Sgt1 recruits Hsp90 chaperone activity to a range of cellular factors including SCF E3 ubiquitin ligases and the kinetochore in eukaryotes. In these pathways Sgt1 interacts with Skp1, a small protein that heterodimerizes with proteins containing the F-box motif. We have determined the crystal structure of the interacting domains of Saccharomyces cerevisiae Sgt1 and Skp1 at 2.8 Å resolution and validated the interface in the context of the full-length proteins in solution. The BTB/POZ domain of Skp1 associates with Sgt1 via the concave surface of its TPR domain using residues that are conserved in humans. Dimerization of yeast Sgt1 occurs via an insertion that is absent from monomeric human Sgt1. We identify point mutations that disrupt dimerization and Skp1 binding in vitro and find that the interaction with Skp1 is an essential function of Sgt1 in yeast. Our data provide a structural rationale for understanding the phenotypes of temperature-sensitive Sgt1 mutants and for linking Skp1-associated proteins to Hsp90-dependent pathways. |

| **Gene** | **Spearman** | **Description** | **Name in Scer** | **Description SGD** | **Relevant literature** |
| --- | --- | --- | --- | --- | --- |
| An08g10160 | -0,7 | Ortholog(s) have role in cellular response to farnesol, cellular response to heat, cellular response to hydrogen peroxide, sterigmatocystin biosynthetic process and peroxisome localization | /  DlpA (A. nidulans) | / | [J Proteomics.](https://www.ncbi.nlm.nih.gov/pubmed/?term=dlpA+nidulans) 2012 Jul 16;75(13):4038-49.  **Proteome analysis of the farnesol-induced stress response in Aspergillus nidulans--The role of a putative dehydrin.**  [Wartenberg D](https://www.ncbi.nlm.nih.gov/pubmed/?term=Wartenberg%20D%5BAuthor%5D&cauthor=true&cauthor_uid=22634043)1, [Vödisch M](https://www.ncbi.nlm.nih.gov/pubmed/?term=V%C3%B6disch%20M%5BAuthor%5D&cauthor=true&cauthor_uid=22634043), [Kniemeyer O](https://www.ncbi.nlm.nih.gov/pubmed/?term=Kniemeyer%20O%5BAuthor%5D&cauthor=true&cauthor_uid=22634043), [Albrecht-Eckardt D](https://www.ncbi.nlm.nih.gov/pubmed/?term=Albrecht-Eckardt%20D%5BAuthor%5D&cauthor=true&cauthor_uid=22634043), [Scherlach K](https://www.ncbi.nlm.nih.gov/pubmed/?term=Scherlach%20K%5BAuthor%5D&cauthor=true&cauthor_uid=22634043), [Winkler R](https://www.ncbi.nlm.nih.gov/pubmed/?term=Winkler%20R%5BAuthor%5D&cauthor=true&cauthor_uid=22634043), [Weide M](https://www.ncbi.nlm.nih.gov/pubmed/?term=Weide%20M%5BAuthor%5D&cauthor=true&cauthor_uid=22634043), [Brakhage AA](https://www.ncbi.nlm.nih.gov/pubmed/?term=Brakhage%20AA%5BAuthor%5D&cauthor=true&cauthor_uid=22634043).  The isoprenoid alcohol farnesol represents a quorum-sensing molecule in pathogenic yeasts, but was also shown to inhibit the growth of many filamentous fungi. In order to gain a deeper insight into the antifungal activity of farnesol, we performed 2D-differential gel electrophoretic analysis (2D-DIGE) of Aspergillus nidulans exposed to farnesol. We observed an increased abundance of antioxidative enzymes and proteins involved in protein folding and the ubiquitin-mediated protein degradation. A striking finding was the strong up-regulation of a dehydrin-like protein (DlpA). Expression analyses suggested the involvement of DlpA in the cellular response to oxidative, osmotic and cold stress. In line with these data, we demonstrated that dlpA expression was regulated by the MAP kinase SakA/HogA. The generation of both a dlpA Tet(on) antisense RNA-producing A. nidulans strain (dlpA-inv) and a ΔdlpA deletion mutant indicated a role of DlpA in conidiation and stress resistance of dormant conidia against heat and ROS. Furthermore, the production of the secondary metabolite sterigmatocystin was absent in both strains dlpA-inv and ΔdlpA. Our results demonstrate the complexity of the farnesol-mediated stress response in A. nidulans and describe a farnesol-inducible dehydrin-like protein that contributes to the high tolerance of resting conidia against oxidative and heat stress. |

| **Gene** | **Spearman** | **Description** | **Name in Scer** | **Description SGD** | **Relevant literature** |
| --- | --- | --- | --- | --- | --- |
| An03g05250 | 0,7 | Has domain(s) with predicted role in transmembrane transport and integral component of membrane localization | THI73 | Putative plasma membrane permease; proposed to be involved in carboxylic acid uptake and repressed by thiamine; substrate of Dbf2p/Mob1p kinase; transcription is altered if mitochondrial dysfunction occurs | [Mol Genet Genomics.](https://www.ncbi.nlm.nih.gov/pubmed/16850348) 2006 Aug;276(2):147-61. Epub 2006 Jun 1.  **Pdc2 coordinates expression of the THI regulon in the yeast Saccharomyces cerevisiae.**  [Mojzita D](https://www.ncbi.nlm.nih.gov/pubmed/?term=Mojzita%20D%5BAuthor%5D&cauthor=true&cauthor_uid=16850348)1, [Hohmann S](https://www.ncbi.nlm.nih.gov/pubmed/?term=Hohmann%20S%5BAuthor%5D&cauthor=true&cauthor_uid=16850348).  Coordination of gene expression in response to different metabolic signals is crucial for cellular homeostasis. In this work, we addressed the role of Pdc2 in the coordinated control of biosynthesis and demand of an essential metabolic cofactor, thiaminediphosphate (ThDP). The DNA binding protein Pdc2 was initially identified as a regulator of the genes PDC1 and PDC5, which encode isoforms of the glycolytic enzyme pyruvate decarboxylase (Pdc). The Pdc2 has also been implicated as a regulator of genes encoding enzymes in ThDP metabolism. The ThDP is the cofactor of Pdc. Using global and gene-specific expression analysis, we show that Pdc2 is required for the upregulation of all genes controlled by thiamine availability. The Pdc2 seems to act together with Thi2, a known transcriptional regulator of THI genes. The requirement for these two factors differs in a gene-specific manner. While the Thi2, in conjunction with Thi3, seems to control expression of THI genes with respect to thiamine availability, the Pdc2 may link the ThDP demand to carbon source availability. Interestingly, the enzymes Pdc1 and Pdc5 are enriched in the nucleus. Both are known to affect gene expression in an autoregulatory mechanism and expression of both is regulated by glucose and Pdc2, further pointing to a role of Pdc2 in coordinating different metabolic signals. Our analysis helps to further define the THI regulon and hence the spectrum of genes/proteins involved in the ThDP homeostasis. In particular, we identify novel proteins putatively involved in thiamine and/or ThDP transport across the plasma and the mitochondrial membrane. In conclusion, the THI regulon is the most interesting system to study principles of genes expression and metabolic coordination and deserves further attention.  [**Partial Decay of Thiamine Signal Transduction Pathway Alters Growth Properties of Candida glabrata.**](https://www.ncbi.nlm.nih.gov/pubmed/27015653)  Iosue CL, Attanasio N, Shaik NF, Neal EM, Leone SG, Cali BJ, Peel MT, Grannas AM, Wykoff DD.  PLoS One. 2016 Mar 25;11(3):e0152042. |

| **Gene** | **Spearman** | **Description** | **Name in Scer** | **Description SGD** | **Relevant literature** |
| --- | --- | --- | --- | --- | --- |
| An07g01090 | 0,75 | Has domain(s) with predicted role in transmembrane transport and integral component of membrane localization | FLR1 | Plasma membrane transporter of the major facilitator superfamily | From SGD:  The widespread biological phenomenon of multidrug resistance (MDR) poses serious challenges for the treatment of human cancers, and also of bacterial and fungal infections. MDR is typically associated with [transport](https://www.yeastgenome.org/go/6855) systems that catalyze the [efflux](https://www.yeastgenome.org/go/15562) of various compounds out of the cell. Among the most important MDR transporters are those belonging to the major facilitator superfamily (MFS). MFS-MDR transporters are found in Eucarya, Bacteria, and Archaea, and have been classified into two families based on the number of predicted transmembrane spans: The Drug:H+ Antiporter-1 (12-Spanner; DHA1) Family, TC 2.A.1.2, and the Drug:H+ Antiporter-2 (14-Spanner; DHA2) Family, TC 2.A.1.3 ([7](https://www.yeastgenome.org/reference/S000115378)).  In S. cerevisiae, the DHA1 family comprises 12 genes involved in various biological processes: [AQR1](https://www.yeastgenome.org/locus/S000005009), [QDR1](https://www.yeastgenome.org/locus/S000001382), [QDR2](https://www.yeastgenome.org/locus/S000001383), [QDR3](https://www.yeastgenome.org/locus/S000000247), [FLR1](https://www.yeastgenome.org/locus/S000000212), [DTR1](https://www.yeastgenome.org/locus/S000000384), [TPO1](https://www.yeastgenome.org/locus/S000003951), [TPO2](https://www.yeastgenome.org/locus/S000003370), [TPO3](https://www.yeastgenome.org/locus/S000006360), [TPO4](https://www.yeastgenome.org/locus/S000005799), [HOL1](https://www.yeastgenome.org/locus/S000005338), and [YHK8](https://www.yeastgenome.org/locus/S000001090) ([7](https://www.yeastgenome.org/reference/S000115378)). [Aqr1p](https://www.yeastgenome.org/locus/S000005009), Qdr1-3p, and [Flr1p](https://www.yeastgenome.org/locus/S000000212) are [plasma membrane](https://www.yeastgenome.org/go/5886) proteins that serve as [multidrug transporters](https://www.yeastgenome.org/go/15238) ([8](https://www.yeastgenome.org/reference/S000069477), [9](https://www.yeastgenome.org/reference/S000060349), [10](https://www.yeastgenome.org/reference/S000080433), [11](https://www.yeastgenome.org/reference/S000076810), [12](https://www.yeastgenome.org/reference/S000066144), [1](https://www.yeastgenome.org/reference/S000052308), [2](https://www.yeastgenome.org/reference/S000042127)). [Aqr1p](https://www.yeastgenome.org/locus/S000005009) has also been implicated in the [excretion of excess amino acids](https://www.yeastgenome.org/go/32973), and [Qdr2p](https://www.yeastgenome.org/locus/S000001383) in the [import of potassium ions](https://www.yeastgenome.org/go/10107) ([13](https://www.yeastgenome.org/reference/S000079962), [14](https://www.yeastgenome.org/reference/S000120124)). [Dtr1p](https://www.yeastgenome.org/locus/S000000384), a putative dityrosine transporter, resides in the [prospore membrane](https://www.yeastgenome.org/go/5628) and functions in [spore wall synthesis](https://www.yeastgenome.org/go/30476) ([15](https://www.yeastgenome.org/reference/S000071740), [16](https://www.yeastgenome.org/reference/S000081076)). Tpo1-4p are membrane proteins involved in the [export of polyamines](https://www.yeastgenome.org/go/15846), including spermine, spermidine, and putrescine ([17](https://www.yeastgenome.org/reference/S000066148), [18](https://www.yeastgenome.org/reference/S000072984)). [Hol1p](https://www.yeastgenome.org/locus/S000005338) participates in [cation](https://www.yeastgenome.org/go/6812) and [alcohol transport](https://www.yeastgenome.org/go/15850) ([19](https://www.yeastgenome.org/reference/S000043679), [20](https://www.yeastgenome.org/reference/S000051592)), and [Yhk8p](https://www.yeastgenome.org/locus/S000001090) is a putative drug transporter requiring further experimental characterization ([12](https://www.yeastgenome.org/reference/S000066144), [21](https://www.yeastgenome.org/reference/S000073301)). |

| **Gene** | **Spearman** | **Description** | **Name in Scer** | **Description SGD** | **Relevant literature** |
| --- | --- | --- | --- | --- | --- |
| An03g00430 | 0,7 | Has domain(s) with predicted amino acid transmembrane transporter activity, role in amino acid transmembrane transport and membrane localization | HNM1 | [HNM1](https://www.yeastgenome.org/locus/S000003045) encodes a high affinity Plasma membrane transporter involved in the active [transport of choline](https://www.yeastgenome.org/go/15871) and [ethanolamine](https://www.yeastgenome.org/go/34229), which are used as precursors for the [biosynthesis of phosphatidylcholine](https://www.yeastgenome.org/go/6656) and [phosphatidylethanolamine](https://www.yeastgenome.org/go/6646)during hypersaline stress; co-regulated with phospholipid biosynthetic genes and negatively regulated by choline and myo-inositol | [FEMS Yeast Res.](https://www.ncbi.nlm.nih.gov/pubmed/26895788) 2016 May;16(3).  **Alternative reactions at the interface of glycolysis and citric acid cycle in Saccharomyces cerevisiae.**  [van Rossum HM](https://www.ncbi.nlm.nih.gov/pubmed/?term=van%20Rossum%20HM%5BAuthor%5D&cauthor=true&cauthor_uid=26895788)1, [Kozak BU](https://www.ncbi.nlm.nih.gov/pubmed/?term=Kozak%20BU%5BAuthor%5D&cauthor=true&cauthor_uid=26895788)1, [Niemeijer MS](https://www.ncbi.nlm.nih.gov/pubmed/?term=Niemeijer%20MS%5BAuthor%5D&cauthor=true&cauthor_uid=26895788)1, [Duine HJ](https://www.ncbi.nlm.nih.gov/pubmed/?term=Duine%20HJ%5BAuthor%5D&cauthor=true&cauthor_uid=26895788)1, [Luttik MA](https://www.ncbi.nlm.nih.gov/pubmed/?term=Luttik%20MA%5BAuthor%5D&cauthor=true&cauthor_uid=26895788)1, [Boer VM](https://www.ncbi.nlm.nih.gov/pubmed/?term=Boer%20VM%5BAuthor%5D&cauthor=true&cauthor_uid=26895788)2, [Kötter P](https://www.ncbi.nlm.nih.gov/pubmed/?term=K%C3%B6tter%20P%5BAuthor%5D&cauthor=true&cauthor_uid=26895788)3, [Daran JM](https://www.ncbi.nlm.nih.gov/pubmed/?term=Daran%20JM%5BAuthor%5D&cauthor=true&cauthor_uid=26895788)1, [van Maris AJ](https://www.ncbi.nlm.nih.gov/pubmed/?term=van%20Maris%20AJ%5BAuthor%5D&cauthor=true&cauthor_uid=26895788)1, [Pronk JT](https://www.ncbi.nlm.nih.gov/pubmed/?term=Pronk%20JT%5BAuthor%5D&cauthor=true&cauthor_uid=26895788)4.  Pyruvate and acetyl-coenzyme A, located at the interface between glycolysis and TCA cycle, are important intermediates in yeast metabolism and key precursors for industrially relevant products. Rational engineering of their supply requires knowledge of compensatory reactions that replace predominant pathways when these are inactivated. This study investigates effects of individual and combined mutations that inactivate the mitochondrial pyruvate-dehydrogenase (PDH) complex, extramitochondrial citrate synthase (Cit2) and mitochondrial CoA-transferase (Ach1) in Saccharomyces cerevisiae. Additionally, strains with a constitutively expressed carnitine shuttle were constructed and analyzed. A predominant role of the PDH complex in linking glycolysis and TCA cycle in glucose-grown batch cultures could be functionally replaced by the combined activity of the cytosolic PDH bypass and Cit2. Strongly impaired growth and a high incidence of respiratory deficiency in pda1Δ ach1Δ strains showed that synthesis of intramitochondrial acetyl-CoA as a metabolic precursor requires activity of either the PDH complex or Ach1. Constitutive overexpression of AGP2, HNM1, YAT2, YAT1, CRC1 and CAT2 enabled the carnitine shuttle to efficiently link glycolysis and TCA cycle in l-carnitine-supplemented, glucose-grown batch cultures. Strains in which all known reactions at the glycolysis-TCA cycle interface were inactivated still grew slowly on glucose, indicating additional flexibility at this key metabolic junction. |

| **Gene** | **Spearman** | **Description** | **Name in Scer** | **Description SGD** | **Relevant literature** |
| --- | --- | --- | --- | --- | --- |
| An13g02810 | -0,7 | Has domain(s) with predicted role in transmembrane transport and integral component of membrane localization | ENB1 | [Endosomal ferric enterobactin transporter; expressed under conditions of iron deprivation; member of the major facilitator superfamily; expression is regulated by Rcs1p and affected by chloroquine treatment](https://www.yeastgenome.org/locus/S000005518#reference) | [Biochem Soc Trans.](https://www.ncbi.nlm.nih.gov/pubmed/12196168) 2002 Aug;30(4):698-702.  **The response to iron deprivation in Saccharomyces cerevisiae: expression of siderophore-based systems of iron uptake.**  [Philpott CC](https://www.ncbi.nlm.nih.gov/pubmed/?term=Philpott%20CC%5BAuthor%5D&cauthor=true&cauthor_uid=12196168)1, [Protchenko O](https://www.ncbi.nlm.nih.gov/pubmed/?term=Protchenko%20O%5BAuthor%5D&cauthor=true&cauthor_uid=12196168), [Kim YW](https://www.ncbi.nlm.nih.gov/pubmed/?term=Kim%20YW%5BAuthor%5D&cauthor=true&cauthor_uid=12196168), [Boretsky Y](https://www.ncbi.nlm.nih.gov/pubmed/?term=Boretsky%20Y%5BAuthor%5D&cauthor=true&cauthor_uid=12196168), [Shakoury-Elizeh M](https://www.ncbi.nlm.nih.gov/pubmed/?term=Shakoury-Elizeh%20M%5BAuthor%5D&cauthor=true&cauthor_uid=12196168).  The budding yeast Saccharomyces cerevisiae responds to growth in limiting amounts of iron by activating the transcription factor Aft1p and expressing a set of genes that ameliorate the effects of iron deprivation. Analysis of iron-regulated gene expression using cDNA microarrays has revealed the set of genes controlled by iron and Aft1p. Many of these genes are involved in the uptake of siderophore-bound iron from the environment. One family of genes, FIT1, FIT2 and FIT3, codes for mannoproteins that are incorporated into the cell wall via glycosylphosphatidylinositol anchors. These genes are involved in the retention of siderophore-iron in the cell wall. Siderophore-bound iron can be taken up into the cell via two genetically separable systems. One system requires the reduction and release of the iron from the siderophore prior to uptake by members of the Fre family of plasma-membrane metalloreductases. Following reduction and release from the siderophore, the iron is then taken up via the high-affinity ferrous transport system. A set of transporters that specifically recognizes siderophore-iron chelates is also expressed under conditions of iron deprivation. These transporters, encoded by ARN1, ARN2/TAF1, ARN3/SIT1 and ARN4/ENB1, facilitate the uptake of both hydroxamate- and catecholate-type siderophores. The Arn transporters are expressed in intracellular vesicles that correspond to the endosomal compartment, which suggests that intracellular trafficking of the siderophore and/or its transporter may be important for uptake. |
