# Supplementary material for: A Library of Aspergillus niger Chassis Strains for Morphology Engineering Connects Strain Fitness and Filamentous Growth With Submerged Macromorphology
Source: Front Bioeng Biotechnol. 2022 Jan 17;9:820088. doi: 10.3389/fbioe.2021.820088 (PMC8801610; doi:10.3389/fbioe.2021.820088)
Supplement: Supplementary file 6 [file DataSheet2.DOCX]

**A library of *Aspergillus niger* chassis strains for morphology engineering connects strain fitness and filamentous growth with submerged macromorphology**

**Timothy C. Cairns^1^, Xiaomei Zheng^2.3.4.5^Claudia Feurstein^1^, Ping Zheng^2,3,4,5^, Jibin Sun^2,3,4,5^, and Vera Meyer^1^**

^1^Technische Universität Berlin, Institute of Biotechnology, Chair of Applied and Molecular Microbiology,

Straße des 17. Juni 135, 10623 Berlin, Germany

^2^ Tianjin Institute of Industrial Biotechnology, Chinese Academy of Sciences, Tianjin, 300308, People’s Republic of China

^3^ Key Laboratory of Systems Microbial Biotechnology, Chinese Academy of Sciences, Tianjin 300308, People’s Republic of China

^4^ University of Chinese Academy of Sciences, Beijing, 100049 China

^5^ College of Biotechnology, Tianjin University of Science & Technology, Tianjin, 300457 China

Timothy C. Cairns: [t.cairns@tu-berlin.de](mailto:t.cairns@tu-berlin.de) ORCID: 0000-0001-7106-224X

Xiaomei Zheng: [zheng_xm@tib.cas.cn](mailto:zheng_xm@tib.cas.cn) ORCID: 0000-0001-9136-0666

Claudia Feurstein: [c.feurstein@tu-berlin.de](mailto:c.feurstein@tu-berlin.de): ORCID: 0000-0001-7046-4183

Ping Zheng: [zheng_p@tib.cas.cn](mailto:zheng_p@tib.cas.cn): ORCID: 0000-0001-9434-9892

Jibin Sun: [sun_jb@tib.cas.cn](mailto:sun_jb@tib.cas.cn): ORCID: 0000-0002-0208-504X

Vera Meyer: [vera.meyer@tu-berlin.de](mailto:vera.meyer@tu-berlin.de), ORCID 0000-0002-2298-2258

**Contact details for corresponding authors:**

Timothy C. Cairns, Tel.: +49 30 314 72750, Fax: +49 30 314 72922, E-mail: [t.cairns@tu-berlin.de](mailto:t.cairns@tu-berlin.de)

Jibin Sun, Tel.: +86-8486 1949, Fax: +86-8486 1943, E-mail: [sun_jb@tib.cas.cn](mailto:sun_jb@tib.cas.cn)

Vera Meyer, Tel.: +49 30 314 72750, Fax: +49 30 314 72922, E-mail: [vera.meyer@tu-berlin.de](mailto:vera.meyer@tu-berlin.de)


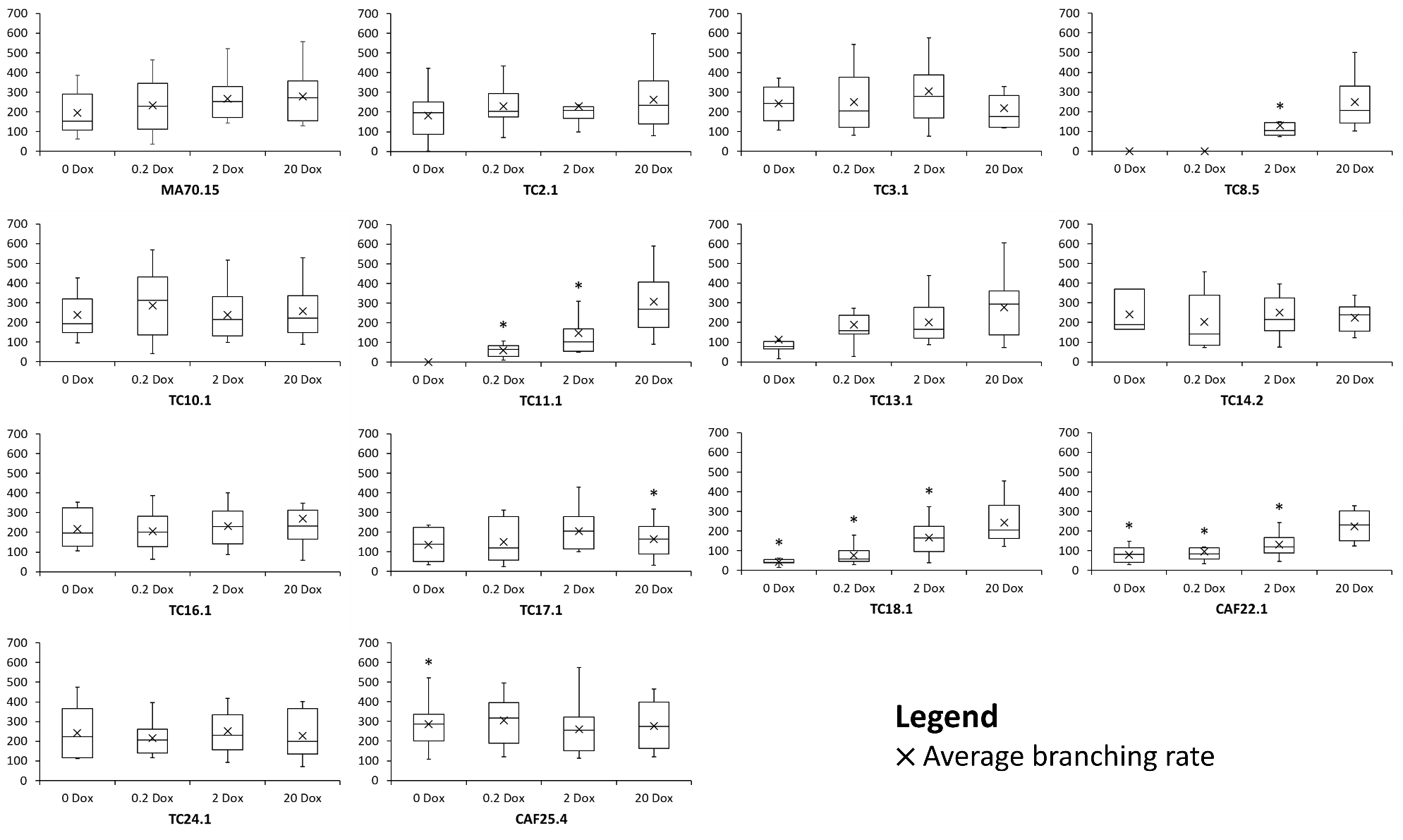


**Supplemental File 2: Box whisker blots of hyphal branch rates in the conditional expression mutants following titration of gene expression.** Branching was calculated as total length of hyphae (µm)/ number of branches
