# Supplementary material for: A Library of Aspergillus niger Chassis Strains for Morphology Engineering Connects Strain Fitness and Filamentous Growth With Submerged Macromorphology
Source: Front Bioeng Biotechnol. 2022 Jan 17;9:820088. doi: 10.3389/fbioe.2021.820088 (PMC8801610; doi:10.3389/fbioe.2021.820088)
Supplement: Supplementary file 7 [file DataSheet1.DOCX]

**A library of *Aspergillus niger* chassis strains for morphology engineering connects strain fitness and filamentous growth with submerged macromorphology**

**Timothy C. Cairns^1^, Xiaomei Zheng^2.3.4.5^Claudia Feurstein^1^, Ping Zheng^2,3,4,5^, Jibin Sun^2,3,4,5^, and Vera Meyer^1^**

^1^Technische Universität Berlin, Institute of Biotechnology, Chair of Applied and Molecular Microbiology,

Straße des 17. Juni 135, 10623 Berlin, Germany

^2^ Tianjin Institute of Industrial Biotechnology, Chinese Academy of Sciences, Tianjin, 300308, People’s Republic of China

^3^ Key Laboratory of Systems Microbial Biotechnology, Chinese Academy of Sciences, Tianjin 300308, People’s Republic of China

^4^ University of Chinese Academy of Sciences, Beijing, 100049 China

^5^ College of Biotechnology, Tianjin University of Science & Technology, Tianjin, 300457 China

Timothy C. Cairns: [t.cairns@tu-berlin.de](mailto:t.cairns@tu-berlin.de) ORCID: 0000-0001-7106-224X

Xiaomei Zheng: [zheng_xm@tib.cas.cn](mailto:zheng_xm@tib.cas.cn) ORCID: 0000-0001-9136-0666

Claudia Feurstein: [c.feurstein@tu-berlin.de](mailto:c.feurstein@tu-berlin.de): ORCID: 0000-0001-7046-4183

Ping Zheng: [zheng_p@tib.cas.cn](mailto:zheng_p@tib.cas.cn): ORCID: 0000-0001-9434-9892

Jibin Sun: [sun_jb@tib.cas.cn](mailto:sun_jb@tib.cas.cn): ORCID: 0000-0002-0208-504X

Vera Meyer: [vera.meyer@tu-berlin.de](mailto:vera.meyer@tu-berlin.de), ORCID 0000-0002-2298-2258

**Contact details for corresponding authors:**

Timothy C. Cairns, Tel.: +49 30 314 72750, Fax: +49 30 314 72922, E-mail: [t.cairns@tu-berlin.de](mailto:t.cairns@tu-berlin.de)

Jibin Sun, Tel.: +86-8486 1949, Fax: +86-8486 1943, E-mail: [sun_jb@tib.cas.cn](mailto:sun_jb@tib.cas.cn)

Vera Meyer, Tel.: +49 30 314 72750, Fax: +49 30 314 72922, E-mail: [vera.meyer@tu-berlin.de](mailto:vera.meyer@tu-berlin.de)

**
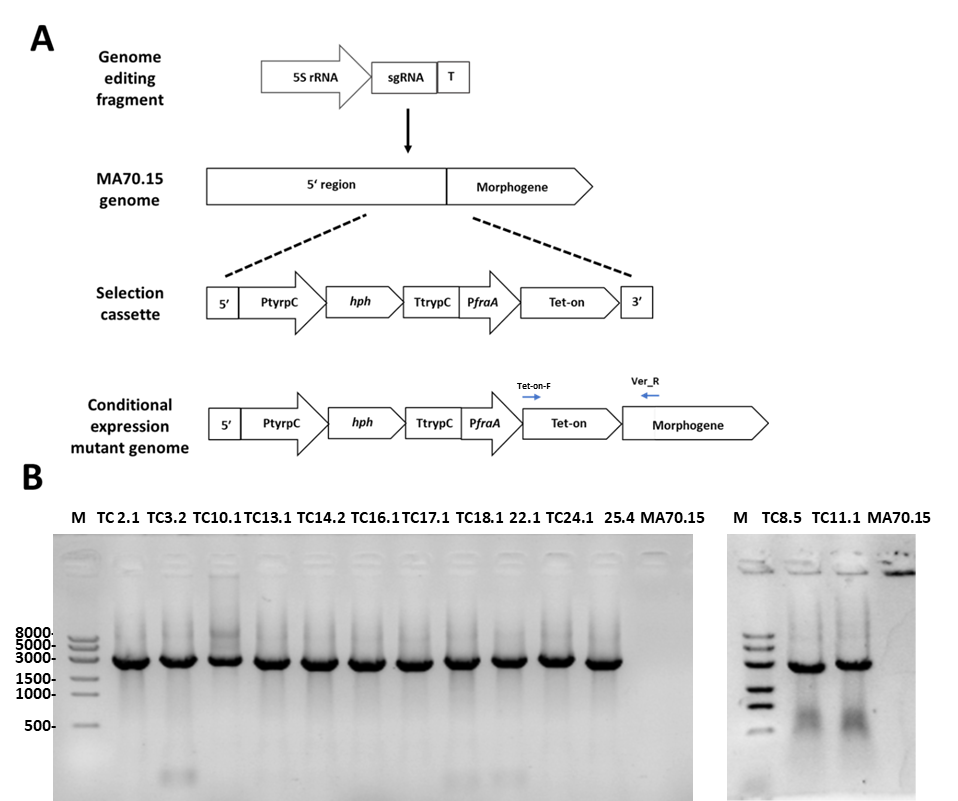
**

**Figure S1.1: Mutant construction and exemplar images of PCR mutant screening.** Schematic diagram depicts genome editing and targeting of the Tet-on cassette in a recipient genome (not to scale) (A). An amplicon ~2.8kb following PCR using Tet-on-F and Ver_R primer pair (Supplemental Table 4) indicates integration of the cassette at the target locus (B).

**
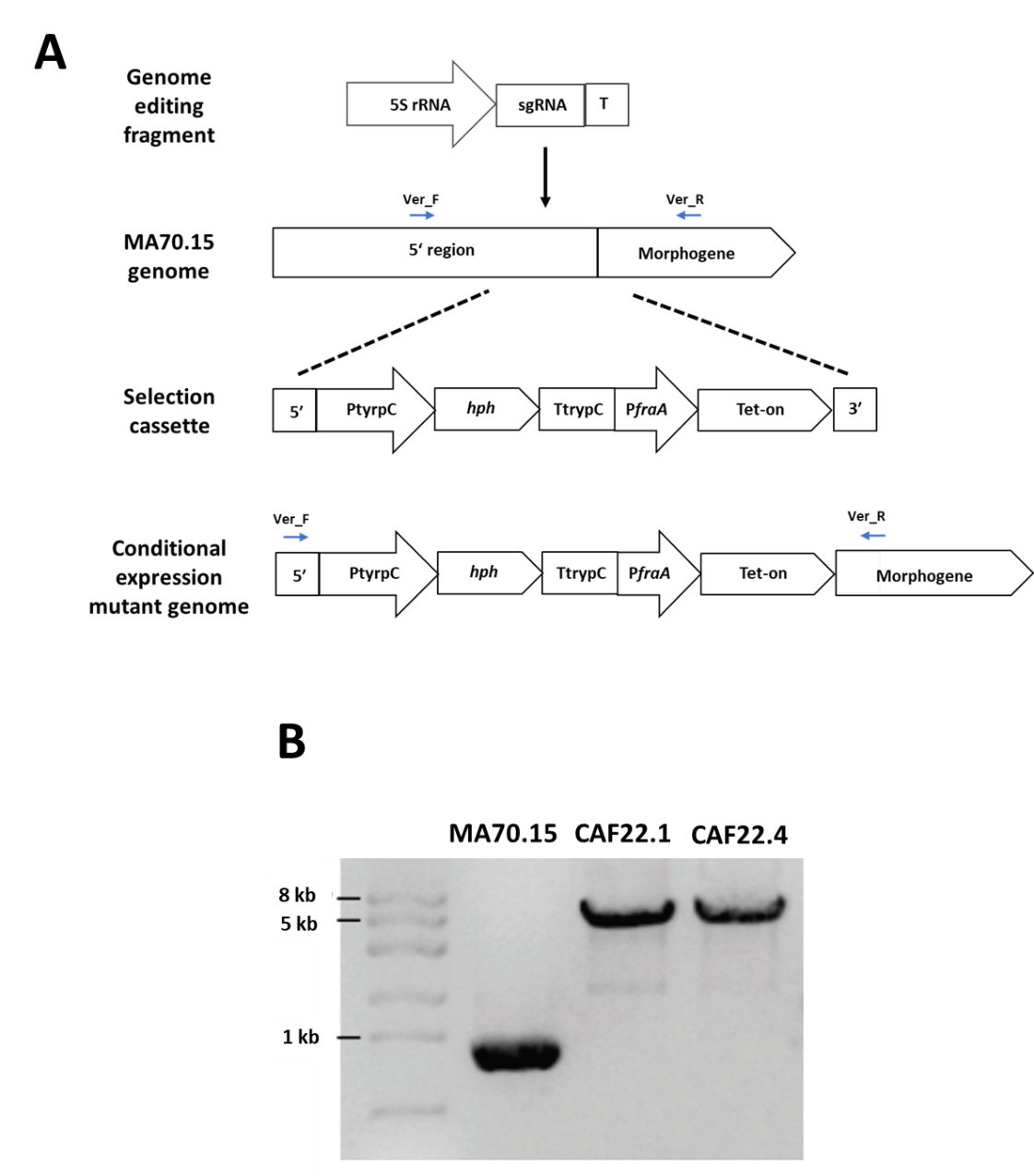
**

**Figure S1.2: Mutant construction and exemplar images of PCR mutant screening.** Schematic diagram depicts genome editing and targeting of the Tet-on cassette in a recipient genome (not to scale) (A). An increase in amplicon size following PCR using verification primers (Supplemental Table 4) and genomic DNA extracted from the mutant relative to the progenitor control occurs due to integration of the Tet-on cassette at this locus (exemplar strains CAF22.1/CAF22.4 given in B). All PCR verification data will be made available on request.

**Supplemental File 1: Southern blot confirmation of conditional expression mutants**

A 1 kb portion of the *fraA* promoter locus (An16g04690) was DIG labelled by PCR amplification using primers VM_554 (ccctcggctggtctgtctta) and VM_784 (tttggcggtttgttgctggc). Genomic DNA from progenitor strain MA70.15 or conditional expression mutants was extracted and digested with PvuII and HindIII. During Southern blot hybridisation, the native An16g04690 locus results in a 2.3 kb fragment which served as a positive control for DNA integrity. The addition of the *fraA* promoter present in the Tet-on cassette results in an addition band of predicted size indicated in Table 1 of this Supplemental File.

| **Strain** | **Predicted hybridisation size of native *fraA* promoter** | **Predicted hybridisation size following integration of Tet-on at the target locus** |
| --- | --- | --- |
| **MA70.15** | 2353 | NONE |
| **TC2.1** | 2353 | 6344 |
| **TC3.2** | 2353 | 5326 |
| **TC8.5** | 2353 | 6956 |
| **TC10.1** | 2353 | 5907 |
| **TC11.1** | 2353 | 6956 |
| **TC13.1** | 2353 | 8428 |
| **TC14.2** | 2353 | 8766 |
| **TC16.1** | 2353 | 5601 |
| **TC17.1** | 2353 | 6327 |
| **TC18.1** | 2353 | 6086 |
| **CAF22.1** | 2353 | 10810 |
| **TC24.1** | 2353 | 9599 |
| **CAF25.4** | 2353 | 6011 |

Table S1: Predicted sizes of Southern blot probe hybridization using the DIG labelled *fraA* probe

**Supplemental File Figure S1.3: Chemiluminescent detected Southern blot analysis of indicated strains.** Note lanes depicting isolates not used in this study are left unlabelled.

**
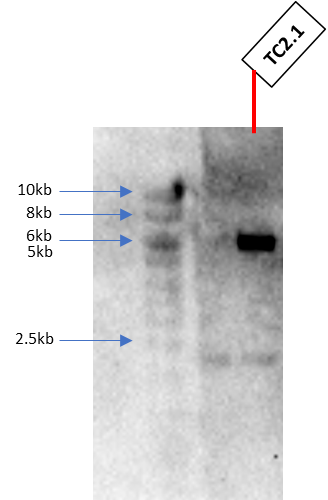
**

**Supplemental File Figure 1.4: Chemiluminescent detected Southern blot analysis of indicated strains.** Note lanes depicting isolates not used in this study are left unlabelled.

**
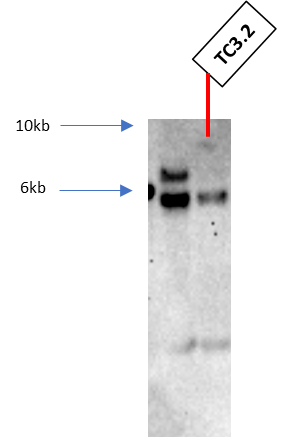
**

**Supplemental File Figure 1.5: Chemiluminescent detected Southern blot analysis of indicated strains.** Note lanes depicting isolates not used in this study are left unlabelled.

**
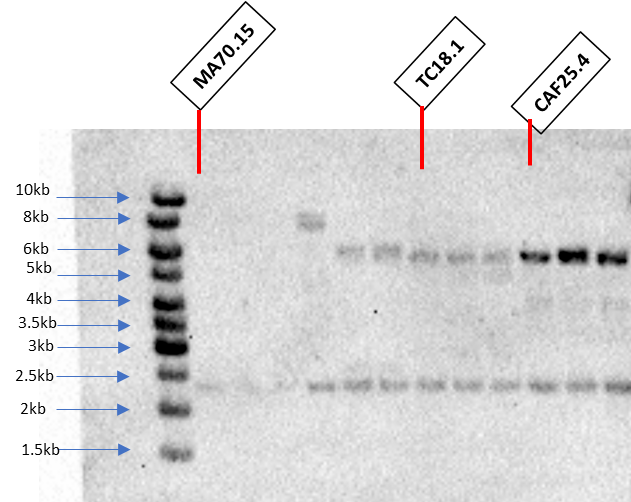
**

**Supplemental File Figure 1.6: Chemiluminescent detected Southern blot analysis of indicated strains.** Note lanes depicting isolates not used in this study are left unlabelled.

**
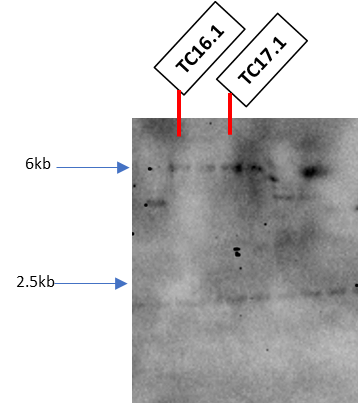
**

**Supplemental File Figure 1.7: Chemiluminescent detected Southern blot analysis of indicated strains.** Note lanes depicting isolates not used in this study are left unlabelled.

**
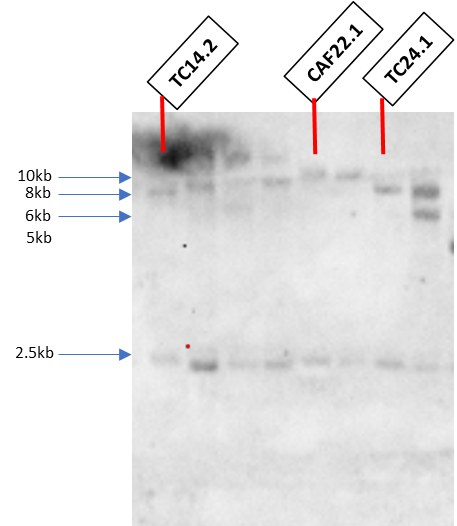
**

**Supplemental File Figure 1.8: Chemiluminescent detected Southern blot analysis of indicated strains.** Note lanes depicting isolates not used in this study are left unlabelled.

**
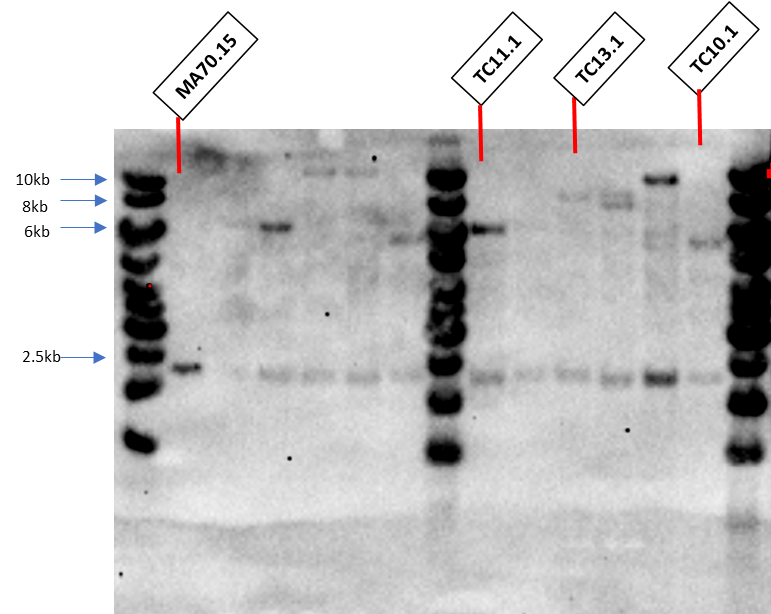
**

**Supplemental File Figure 1.9: Chemiluminescent detected Southern blot analysis of indicated strains.** Note lanes depicting isolates not used in this study are left unlabelled. Note- over-exposed lane in blot is from plasmid pFW22.1 control which contains the *fraA* promoter.

**
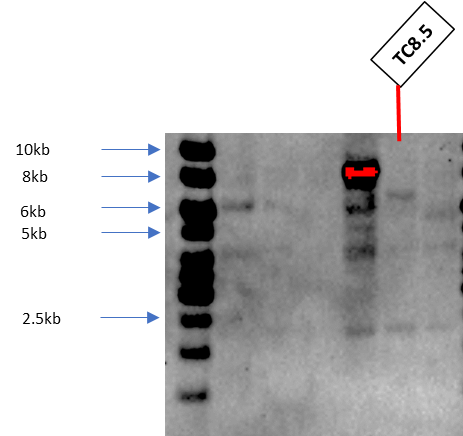
**
